# CD63 aptamer–based electrochemical sensor assesses stored red blood cell–derived exosome burden associated with mast-cell–mediated transfusion-related acute lung injury

**DOI:** 10.1186/s12951-026-04917-x

**Published:** 2026-08-12

**Authors:** Ling Zheng, Shan-Shan Su, Sai-nan Qin, Yimin Ren, Danfeng Wang, Pingting Huang, Xiaochun Zheng, Jian-Jun Sun, Xiaobin Fang

**Affiliations:** 1 Department of Anesthesiology/Critical Care Medicine, School of Medicine, Fujian Provincial Key Laboratory of Critical Care Medicine, Fuzhou University Affiliated Provincial Hospital, Fuzhou University, Shengli Clinical Medical College of Fujian Medical University, Fujian Provincial Hospital, Fuzhou, 350001 Fujian China; 2 Ministry of Education Key Laboratory for Analytical Science of Food Safety and Biology, College of Chemistry, Fuzhou University Affiliated Provincial Hospital, Fuzhou University, Fuzhou, 350116 China; 3 Department of Anesthesiology, the Affiliated Hospital of Guizhou Medical University, Guiyang, China; 4 Department of Emergency Medicine, Fujian Provincial Hospital, Fujian Provincial Key Laboratory of Emergency Medicine, Shengli Clinical College of Fujian Medical University, Fuzhou University Affiliated Provincial Hospital, 34 Dongjie, Gulou District, Fuzhou, 350001 Fujian China; 5 Department of Anesthesiology, Fujian Provincial Hospital, Shengli Clinical Medical College of Fujian Medical University & Fujian Emergency Medical Center, Fujian Provincial Key Laboratory of Emergency Medicine, Fujian Provincial Key Laboratory of Critical Medicine, Fujian Provincial Co-constructed Laboratory of “Belt and Road,”, Fuzhou, Fujian China

**Keywords:** Exosomes, Electrochemical aptamer-based sensors, Mast cells, Rapid detection, Transfusion-related acute lung injury

## Abstract

Transfusion-related acute lung injury (TRALI) remains a serious complication of transfusion, and blood component–derived exosomes have been proposed as candidate contributors to transfusion-related inflammatory dysregulation. Rapid and practical methods to assess exosome burden in stored red blood cell (RBC) units remain limited. Here, we developed a CD63 electrochemical aptamer-based (E-AB) sensor as a preliminary analytical platform to rapidly detect exosome-associated signals and investigated whether stored red-blood-cell–derived exosomes (EXs-RBC) contribute to mast-cell–associated inflammatory lung injury in TRALI-relevant models. Using the CD63 E-AB sensor, we assessed EXs-RBC signals in stored RBC units, isolated EXs-RBC for HMC-1 cell stimulation and mouse experiments, and evaluated their inflammatory effects. The CD63 E-AB readout decreased in a concentration-dependent manner with increasing exosome levels. EXs-RBC dose-dependently amplified inflammatory responses, and these effects were attenuated by pharmacologic mast-cell inhibition or mast-cell deficiency. In antibody-independent TRALI-relevant inflammatory lung injury models and a supportive antibody-mediated TRALI-like model, EXs-RBC exacerbated lung injury and were associated with activation of the mast-cell toll-like receptor 3 (TLR3)–mitogen-activated protein kinase (MAPK) signaling. Collectively, these findings support the feasibility of using a CD63 E-AB sensor to assess exosome burden in stored RBC units and provide experimental evidence that EXs-RBC can amplify mast-cell-associated inflammatory lung injury in TRALI-relevant models, supporting further investigation of EXs-RBC burden as a candidate donor-side metric in transfusion safety research.

**Graphical abstract:**

**Figure:**
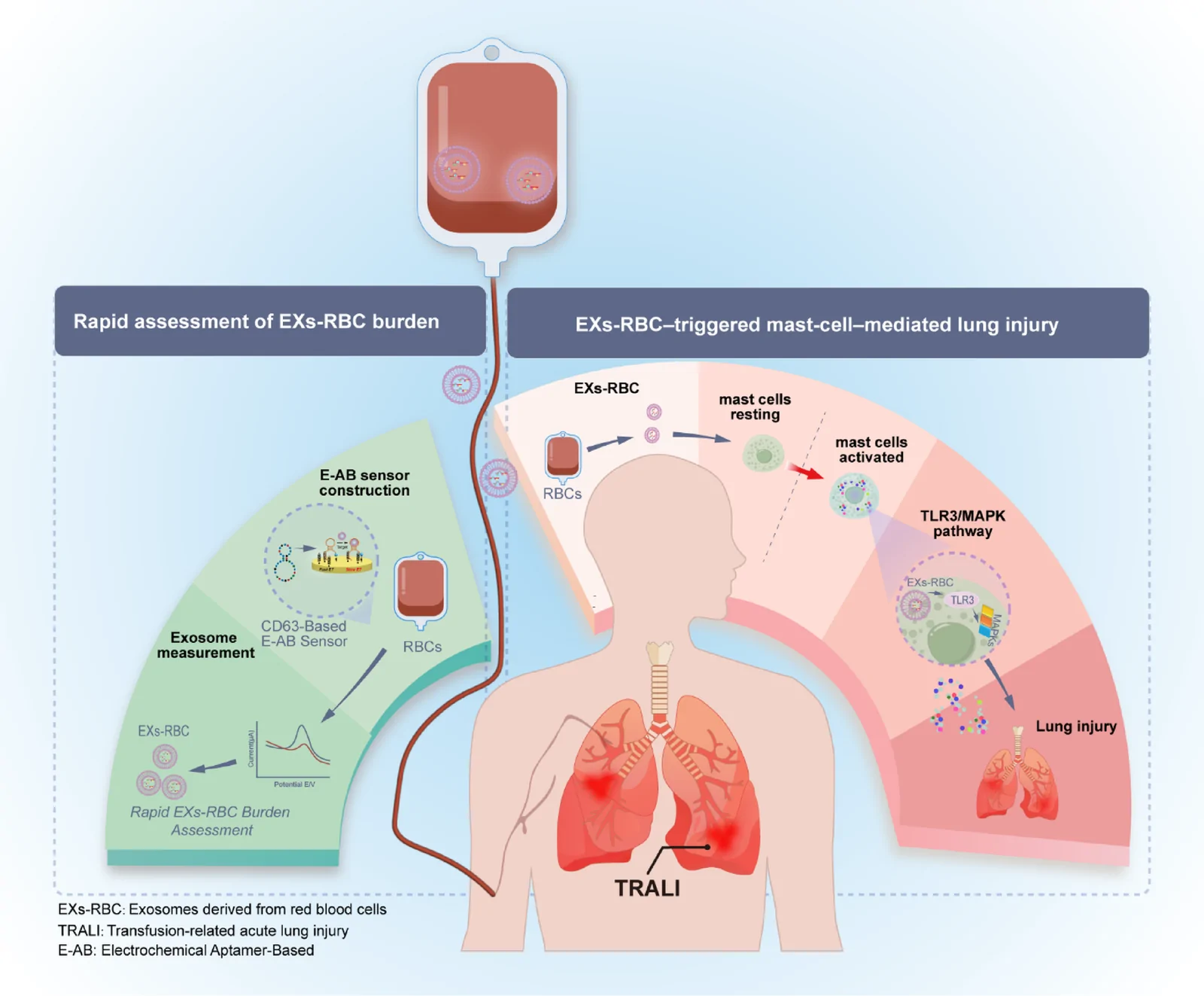

**Supplementary Information:**

The online version contains supplementary material available at https://doi.org/10.1186/s12951-026-04917-x.

## Introduction

Blood transfusion is indispensable to clinical care, yet ensuring transfusion safety remains a global challenge [1]. Transfusion-related acute lung injury (TRALI) typically occurs within 6 h of transfusion and is defined by acute hypoxemic respiratory failure with noncardiogenic pulmonary edema and bilateral pulmonary infiltrates [2–4]; it remains a major threat to transfusion safety and a leading cause of transfusion‑associated mortality [5, 6]. High mortality is primarily attributed to the lack of effective prevention and treatment, reflecting the incompletely defined pathogenesis of TRALI. This pathogenesis is commonly framed within a two-hit model, in which recipient susceptibility provides the first hit and transfusion-related factors provide the second hit [7–10]. This second hit may include donor antibodies as well as other biologically active mediators that amplify pulmonary inflammation in a primed host [11]. This pulmonary inflammation is increasingly understood as a multicellular process involving neutrophils, monocytes/macrophages, platelets, complement activation, and the pulmonary endothelium [12–19]. Within this multicellular inflammatory process, immune-cell and complement activation converge on the alveolar–capillary barrier, particularly the pulmonary endothelium, thereby promoting barrier dysfunction in TRALI-related acute lung injury [20, 21]. Against this pathophysiologic background, identifying and quantifying bioactive transfusion-related mediators that may constitute the second hit is important for clarifying TRALI mechanisms and may support future transfusion-safety strategies.

Among bioactive transfusion-related mediators, donor antibodies are well-recognized triggers of TRALI [22–24], whereas bioactive lipids [25] and exosomes [11, 26] have emerged as candidate mediators that may amplify inflammatory injury in susceptible recipients. Exosomes are exogenous blood components newly recognized for their immunomodulatory effects [27, 28]; these nanoscale vesicles accumulate during RBC storage and vary in burden across units [29–31]. Exosome burden in stored blood products has therefore been proposed as a candidate donor-side metric in transfusion-related inflammatory injury [26], providing a rationale for rapid assessment of exosome burden in stored RBC units. Electrochemical aptamer-based (E-AB) sensors offer high specificity and rapid quantification [32], and CD63 aptamer–based E-ABs detect exosomes [33], although their applicability to blood products remains unproven. Accordingly, a CD63 aptamer–based electrochemical platform may provide a feasible analytical approach for rapid assessment of RBC-derived exosome (EXs-RBC) burden in stored RBC units.

Beyond assessing EXs-RBC burden, defining the biological effects of EXs-RBC is necessary to evaluate whether these vesicles represent functional mediators in TRALI-relevant inflammatory settings. EXs-RBC have been shown to activate mast cells and induce the release of multiple inflammatory mediators from these rapidly responsive immune cells [28]. Beyond their classical role in allergic reactions [34, 35], mast cells have been implicated in inflammatory lung injury [36–39] and may participate in TRALI-related immune responses [40]. Together, these prior observations raised the possibility that EXs-RBC may engage recipient mast cells as a TRALI-relevant inflammatory amplifier. Mechanistically, Toll-like receptor 3 (TLR3) and mitogen-activated protein kinase (MAPK) signaling are plausible pathways linking EXs-RBC exposure to mast-cell inflammatory activation and lung injury [28, 41, 42]. Hence, we hypothesized that EXs-RBC may contribute to mast-cell-associated inflammatory lung injury in TRALI-relevant settings through TLR3–MAPK signaling.

Therefore, in this study, we aimed to develop a CD63 aptamer–based electrochemical platform as a preliminary analytical approach for assessing EXs-RBC burden directly in stored RBC units, to investigate whether EXs-RBC are associated with mast-cell–related inflammatory lung injury in TRALI-relevant models, and to assess the involvement of TLR3–MAPK signaling. This study provides a proof-of-concept framework for rapid EXs-RBC burden assessment and for evaluating EXs-RBC as candidate donor-side mediators in TRALI-relevant inflammatory lung injury.

## Materials and methods

### Study design and ethical approval

All reagents, animals, cell lines, and culture conditions are described in the Experimental Section and the Additional file 1; an overview of the study workflow is provided in Additional file 1: Supplementary Figure S1. The human protocol (Approval No. K2023-03–035) was approved by the Fujian Provincial Hospital Ethics Committee in accordance with the Declaration of Helsinki, and written informed consent was obtained from all participants prior to enrollment. All animal procedures (Certificate No. FPH.PZ.20230506[0002]) were approved by the Experimental Animal Welfare Ethics Committee of Fujian Provincial Hospital and conducted in accordance with the NIH Guide for the Care and Use of Laboratory Animals. A comprehensive reagent list is provided in Additional file 1: Table S1, and detailed experimental protocols are available in the Supporting Methods. The CD63 aptamer (Apt-63) bearing a 5′ disulfide-protected thiol (C6 linker) was synthesized and HPLC-purified by Sangon Biotech (China); The thiol group was used for immobilization on the gold electrode surface, whereas MB served as the electrochemical indicator for SWV readout.

Apt-63 sequence: 5′-HS-(CH₂)₆-CACCCCACCTCGCTCCCGTGACACTAATGCTA-3′. Detailed procedures for CD63 E-AB fabrication are provided in the Additional file 1.

### CD63 E-AB sensor fabrication and EXs-RBC burden assessment

A methylene blue (MB)-labeled CD63 aptamer monolayer was self-assembled on gold and backfilled with MCH to form an electrochemical aptamer-based (E-AB) interface for EXs-RBC burden assessment. MB served as the electrochemical reporter, and exosome binding increased the average distance between the MB tag and the electrode surface, thereby slowing electron transfer and decreasing the square-wave voltammetry (SWV) current in a signal-off manner. For assays, Apt/MCH-modified electrodes were incubated with samples for 30 min at room temperature (RT), gently rinsed with phosphate-buffered saline (PBS), and signals were reported as ΔI/I₀ (CV/DPV) or ΔR_ct/R_ct,0 (EIS), where I₀ (or R_ct,0) denotes the post-MCH baseline.

To establish detectability and dose–response, EXs-RBC were spiked into PBS at three nanoparticle tracking analysis (NTA)-calibrated particle concentrations (3.0 × 10¹⁰, 9.0 × 10¹⁰, and 15.0 × 10¹⁰ particles/mL), which were selected on the basis of pilot experiments to provide a practically relevant range and clearly distinguishable electrochemical responses. These samples were then measured with the sensor. To assess concordance between direct sensor output and exosome particle concentration, RBC-unit samples were measured directly by the CD63 E-AB sensor, while EXs-RBC isolated from matched aliquots were quantified by NTA; the NTA-measured particle concentrations were then correlated with the corresponding sensor signals. To test whether the E-AB signal in RBC matrices was driven by EXs-RBC, pre-quantified EXs-RBC were spiked into PBS-diluted RBC samples at increasing particle concentrations (3.0 × 10¹⁰, 6.0 × 10¹⁰, and 12.0 × 10¹⁰ particles/mL), and changes in sensor response were measured. To assess EXs-RBC fraction-associated signal specificity, PBS, isolated EXs-RBC, and exosome-depleted RBC-unit supernatant were measured with the CD63 aptamer-based sensor under identical conditions. To assess aptamer-dependent specificity, responses of CD63 aptamer-modified electrodes were compared with those of irrelevant/non-CD63 aptamer-modified electrodes (Apt-12, Apt-32, and Apt-33) under identical EXs-RBC exposure conditions.

### Clinical samples, exosome preparation, and uptake assay

The isolation, characterization, and all related experiments of EXs were performed with reference to the ISEV MISEV2023 guidelines [43]. Following MISEV2023, we refer to these ultracentrifugation-enriched, exosome-like vesicle preparations as exosomes throughout this manuscript for consistency. Stored RBC units were maintained at 2–6 °C before supernatant collection. Exosomes were isolated by sequential differential centrifugation and ultracentrifugation from platelet-free plasma of healthy volunteers and from stored RBC-unit supernatants. Briefly, samples were centrifuged at 450 × g for 10 min to remove residual cells, 2,000 × g for 10 min to remove cell debris and large particles, and 10,000 × g for 10 min to deplete apoptotic bodies and large vesicles, followed by PBS washing and ultracentrifugation at 100,000 × g for 90 min to collect the EXs-enriched pellet and reduce soluble co-isolates. The final EXs pellet was resuspended in PBS for downstream characterization and functional experiments. A schematic overview of the EXs isolation and quality-control workflow is provided in Fig. 2A, and detailed procedures are provided in the Supporting Methods.

**Fig. 1:**
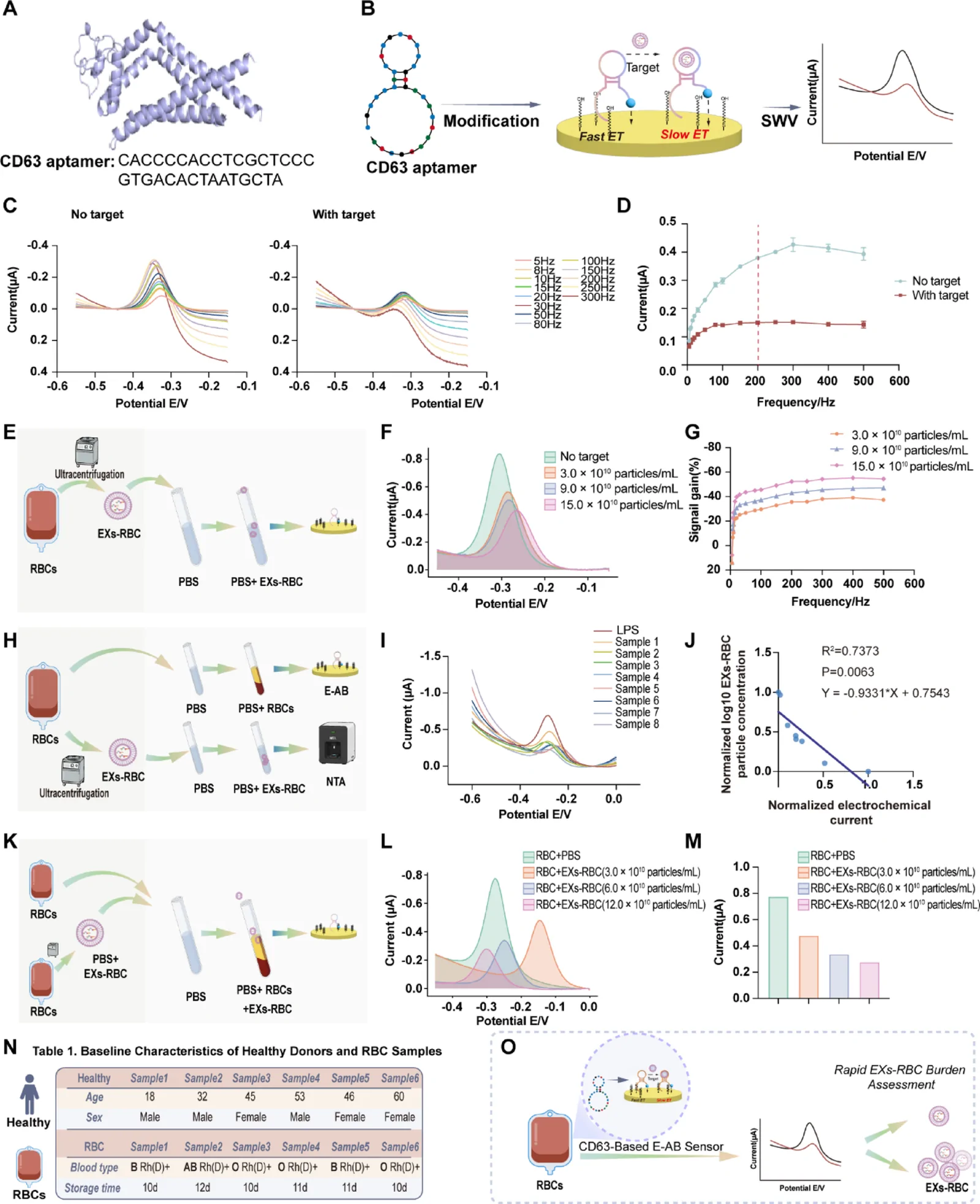
A CD63 aptamer–based electrochemical sensor exhibits an inverse, concentration‑dependent response to EXs‑RBC. (**A**) Three-dimensional structural model of the CD63 aptamer and its nucleotide sequence. (**B**) Principle of the electrochemical aptamer‑based (E‑AB) sensor: A redox‑labeled CD63 aptamer is immobilized on the electrode; Target binding increases the aptamer–electrode distance, slows electron transfer, and decreases the square‑wave voltammetry (SWV) peak current (Ip). (**C**) Representative SWV traces across frequencies. (**D**) Frequency scan: Ip rises with frequency in the absence of target but is suppressed and plateaus when target is present. (**E**) Workflow for detecting RBC-derived exosomes using a CD63 aptamer–based electrochemical sensor. (**F**) SWV current–potential curves for different NTA-calibrated EXs-RBC particle concentrations in PBS. (**G**) Ip decreased stepwise with higher exosome levels. (**H**) Workflow for direct EXs-RBC burden assessment in RBC units by the CD63 aptamer–based electrochemical sensor and matched NTA particle quantification of isolated EXs-RBC. (**I**) SWV current–potential curves from RBC-unit samples with different NTA-calibrated EXs-RBC particle concentrations. (**J**) Linear regression analysis between normalized E-AB current signals measured directly in RBC units and NTA-measured particle concentrations from matched isolated EXs-RBC preparations. (**K**) Schematic: Purified EXs‑RBC were added to the same RBC suspension and measured with a CD63 aptamer–based SWV sensor. (**L**) Representative SWV traces from the same unit showing stepwise decreases in Ip with increasing exosome spike‑in. (**M**) Summary of Ip for the same sample, confirming an inverse, dose‑dependent response. (**N**) Baseline characteristics of donors and RBC units used in this study. (**O**) Overview: The CD63 aptamer–based sensor supports rapid, isolation-free assessment of relative EXs-RBC burden directly in RBC units

Transmission electron microscopy (TEM) was used to assess vesicle morphology. NTA was performed to evaluate particle size distribution and particle concentration. Particle concentrations were determined by NTA after 1:1000 dilution and were back-calculated to the original EXs stock. Based on NTA calibration, the working EXs stock contained approximately 3 × 10^10^ particles/mL, corresponding to 1 µg/µL total exosome protein and approximately 3 × 10^7^ particles/µg protein. Therefore, HMC-1 cells were treated with 1.5 × 10^9^ particles/mL, and mice received 1.5 × 10^9^ particles/mouse. The corresponding protein amounts were 50 µg/mL and 50 µg, respectively [43–45]. Exosome preparations were further characterized by western blotting for exosome-associated markers, including CD63, CD9, CD81, TSG101, and ALIX, with calnexin assessed as a cellular contamination marker.

For correlation analysis, stored RBC-unit samples were measured directly using the CD63 E-AB sensor, while EXs-RBC isolated from matched aliquots were quantified by NTA. NTA-measured particle concentrations were log10-transformed and normalized, and then analyzed against normalized electrochemical current signals by linear regression. For uptake studies, DiI (1,1′-dioctadecyl-3,3,3′,3′-tetramethylindocarbocyanine perchlorate) –labeled EXs-RBC or EXs-nor were incubated with cells, followed by PBS washes and fluorescence microscopy to visualize internalization. These assays confirmed cellular uptake relevant to downstream mast-cell activation experiments. For endosomal localization analysis, DiI-labeled EXs-RBC were incubated with HMC-1 cells. Cells were then stained for Rab7 as an endosomal marker and counterstained with DAPI. Confocal microscopy was used to assess the colocalization of EXs-RBC with Rab7-positive endosomes.

### Cell culture, treatments, and functional assays

HMC-1 human mast cells (Shanghai Cell Bank, Shanghai, China) were maintained in complete RPMI-1640 at 37℃ and 5% CO_2_. Cells were treated with vehicle (PBS), lipopolysaccharide (LPS), or exosomes as indicated. LPS was applied at 1 µg/mL in accordance with published protocols [46, 47] and our pilot experiments. Exosome preparations were adjusted to either 1 µg/µL or 2 µg/µL total protein, depending on the experimental design, and were calibrated by NTA. The 1 µg/µL stock yielded an estimated particle concentration of approximately 3 × 10^10^ particles/mL, while the 2 µg/µL concentrated stock yielded approximately 6 × 10^10^ particles/mL and was used for volume-matched add-back experiments.

To evaluate exosome effects in HMC-1 cells, cultures were exposed to PBS, EXs-nor, or EXs-RBC at 1.5 × 10^9^ particles/mL. To interrogate mast-cell degranulation, TLR3 activation, and MAPK signaling, cells were pre-treated for 1 h with the indicated inhibitors: cromolyn sodium (50 µM, mast-cell stabilizer); CU-CPT-4a (5 µM, TLR3 antagonist); CC-401 (5 µM, JNK inhibitor); SCH772984 (5 µM, ERK1/2 inhibitor); and TAK-715 (5 µM, p38 MAPK inhibitor), followed by exposure to EXs-RBC. Inflammatory responses were assessed by quantitative polymerase chain reaction (qPCR), enzyme-linked immunosorbent assay (ELISA), and western blotting. To assess whether soluble non-vesicular components in RBC-unit supernatants contributed to the observed responses, exosome-depleted supernatant and EXs-RBC add-back control experiments were performed in HMC-1 cells. The post-ultracentrifugation supernatant was collected as exosome-depleted RBC-unit supernatant (EX-depleted Sup), and EXs-RBC were added back at the same particle-equivalent dose used in the EXs-RBC group. Volume-matched 50 µL treatment mixtures containing PBS, EXs-RBC, EX-depleted Sup, or EX-depleted Sup plus EXs-RBC were applied to HMC-1 cells. Detailed mixture compositions are provided in the Supplementary Methods.

For RNase pretreatment experiments, EXs-RBC were incubated with RNase A before addition to HMC-1 cells to assess whether RNA-sensitive vesicular cargo contributed to TLR3-MAPK activation. After RNase pretreatment, EXs-RBC were applied to HMC-1 cells at the same particle-equivalent dose as untreated EXs-RBC.

### Animals and experimental procedures

Male BALB/c mice were used for the antibody-mediated TRALI-like model, LPS-induced ARDS model, sepsis model, and EXs-RBC administration experiments. Male mast cell–deficient C57BL/6-Kit^W-sh/W-sh^ mice and their wild-type littermates were used specifically for experiments assessing the role of mast-cell deficiency. For each experiment, mice were randomized into groups of six using R software; an additional 10% of animals were pre-randomized and reserved to replace periprocedural mortality per protocol. If a mouse died before the planned endpoint, it was replaced by the next animal from the reserve cohort.

To determine whether EXs-RBC impair lung function and whether this effect depends on mast cells or the TLR3/MAPK pathway, BALB/c mice received intratracheal instillation of EXs-RBC (50 µL; approximately 1.5 × 10^9^ particles/mouse, corresponding to 50 µg total exosome protein). In dose-escalation experiments, a higher dose was administered (50 µL; approximately 3 × 10^9^ particles/mouse, corresponding to 100 µg total exosome protein). Selected groups were pretreated 1 h before intratracheal instillation with cromolyn (50 mg/kg), CU-CPT-4a (TLR3 antagonist, 10 mg/kg), CC-401 (JNK inhibitor, 10 mg/kg), TAK-715 (p38 inhibitor, 10 mg/kg), or SCH772984 (ERK1/2 inhibitor, 10 mg/kg), as described previously [40]. To interrogate the role of mast cells in EXs-RBC–induced lung injury, mast cell–deficient (MC^⁻/⁻^) and wild-type C57BL/6 mice received intratracheal EXs-RBC (50 µL; 1.5 × 10^9^ particles/mouse) [48, 49]. Mice were euthanized 24 h after intratracheal instillation by intraperitoneal overdose of sodium pentobarbital (100 mg/kg), and samples were collected for analysis. The intratracheal dosing regimen was adapted from a published protocol [50–53] and further validated in our pilot dose-finding experiments to elicit a reproducible, non-lethal pulmonary response suitable for mechanistic studies.

To determine whether the observed biological effects were mediated by EXs-RBC, mice received intratracheal administration of volume-matched 50 µL preparations containing PBS, EXs-RBC, EX-depleted Sup, or EX-depleted Sup plus EXs-RBC. EXs-RBC was added back at the same particle-equivalent dose used in the EXs-RBC group (approximately 1.5 × 10⁹ particles/mouse). Mice were euthanized 24 h later, and lung tissues and BALF were collected for analysis.

To assess whether exosomes alone induced lung inflammatory responses in healthy mice, male BALB/c mice received a single tail-vein injection of PBS, EXs-nor, or EXs-RBC (1.5 × 10⁹ particles/mouse in 50 µL PBS for exosome groups). Mice were euthanized 24 h after injection, and lung tissues and BALF were collected for analysis.

To evaluate the donor-side effects of EXs-RBC under recipient inflammatory conditions, two antibody-independent TRALI-relevant lung injury models were used. Specifically, male BALB/c mice were challenged with LPS to induce either pulmonary inflammatory/ARDS-like lung injury (intratracheal, 50 µL of 1 µg/µL LPS) or systemic inflammatory/sepsis-like lung injury (intraperitoneal, 50 µL of 3 µg/µL LPS). One hour later, mice received a single tail-vein bolus of PBS or exosomes (EXs-nor or EXs-RBC; 1.5 × 10⁹ particles/mouse in 50 µL PBS for exosome groups; dose selection based on our pilot data and prior reports [54]). Control groups received the corresponding volume-matched PBS injections. The experimental groups included Sham, PBS + PBS, LPS alone, LPS + EXs-nor, and LPS + EXs-RBC. No anti–MHC-I antibody was administered in these two models. Animals were euthanized 24 h after intravenous administration, and samples were collected for analysis.

To assess whether EXs-RBC could further exacerbate antibody-mediated TRALI-like lung injury, mice were primed with LPS (0.1 mg/kg, i.p.; *Escherichia coli*, L8880; Solarbio) for 24 h and then challenged with an anti–MHC-I monoclonal antibody (1 mg/kg in 100 µL PBS) via tail-vein injection. One hour later, PBS, EXs-nor, or EXs-RBC was administered as a single tail-vein bolus (1.5 × 10⁹ particles/mouse in 50 µL PBS for exosome groups). This antibody-mediated TRALI-like model was established as previously described and validated in preliminary experiments [6, 40]. Mice subjected to this model were euthanized 2 h after the anti–MHC-I challenge, and samples (lungs and bronchoalveolar lavage fluid) were collected for subsequent analyses.

### Unilateral BALF collection, lung wet-to-dry ratio, and histological scoring

To evaluate pulmonary inflammation and alveolar–capillary barrier permeability under the indicated conditions, bronchoalveolar lavage fluid (BALF) was collected after euthanasia by lavaging the left lung three times with 0.4 mL of ice-cold phosphate-buffered saline (PBS). To restrict lavage to the left lung and preserve the right lung for subsequent analyses, the right main bronchus was ligated before lavage. The recovered BALF (approximately 1 mL per mouse) was collected into centrifuge tubes and centrifuged at 500 × g for 5 min to remove cells. The supernatant was harvested for subsequent protein quantification or further analyses.

The lung wet-to-dry weight ratio was determined using the right cranial lung lobe. Briefly, the right cranial lobe was excised immediately after euthanasia, gently blotted to remove surface fluid, and weighed to obtain the wet weight. Samples were then dried at 60 °C until a constant weight was reached and weighed again to obtain the dry weight. The wet-to-dry ratio was calculated as wet weight divided by dry weight.

Histological lung injury was quantified in a blinded manner using an established acute lung injury scoring system as previously described [55]. Lung injury was assessed using a semi-quantitative scoring system based on four parameters: alveolar congestion, hemorrhage, inflammatory cell infiltration in the alveolar spaces or vessel walls, and alveolar wall thickening/hyaline membrane formation. Each parameter was scored from 0 to 4 according to the affected area: 0, absent; 1, < 25%; 2, 25–50%; 3, 50–75%; and 4, > 75%. The total lung injury score for each H&E-stained field was calculated as the sum of the four parameters, with a maximum score of 16. Six H&E-stained fields from each group were evaluated in a blinded manner and used for histological score analysis.

### Molecular and histological assays

mRNA levels in cells and lung tissues were quantified by qPCR and normalized to 18 S RNA; primer sequences are listed in Table S2. Protein expression of TPSAB1, TLR3, and MAPK (total and phosphorylated JNK/p38/ERK) was assessed by western blotting; band intensities were quantified in ImageJ (NIH). β-Hexosaminidase A (β-Hex A) release in HMC-1 culture supernatants was measured by ELISA as a functional degranulation-associated readout. In this study, mast-cell activation was assessed using inflammatory mediator expression, TLR3/MAPK signaling, and β-Hex A release, with β-Hex A release interpreted as a degranulation-associated readout. Myeloperoxidase (MPO) in lung homogenates was measured by ELISA. BALF was collected, and total protein concentrations were determined as an index of alveolar–capillary barrier permeability. Lung edema was assessed by calculating the W/D weight ratio. Histopathology was evaluated on hematoxylin and eosin (H&E)-stained lung sections.

### Statistical analysis

Data are presented as mean ± SEM unless stated otherwise. Normality was assessed with the Shapiro–Wilk test. Two-tailed tests were used: Student’s t-test for two groups and one-way ANOVA with Dunnett’s post hoc test for multiple comparisons. Analyses were performed in R (v4.4.2). Statistical significance was set at *P* < 0.05.

## Results

### A CD63 aptamer–based electrochemical sensor exhibits an inverse, concentration‑dependent response to EXs‑RBC

The three-dimensional structure and nucleotide sequence of the CD63 aptamer are shown in Fig. 1A. As illustrated in Fig. 1B, exosome binding to aptamer increases the aptamer–electrode distance, slows electron transfer, and reduces the square-wave voltammetry (SWV) peak current (**I**_**p**_). Across scans from 5 to 300 Hz, **I**_**p**_ was higher without target than with EXs-RBC. As the frequency increased, the currents of both groups rose rapidly at lower frequencies but their slopes became markedly smaller around 200 Hz, indicating that the responses were approaching a plateau. Although a somewhat larger current difference was still observed at 300 Hz, further increasing the frequency beyond 200 Hz did not produce a substantial additional increase in response. Therefore, 200 Hz was selected as the working frequency for subsequent measurements (Fig. 1C, D). In specificity-control experiments, electrodes modified with irrelevant/non-CD63 aptamers (Apt-12, Apt-32, and Apt-33) showed markedly lower current changes in response to EXs-RBC than CD63 aptamer-modified electrodes (Supplementary Figure S2A–D), supporting a CD63 aptamer-dependent E-AB response. In addition, exosome-depleted RBC-unit supernatant produced a current response comparable to PBS, whereas isolated EXs-RBC markedly reduced the current signal (Supplementary Figure S2E, F), supporting that the E-AB response was mainly associated with the EXs-RBC fraction.

**Fig. 2:**
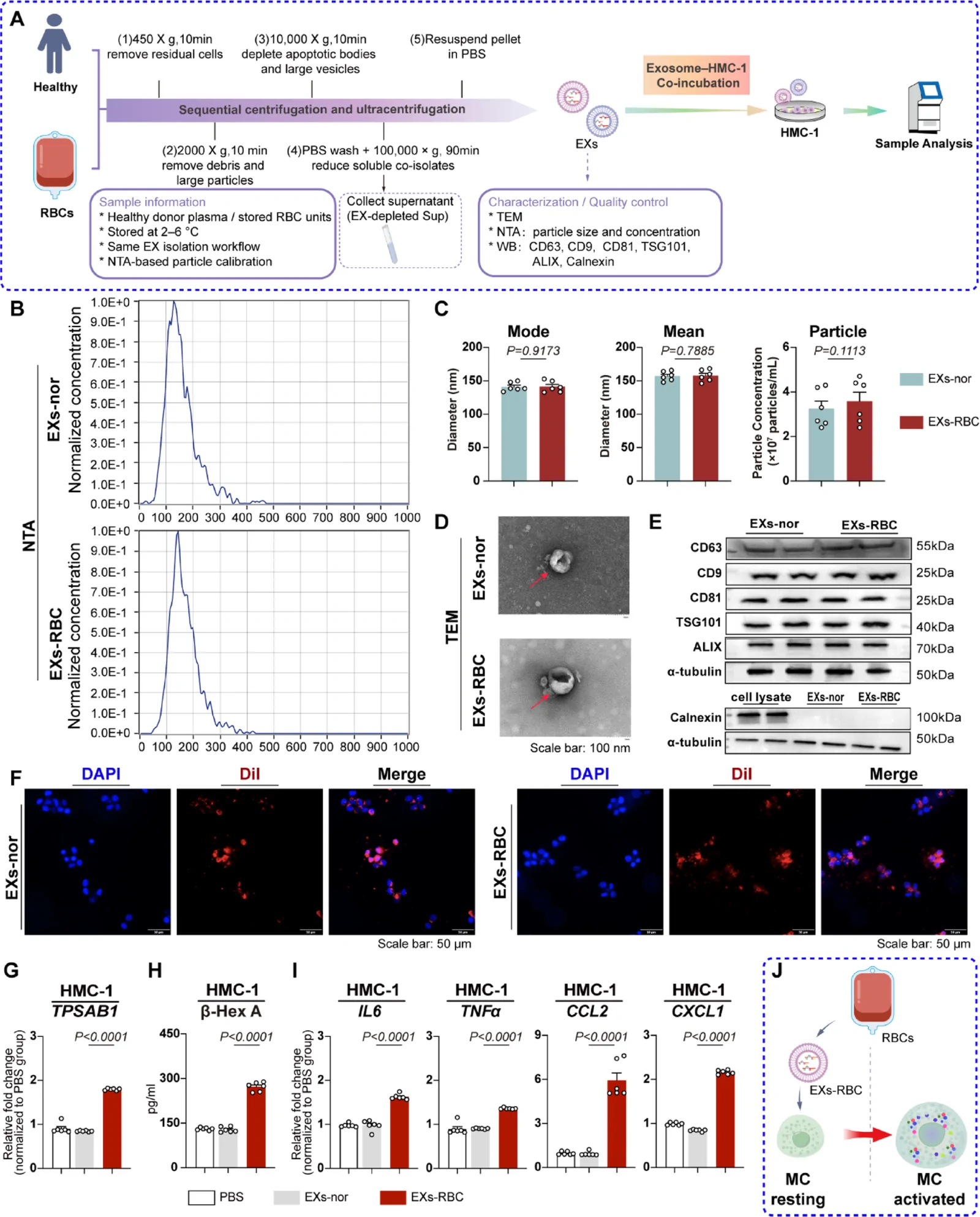
Isolation, characterization, uptake, and inflammatory effects of EXs-RBC in HMC-1 mast cells. (**A**) Schematic overview of EXs isolation, exosome-depleted supernatant collection, quality-control characterization, and HMC-1 co-incubation workflow. (**B**) Representative NTA size-distribution profiles of EXs-nor and EXs-RBC. (**C**) Quantification of mode diameter, mean diameter, and NTA-measured particle concentration after 1:1000 dilution; stock particle concentrations were back-calculated from the diluted measurements. (**D**) Representative TEM images of EXs-nor and EXs-RBC showing exosome-like vesicle morphology. Scale bar, 100 nm. (**E**) Western blot characterization of exosome-associated markers CD63, CD9, CD81, TSG101, and ALIX, with calnexin assessed as a cellular contamination marker. (**F**) Uptake of DiI-labeled EXs-nor or EXs-RBC by HMC-1 cells counterstained with DAPI. Scale bar, 50 μm. (**G**) qPCR analysis of TPSAB1 expression in HMC-1 cells. (H) ELISA results for β-Hexosaminidase A. (**I**) qPCR analysis of IL6, TNFα, CCL2, and CXCL1 expression in HMC-1 cells. (**J**) Schematic summary showing EXs-RBC internalization and HMC-1 mast-cell activation. Data are presented as mean ± SEM; statistical significance was determined by one-way ANOVA followed by post hoc multiple-comparison testing

In PBS (Fig. 1E), increasing NTA-calibrated EXs-RBC particle concentrations (3.0 × 10¹⁰, 9.0 × 10¹⁰, and 15.0 × 10¹⁰ particles/mL) produced stepwise reductions in **I**_**p**_ and progressively more negative signal change values (Fig. 1F, G), supporting that the electrochemical sensor discriminates EXs-RBC concentrations in PBS.

In RBC-unit samples (Fig. 1H), eight RBC units with different storage durations and NTA-calibrated EXs-RBC particle concentrations were analyzed. The blood types and storage durations of these units are summarized in Supplementary Figure S2G. Higher EXs-RBC particle burden was associated with lower Ip values (Fig. 1I). Linear regression analysis showed a significant inverse correlation between normalized E-AB current signals measured directly in RBC units and NTA-measured particle concentrations from the corresponding isolated EXs-RBC preparations (R² = 0.7373, *P* = 0.0063; Fig. 1J), supporting the sensor’s ability to discriminate EXs-RBC particle burden in RBC units.

In spike‑in experiments (Fig. 1K), representative traces showed stepwise decreases in **I**_**p**_ with increasing EXs‑RBC levels (Fig. 1L), and summary analysis confirmed a dose-dependent inverse response (Fig. 1M), indicating that signal suppression was driven by exosome binding. Baseline characteristics of donors and RBCs used in this study are shown in Fig. 1N. These data support the analytical feasibility of a CD63 aptamer-based sensor for rapid, concentration-dependent assessment of relative EXs-RBC burden directly in RBC units without prior isolation, supporting its potential as a candidate pretransfusion quality-control readout (Fig. 1O).

### EXs‑RBC are characterized, internalized by HMC‑1 mast cells, and induce pro‑inflammatory gene expression

As illustrated in Fig. 2A, exosomes were isolated from healthy donor plasma and stored RBC units using a sequential centrifugation/ultracentrifugation workflow with downstream quality-control characterization. NTA showed comparable size-distribution profiles for EXs-nor and EXs-RBC (Fig. 2B). Quantification of NTA parameters showed similar mode diameter, mean diameter, and diluted particle concentrations between EXs-nor and EXs-RBC (Fig. 2C). TEM revealed cup-shaped, membrane-enclosed small vesicles in both preparations (Fig. 2D). Both EXs‑nor and EXs‑RBC were enriched for exosomal markers CD63, CD9, CD81, TSG101, and ALIX, with minimal calnexin signal, by western blotting (Fig. 2E). Fluorescence microscopy confirmed internalization of DiI-labeled exosomes by HMC-1 cells (Fig. 2F). Compared with EXs‑nor, EXs‑RBC significantly increased tryptase alpha/beta 1 (*TPSAB1*) mRNA in HMC‑1 cells (*P* < 0.05; Fig. 2G), β-Hex A release (Fig. 2H), and upregulated interleukin-6 (IL6), tumor necrosis factor-alpha (TNFα), chemokine (C-C motif) ligand 2 (CCL2), and chemokine (C-X-C motif) ligand 1 (CXCL1) mRNA (Fig. 2I). These findings suggest that EXs-RBC induce mast-cell activation, thereby promoting inflammatory mediator release **(**Fig. 2J).

### EXs-RBC add-back restores inflammatory responses after exosome depletion

**Fig. 3:**
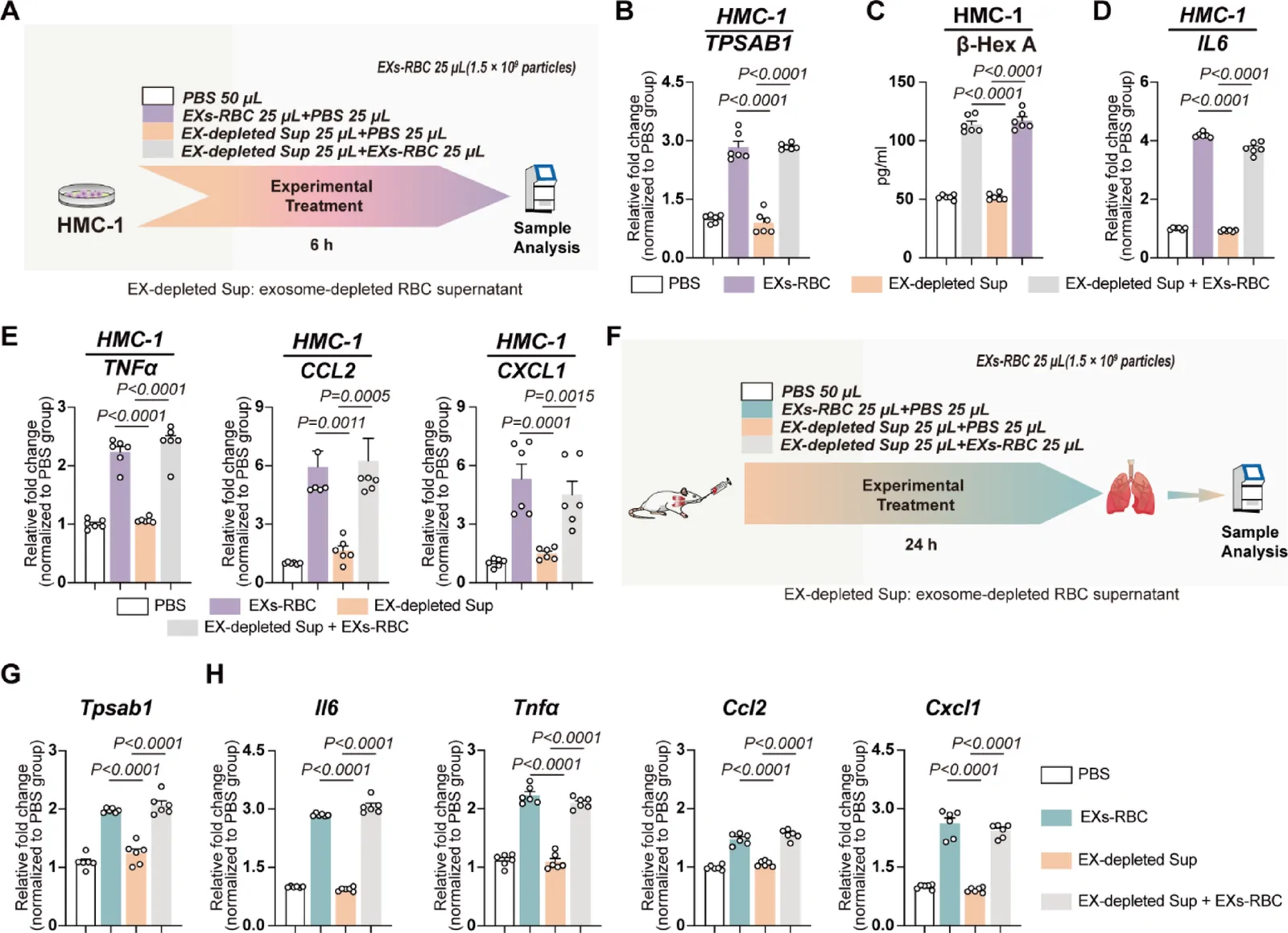
EXs-RBC add-back restores inflammatory responses after exosome depletion in HMC-1 cells and mice. (**A**) Experimental workflow for HMC-1 cell treatment with PBS, EXs-RBC, exosome-depleted RBC-unit supernatant (EX-depleted Sup), or EX-depleted Sup plus EXs-RBC. (B) qPCR analysis of TPSAB1 expression in HMC-1 cells. (**C**) ELISA results for β-Hexosaminidase in HMC-1 cells. (**D**) qPCR analysis of IL6 expression in HMC-1 cells. (**E**) qPCR analysis of TNFα, CCL2, and CXCL1 expression in HMC-1 cells. (**F**) Experimental workflow for mouse intratracheal administration of PBS, EXs-RBC, EX-depleted Sup, or EX-depleted Sup plus EXs-RBC. (**G**) qPCR analysis of pulmonary Tpsab1 expression. (**H**) qPCR analysis of pulmonary Il6, Tnfα, Ccl2, and Cxcl1 expression. Data are presented as mean ± SEM. Statistical significance was determined by one-way ANOVA followed by post hoc multiple-comparison testing

We next tested whether the inflammatory responses were attenuated after exosome depletion and restored by EXs-RBC add-back. The HMC-1 experimental workflow is shown in Fig. 3A. In HMC-1 cells, EXs-RBC markedly increased TPSAB1 expression compared with PBS, whereas EX-depleted Sup induced only a weak response (Fig. 3B). Similar patterns were observed for β-Hex A release (Fig. 3C) and IL6, TNFα, CCL2, and CXCL1 expression (Fig. 3D, E). Re-addition of EXs-RBC to EX-depleted Sup restored these responses to levels comparable to those induced by EXs-RBC alone.

The mouse experimental workflow is shown in Fig. 3F. EX-depleted Sup induced weaker pulmonary Tpsab1 and inflammatory mediator expression than EXs-RBC, whereas EX-depleted Sup plus EXs-RBC restored these responses (Fig. 3G, H). Together, these results support that the inflammatory activity of stored RBC-unit supernatants was mainly associated with the EXs-RBC-enriched fraction.

### Intratracheal administration of EXs-RBC activates mast cells and aggravates lung inflammatory injury in a dose-dependent manner

**Fig. 4:**
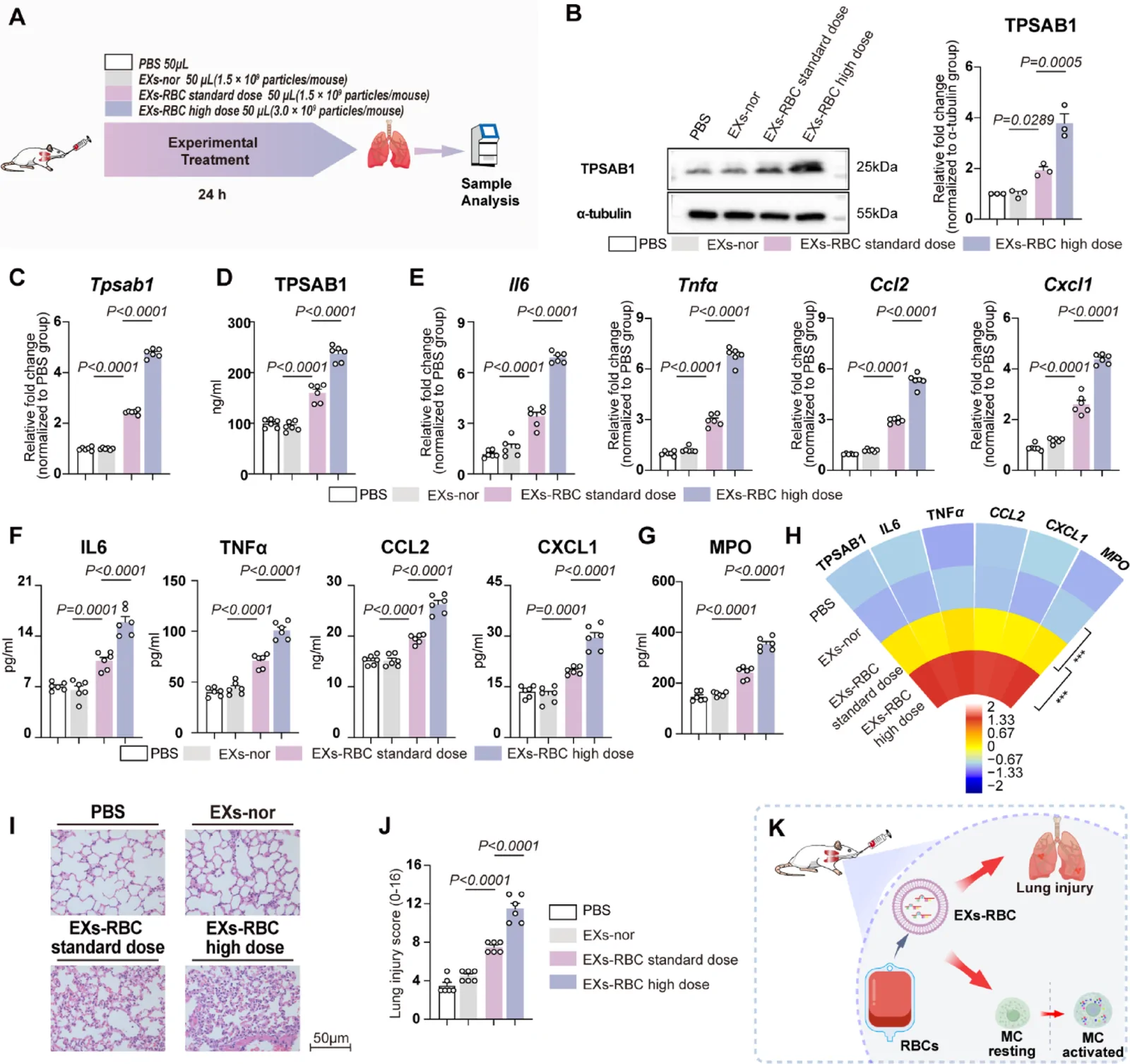
EXs-RBC activate mast cells and aggravate lung inflammation and injury in a dose-dependent manner. **(A)** Schematic of intratracheal administration of exosomes and subsequent lung analysis. **(B)** Representative western blots of TPSAB1 (left) and densitometric quantification by ImageJ (right). **(C)** qPCR results for *TPSAB1* mRNA. **(D)** ELISA results for TPSAB1. **(E)** qPCR results for *IL6*,* TNFα*,* CCL2*, and *CXCL1* mRNA. **(F)** ELISA results for IL6, TNFα, CCL2, and CXCL1. **(G)** ELISA results for MPO. **(H)** Heatmap of row-wise Z-score–normalized ELISA concentrations (pg/mL or ng/mL) from three independent experiments. **(I)** Representative H&E-stained lung sections. **(J)** Quantitative histological lung injury scores. **(K)** Schematic: EXs-RBC induce mast-cell activation and lung injury in mice. Standard dose, 1.5 × 10⁹ particles/mouse; high dose, 3.0 × 10⁹ particles/mouse. Data are mean ± SEM from three independent experiments with technical duplicates; statistical significance was assessed by one‑way ANOVA (*, *P* < 0.05; ***, *P* < 0.001)

The experimental workflow is shown in Fig. 4A. Immunoblotting showed elevated TPSAB1 protein in EXs-RBC groups compared with PBS and EXs-nor, with dose dependence confirmed by densitometry (Fig. 4B). Consistent increases were observed at the mRNA and protein levels by qPCR and ELISA (Fig. 4C, D). EXs-RBC increased inflammatory cytokines including IL6, TNFα, CCL2, and CXCL1 in a dose-dependent manner (qPCR, Fig. 4E; ELISA, Fig. 4F). MPO levels were also elevated in EXs-RBC groups (Fig. 4G), and heatmap analysis showed coordinated increases in mast-cell and inflammatory injury markers (Fig. 4H). Histology revealed progressive lung injury after EXs-RBC challenge, and quantitative lung injury scoring further confirmed dose-dependent histological damage (Fig. 4I, J). These results indicate that EXs-RBC induce mast-cell activation and amplify lung inflammatory injury (Fig. 4K).

### Cromolyn pre-administration or mast-cell deficiency markedly diminishes EXs-RBC-induced mast-cell activation and associated lung inflammatory injury

**Fig. 5:**
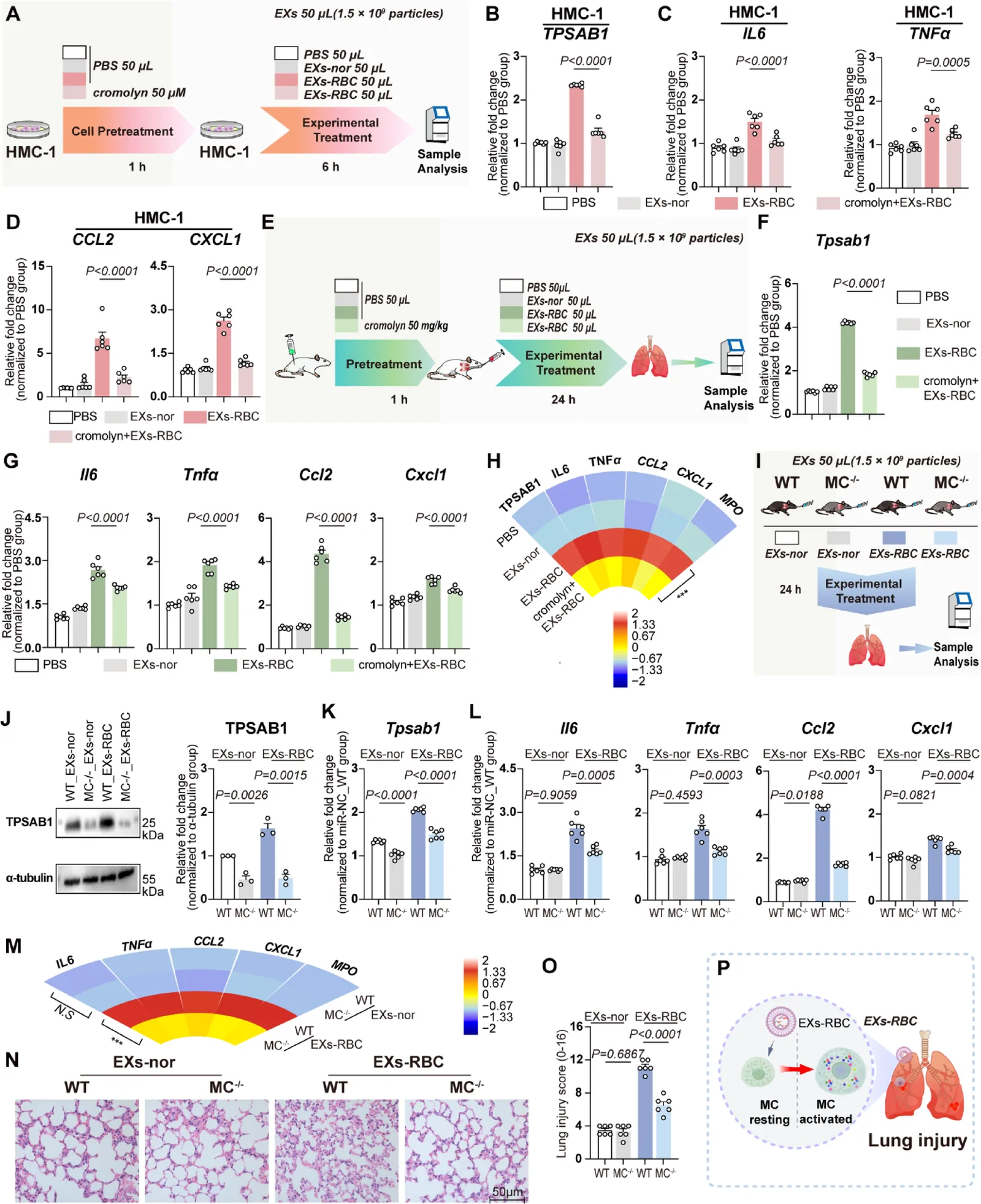
Cromolyn pretreatment or mast-cell deficiency diminishes EXs-RBC-induced mast-cell activation and associated lung injury. **(A)** Experimental design for EXs-RBC-challenged HMC-1 cells with or without pre-administration of cromolyn. Data are expressed as mean ± SEM from three independent experiments; statistical significance was determined by one-way ANOVA followed by Dunnett’s multiple comparisons test versus EXs-RBC-challenged group. **(B)** qPCR results for *TPSAB1* mRNA. **(C**,** D)** qPCR results for *IL6*,* TNFα*,* CCL2*, and *CXCL1* mRNA. **(E)** Experimental design for EXs-RBC-challenged mice with or without pre-administration of cromolyn. **(F)** qPCR results for *Tpsab1* mRNA. **(G)** qPCR results for *Il6*,* Tnfα*,* Ccl2*, and *Cxcl1* mRNA. **(H)** Heatmap of row-wise Z-score–normalized ELISA concentrations averaged from three independent experiments. **(I)** Experimental design for EXs-RBC-challenged mast cell-deficient C57BL/6-Kit^W−sh/W−sh^ mice. **(J)** Representative western blots of TPSAB1 (left) and densitometric quantification by ImageJ (right). **(K)** qPCR results for *Tpsab1* mRNA. **(L)** qPCR results for *Il6*,* Tnfα*,* Ccl2*, and *Cxcl1* mRNA. **(M)** Heatmap of row-wise Z-score–normalized ELISA-measured concentrations for IL6, TNFα, CCL2, CXCL1, and MPO. **(N)** Representative H&E-stained lung sections. **(O)** Quantitative histological lung injury scores. **(P)** Schematic summary showing the contribution of mast-cell activation to EXs-RBC-associated lung inflammatory injury. Data are expressed as mean ± SEM from three independent experiments; each group has six mice (*n* = 6); statistical significance was determined by one-way ANOVA followed by Dunnett’s multiple comparisons test. ns, no statistical significance; ***, *P* < 0.001

Following the procedure depicted in Fig. 5A, pretreatment with cromolyn effectively inhibited EXs-RBC–induced mast cell activation, as evidenced by a significant reduction in *TPSAB1* expression (Fig. 5B). Cromolyn also decreased the mRNA levels of inflammatory markers *IL6*,* TNFα*,* CCL2*, and *CXCL1* in EXs-RBC–treated HMC-1 cells (Fig. 5C, D). In mice, following the procedure depicted in Fig. 5E, cromolyn pretreatment significantly reduced pulmonary *Tpsab1*,* Il6*,* Tnfα*,* Ccl2*, and *Cxcl1* expression after EXs-RBC challenge (Fig. 5F, G), and decreased TPSAB1, IL6, TNFα, CCL2, CXCL1, and MPO protein levels (Fig. 5H; Supplementary Figure S3A–C). Following the procedure depicted in Fig. 5I, mast cell-deficient C57BL/6-Kit^W−sh/W−sh^ mice showed reduced TPSAB1 protein and Tpsab1 mRNA compared with wild-type mice after EXs-RBC challenge (Fig. 5J, K). After EXs-RBC intratracheal injection, these mast cell–deficient mice showed significantly reduced tryptase (TPSAB1) protein (western blots) in Fig. 5J and *Tpsab1* mRNA in Fig. 5K, as well as lower levels of inflammatory markers in the lungs, including IL6, TNFα, CCL2, and CXCL1, as shown by qPCR (Fig. 5L) and ELISA (Fig. 5M; Supplementary Figure S3D). MPO levels (ELISA; Fig. 5M; Supplementary Figure S3E) and histological analysis (Fig. 5N) further demonstrated less lung injury in these mice compared with wild‑type controls. Quantitative lung injury scoring further confirmed reduced histological damage in mast-cell-deficient mice after EXs-RBC challenge (Fig. 5O). These results support a contributory role of mast-cell activation in EXs-RBC–associated lung injury (Fig. 5P).

### EXs-RBC upregulates TLR3 and activates MAPK signaling in vitro and in vivo

**Fig. 6:**
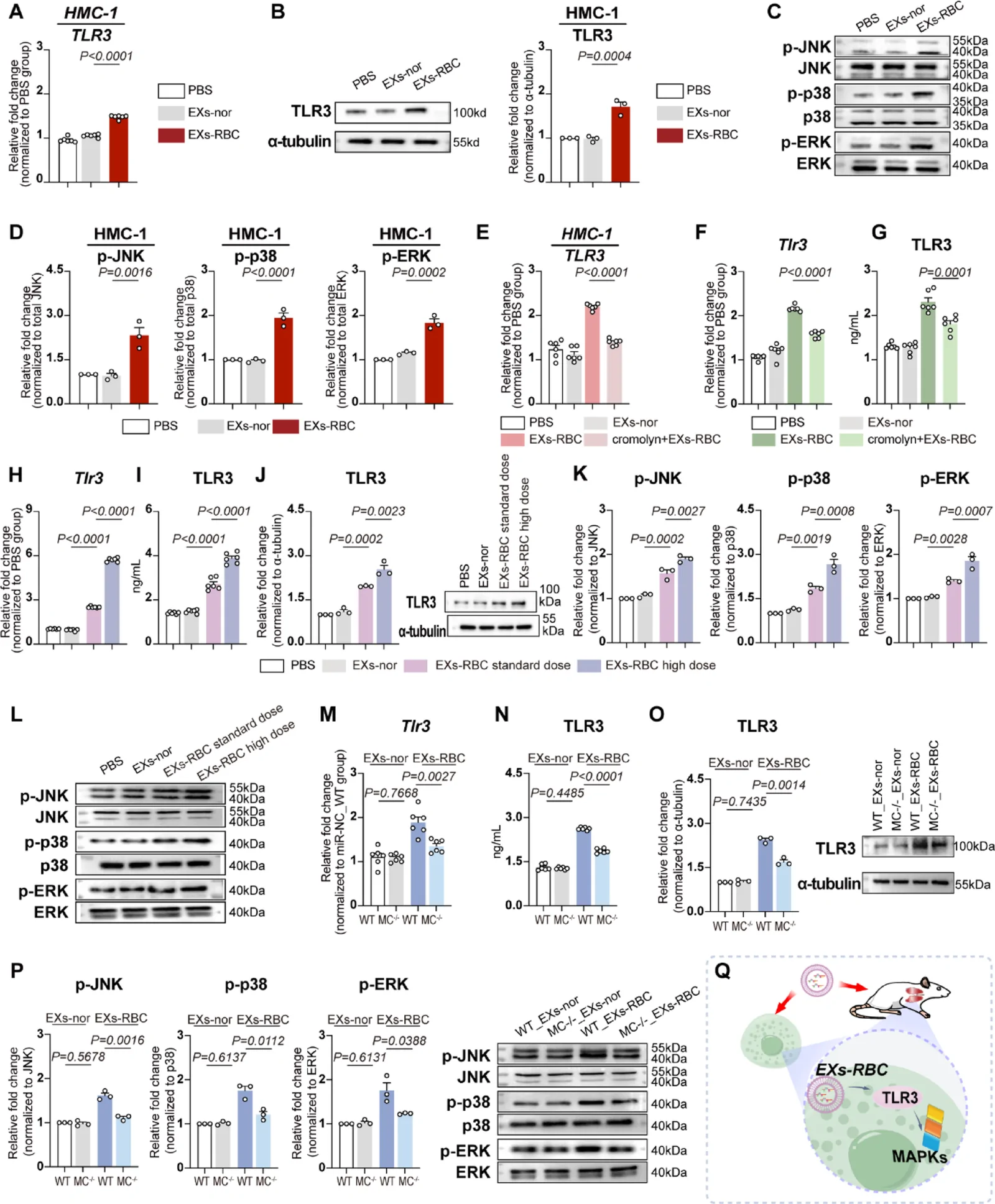
EXs-RBC upregulates TLR3 and activates MAPK signaling in vitro and in vivo. **(A–D)** HMC-1 mast cells treated with EXs-RBC showed increased TLR3 expression and activation of MAPK signaling. **(A)** qPCR results for TLR3. **(B)** Western blots of TLR3; representative blots (left) and densitometry (right), quantified by ImageJ. **(C)** Representative western blots showing increased phospho-JNK, phospho-p38, and phospho-ERK1/2 with total JNK, p38, and ERK1/2 controls. **(D)** Densitometry of phospho-to-total ratios (p-JNK/JNK, p-p38/p38, p-ERK1/2/ERK1/2), quantified by ImageJ. **(E)** qPCR results for TLR3 in EXs‑RBC–treated HMC‑1 cells with or without cromolyn pretreatment. **(F–G)** Mice treated with EXs-RBC with or without cromolyn pretreatment: **(F)** qPCR results for *Tlr3*; **(G)** ELISA results for TLR3 protein. **(H–L)** Lung results from mice treated with increasing doses of EXs-RBC (dose-dependent design). Standard dose, 1.5 × 10⁹ particles/mouse; high dose, 3.0 × 10⁹ particles/mouse. **(H)** qPCR results for *Tlr3*. **(I)** ELISA results for TLR3 protein. **(J)** TLR3 western blots: Densitometry (left) and representative blots (right), quantified by ImageJ. **(K)** Densitometry of phospho‑to‑total ratios (p‑JNK/JNK, p‑p38/p38, p‑ERK1/2/ERK1/2). **(L)** Representative western blots showing dose‑dependent increases in phospho‑JNK, phospho‑p38, and phospho‑ERK1/2 with total JNK, p38, and ERK1/2 controls. **(M–N)** Expression of *Tlr3* (qPCR) and TLR3 protein (ELISA) in the lungs of exosome-treated, mast cell–deficient mice. **(O)** TLR3 western blots: Densitometry (left) and representative blots (right), quantified by ImageJ. **(P)** Representative western blots comparing wild-type and mast cell–deficient mice, showing reduced phospho-JNK, phospho-p38, and phospho-ERK1/2 with total JNK, p38, and ERK1/2 controls. Data are presented as mean ± SEM from three independent experiments (in vitro) and *n* = 6 per group (in vivo); statistical significance was determined by one-way ANOVA with Dunnett’s multiple-comparisons test versus control. **(Q)** Proposed model: EXs-RBC upregulates TLR3 and activates MAPK pathways in HMC-1 mast cells and in mouse lungs

TLR3 levels were elevated in EXs-RBC–treated HMC-1 mast cells, as confirmed by qPCR (Fig. 6A) and western blot (Fig. 6B). Phosphorylation of JNK, p38, and ERK1/2 was increased in EXs-RBC–treated HMC-1 cells, while total JNK, p38, and ERK1/2 levels remained unchanged (Fig. 6C, D). Cromolyn pretreatment attenuated the EXs-RBC–induced increase in TLR3 expression in HMC-1 mast cells (Fig. 6E). In the lungs of EXs-RBC–treated mice, pre-administration of cromolyn significantly reduced *Tlr3* expression (qPCR, Fig. 6F) and TLR3 protein (ELISA, Fig. 6G).

EXs-RBC increased TLR3 in the lungs of mice in a dose-dependent manner (qPCR, Fig. 6H; ELISA, Fig. 6I; and western blots, Fig. 6J). Phosphorylation of JNK, p38, and ERK1/2 was dose-dependently increased in EXs-RBC–treated mice, while total JNK, p38, and ERK1/2 levels were unchanged (Fig. 6K, L). Compared with wild‑type controls, mast cell–deficient mice showed an attenuation of the EXs‑RBC–induced increase in lung TLR3 expression (qPCR (Fig. 6M), ELISA (Fig. 6N), and western blotting (Fig. 6O)). Phosphorylation of JNK, p38, and ERK1/2 was increased in EXs‑RBC–treated wild‑type mice, whereas it was attenuated in mast cell–deficient mice, and total JNK, p38, and ERK1/2 levels remained unchanged (Fig. 6P). Collectively, these data indicate that EXs-RBC upregulates TLR3 and activates MAPK pathways in mast cells and in mouse lungs (Fig. 6Q).

Because TLR3 is localized in endosomal compartments, we further assessed whether internalized EXs-RBC could access late endosomes in HMC-1 cells. DiI-labeled EXs-RBC partially overlapped with Rab7-positive compartments, supporting late endosomal access of internalized EXs-RBC (Supplementary Figure S4). These findings provide spatial support for the association between EXs-RBC uptake and endosomal TLR3-associated signaling.

### Pharmacologic blockade of TLR3 or MAPK dampened EXs-RBC–driven mast-cell activation and lung inflammation

**Fig. 7:**
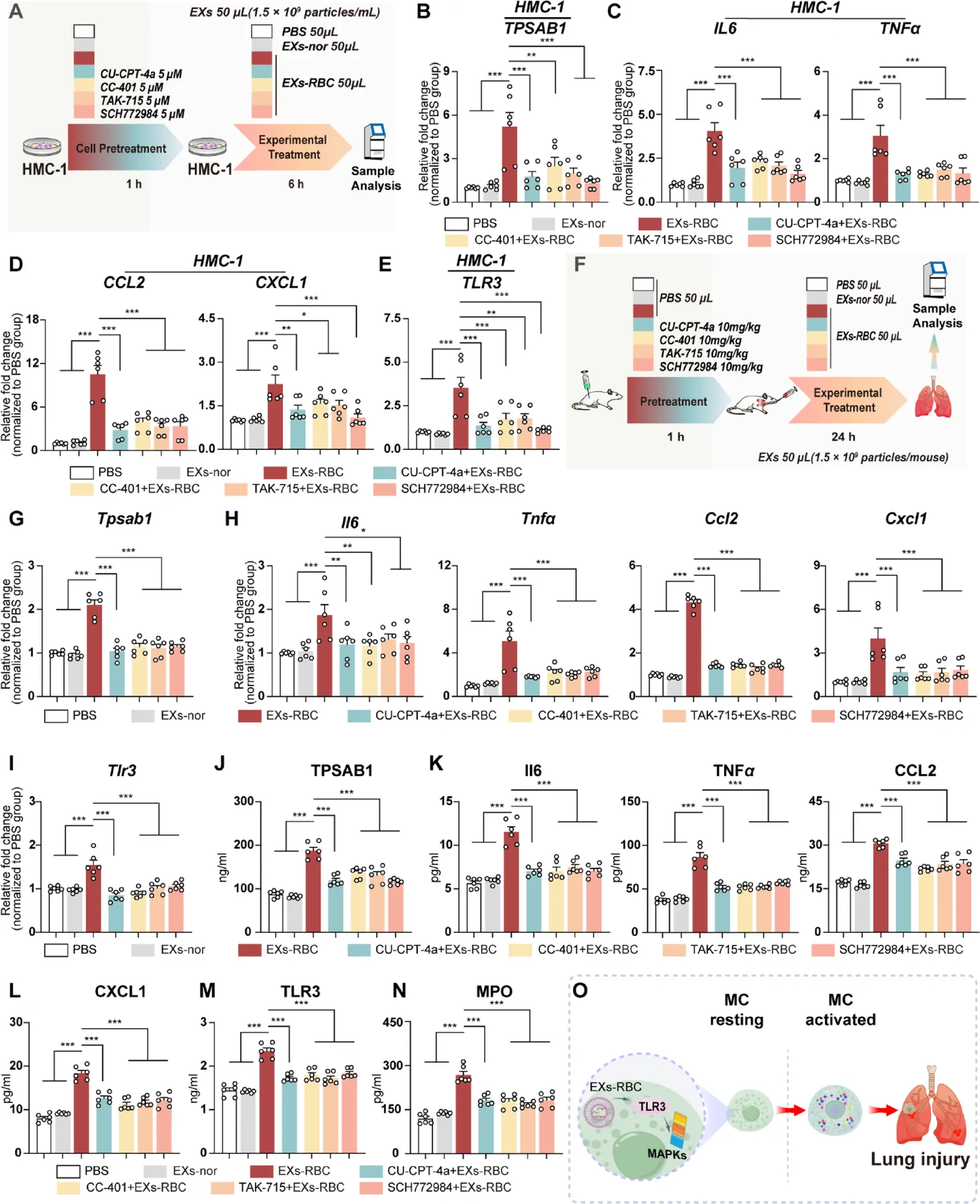
Blocking TLR3 or MAPK suppresses EXs-RBC–induced mast-cell activation and lung inflammatory injury. **(A)** Experimental design for EXs-RBC challenge in HMC-1 mast cells, with or without pretreatment with a TLR3 antagonist (CU-CPT-4a) or MAPK pathway inhibitors (CC-401 [JNK], TAK-715 [p38], SCH772984 [ERK1/2]). Data are presented as mean ± SEM from three independent experiments; statistical significance was determined by one-way ANOVA followed by Dunnett’s multiple-comparisons test. **(B)** qPCR results for *TPSAB1* mRNA. **(C–D)** qPCR results for *IL6*,* TNFα*,* CCL2*, and *CXCL1* mRNA. **(E)** qPCR results for TLR3 mRNA. **(F)** Experimental design for mice intratracheally injected with EXs-RBC, with or without pre-administration of TLR3 antagonist (CU-CPT-4a) or MAPK inhibitors. Data are presented as mean ± SEM; *n* = 6 per group, and statistical significance was determined by one-way ANOVA with Dunnett’s multiple-comparisons test. **(G)** qPCR results for *Tpsab1* mRNA. **(H)** qPCR results for *Il6*,* Tnfα*,* Ccl2*, and *Cxcl1* mRNA. **(I)** qPCR results for *Tlr3* mRNA. **(J)** ELISA results for TPSAB1. **(K–L)** ELISA results for IL6, TNFα, CCL2, and CXCL1. **(M)** ELISA results for TLR3. **(N)** ELISA results for MPO. **(O)** Proposed model: TLR3 and MAPK signaling mediate EXs-RBC–induced mast cell activation and pulmonary injury. ***, *P* < 0.001

Following the paradigm in Fig. 7A, pretreatment with CU‑CPT‑4a (TLR3 inhibitor), CC-401 (JNK inhibitor), SCH772984 (ERK1/2 inhibitor), and TAK-715 (p38 inhibitor) reduced EXs-RBC–induced mast-cell activation, as indicated by decreased *TPSAB1* expression (Fig. 7B). These inhibitors also attenuated EXs-RBC–induced increases in IL6, TNFα, CCL2, and CXCL1 transcripts in HMC-1 cells (Fig. 7C, D) and decreased *TLR3* mRNA (Fig. 7E). Following Fig. 7F, pre-administration of the same inhibitors significantly reduced pulmonary *Il6*,* Tnfα*,* Ccl2*,* Cxcl1*, and *Tlr3* mRNA, in mice intratracheally injected with EXs-RBC (qPCR; Fig. 7G–I**)** and lowered TPSAB1, IL6, TNFα, CCL2, CXCL1, and TLR3 protein levels (ELISA; Fig. 7J–M). Furthermore, inhibitor treatment reduced MPO (Fig. 7N). Collectively, pharmacologic blockade of TLR3 or MAPK dampened EXs-RBC–driven mast-cell activation and lung inflammation (Fig. 7O).

RNase pretreatment attenuated EXs-RBC-induced TPSAB1 expression, inflammatory mediator expression, TLR3 expression, and MAPK phosphorylation in HMC-1 cells, suggesting that RNA-sensitive cargo contributes, at least in part, to EXs-RBC-mediated mast-cell activation through TLR3-MAPK signaling (Supplementary Figure S5).

### EXs-RBC exacerbate lung inflammation and injury in an antibody-independent LPS-induced ARDS-like model

Before testing antibody-independent inflammatory priming models, we first examined whether intravenous EXs-RBC alone induced lung inflammatory responses in healthy mice. Compared with PBS or EXs-nor, EXs-RBC alone did not significantly alter pulmonary Tpsab1, Il6, Tnfα, Ccl2, Cxcl1, or Tlr3 mRNA expression in healthy mice (Supplementary Figure S6). These data suggest that EXs-RBC alone had limited inflammatory activity in healthy lungs under the tested conditions.

**Fig. 8:**
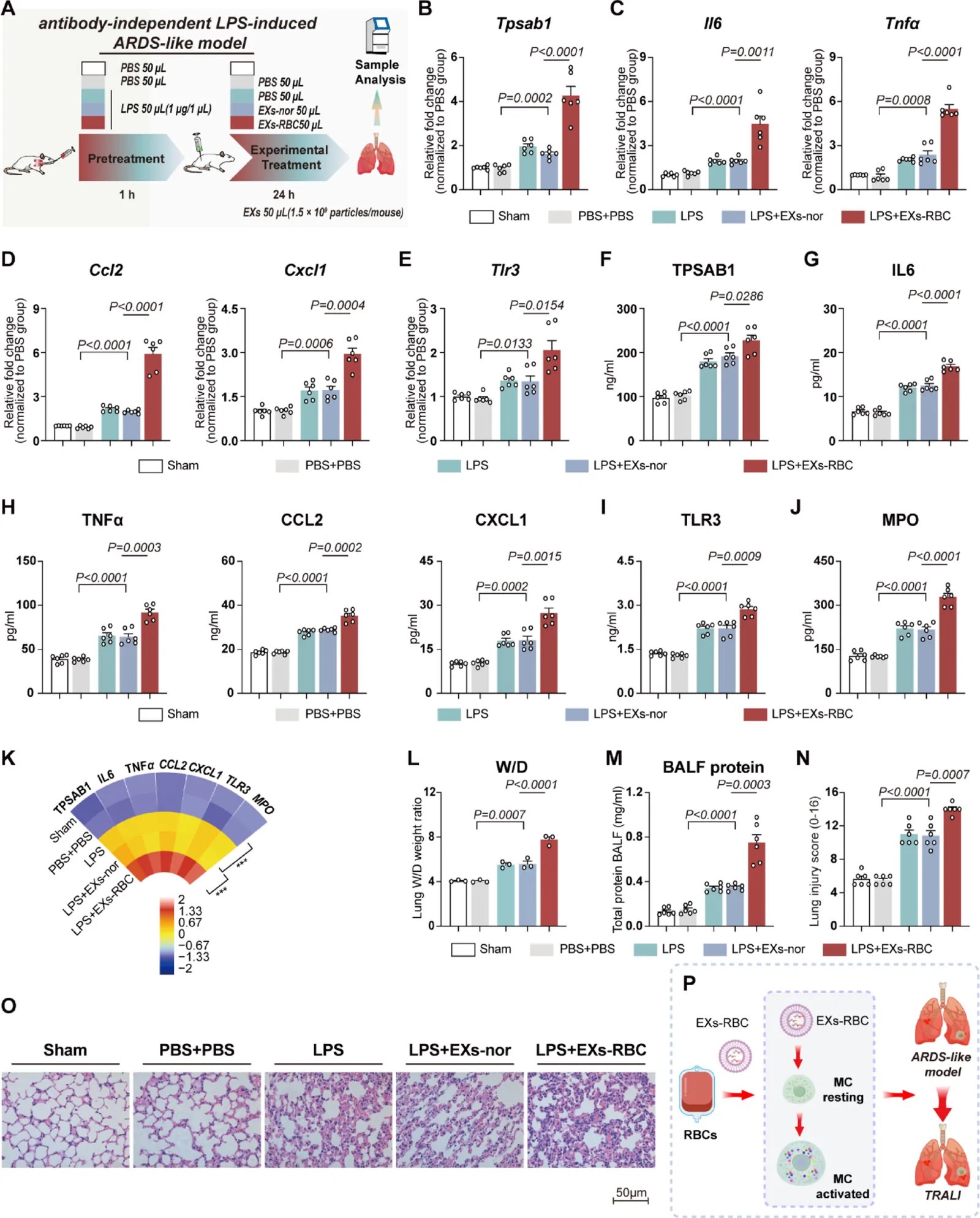
EXs-RBC exacerbate lung inflammation and injury in an antibody-independent LPS-induced ARDS-like model. **(A)** Experimental design for intratracheal LPS-induced ARDS-like lung injury followed by intravenous administration of PBS, EXs-nor, or EXs-RBC. (**B**) qPCR analysis of pulmonary Tpsab1 expression. (**C**) qPCR analysis of pulmonary Il6 and Tnfα expression. (**D**) qPCR analysis of pulmonary Ccl2 and Cxcl1 expression. (**E**) qPCR analysis of pulmonary Tlr3 expression. (**F**) ELISA analysis of TPSAB1. (**G**) ELISA analysis of IL6. (**H**) ELISA analysis of TNFα, CCL2, and CXCL1. (I) ELISA analysis of TLR3. (**J**) ELISA analysis of MPO. (**K**) Heatmap of row-wise Z-score-normalized inflammatory and injury-associated readouts. (**L**) Lung wet-to-dry weight ratio. (**M**) BALF total protein concentration. (**N**) Quantitative histological lung injury score. (**O**) Representative H&E-stained lung sections. (**P**) Schematic summary of EXs-RBC-associated exacerbation of lung inflammation in the ARDS-like model. Data are presented as mean ± SEM; *n* = 6 mice per group; statistical significance was determined by one-way ANOVA followed by post hoc multiple-comparison testing; exact P values are shown in the panels. Scale bar, 50 μm

To evaluate the donor-side effect of EXs-RBC under antibody-independent pulmonary inflammatory priming, BALB/c mice were challenged intratracheally with LPS and then received PBS, EXs-nor, or EXs-RBC by intravenous injection (Fig. 8A). LPS challenge increased pulmonary Tpsab1, inflammatory mediator, and Tlr3 expression, confirming induction of an ARDS-like inflammatory lung injury model (Fig. 8B–E). Compared with LPS plus EXs-nor, LPS plus EXs-RBC further increased TPSAB1, IL6, TNFα, CCL2, CXCL1, TLR3, and MPO levels (Fig. 8F–J). EXs-RBC also increased the lung W/D ratio, BALF total protein concentration, and histological lung injury score, with more severe inflammatory cell infiltration and alveolar structural injury on H&E staining (Fig. 8K–O). These results indicate that EXs-RBC exacerbate lung inflammation and injury in an antibody-independent LPS-induced ARDS-like model.

### EXs-RBC exacerbate lung inflammation and injury in an antibody-independent LPS-induced sepsis-like model

**Fig. 9:**
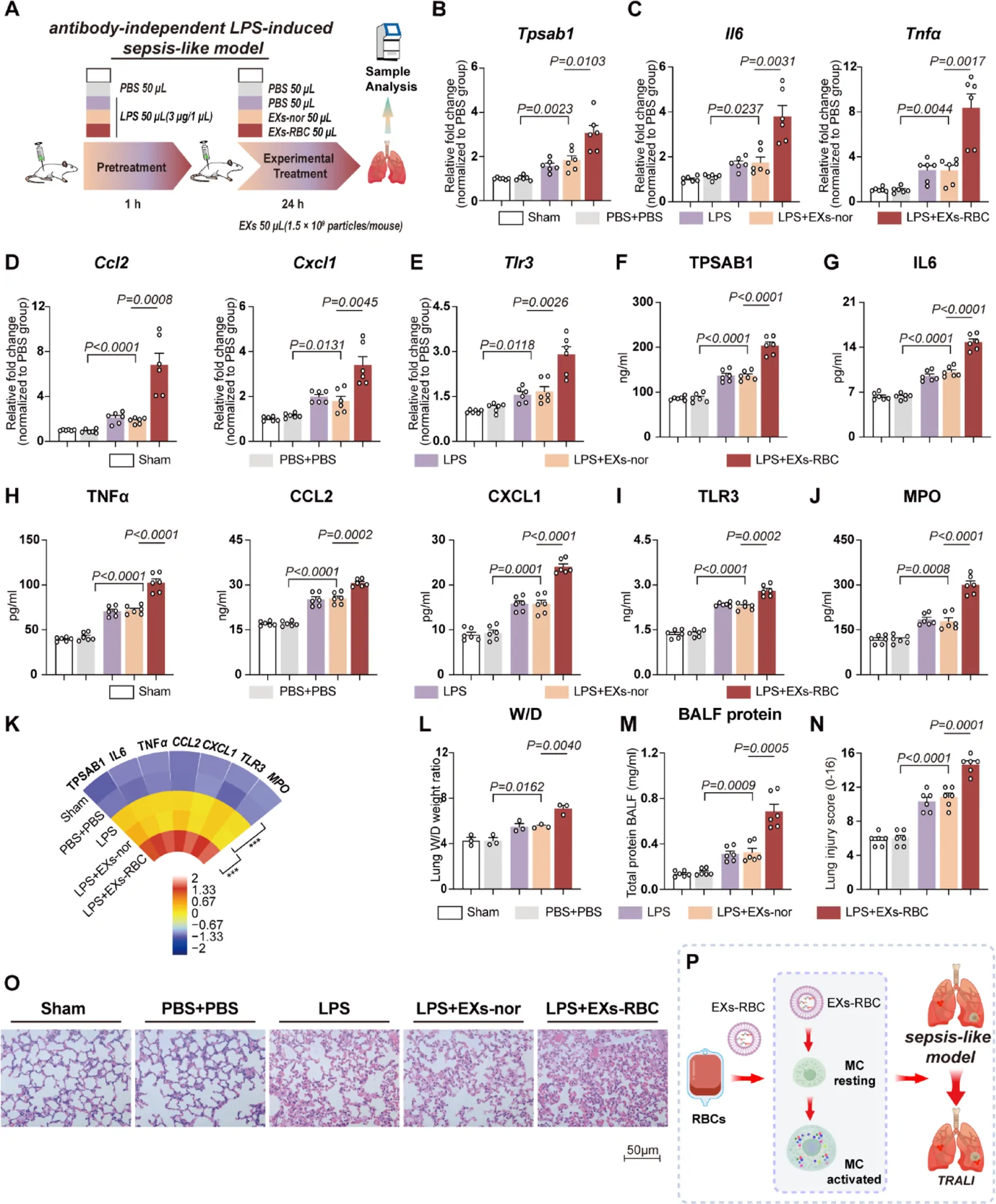
EXs-RBC exacerbate lung inflammation and injury in an antibody-independent LPS-induced sepsis-like model. (**A**) Experimental design for intraperitoneal LPS-induced sepsis-like lung injury followed by intravenous administration of PBS, EXs-nor, or EXs-RBC. (**B**) qPCR analysis of pulmonary Tpsab1 expression. (**C**) qPCR analysis of pulmonary Il6 and Tnfα expression. (**D**) qPCR analysis of pulmonary Ccl2 and Cxcl1 expression. (**E**) qPCR analysis of pulmonary Tlr3 expression. (**F**) ELISA analysis of TPSAB1. (**G**) ELISA analysis of IL6. (**H**) ELISA analysis of TNFα, CCL2, and CXCL1. (**I**) ELISA analysis of TLR3. (**J**) ELISA analysis of MPO. (**K**) Heatmap of row-wise Z-score-normalized inflammatory and injury-associated readouts. (**L**) Lung wet-to-dry weight ratio. (**M**) BALF total protein concentration. (**N**) Quantitative histological lung injury score. (**O**) Representative H&E-stained lung sections. (**P**) Schematic summary of EXs-RBC-associated exacerbation of lung inflammation in the sepsis-like model. Data are presented as mean ± SEM; *n* = 6 mice per group; statistical significance was determined by one-way ANOVA followed by post hoc multiple-comparison testing; exact P values are shown in the panels. Scale bar, 50 μm

We next examined whether EXs-RBC exerted similar effects under systemic inflammatory priming. BALB/c mice were challenged intraperitoneally with LPS and then received PBS, EXs-nor, or EXs-RBC by intravenous injection (Fig. 9A). LPS challenge increased pulmonary Tpsab1, inflammatory mediator, and Tlr3 expression, confirming induction of a sepsis-like inflammatory lung injury model (Fig. 9B–E). Compared with LPS plus EXs-nor, LPS plus EXs-RBC further increased TPSAB1, IL6, TNFα, CCL2, CXCL1, TLR3, and MPO levels (Fig. 9F–J). EXs-RBC also increased the lung W/D ratio, BALF total protein concentration, and histological lung injury score, with more pronounced lung histopathological injury on H&E staining (Fig. 9K–O). These findings indicate that EXs-RBC also exacerbate lung inflammation and injury in an antibody-independent LPS-induced sepsis-like model.

### EXs-RBC further exacerbate lung inflammation in an anti–MHC-I antibody-mediated TRALI-like model

**Fig. 10:**
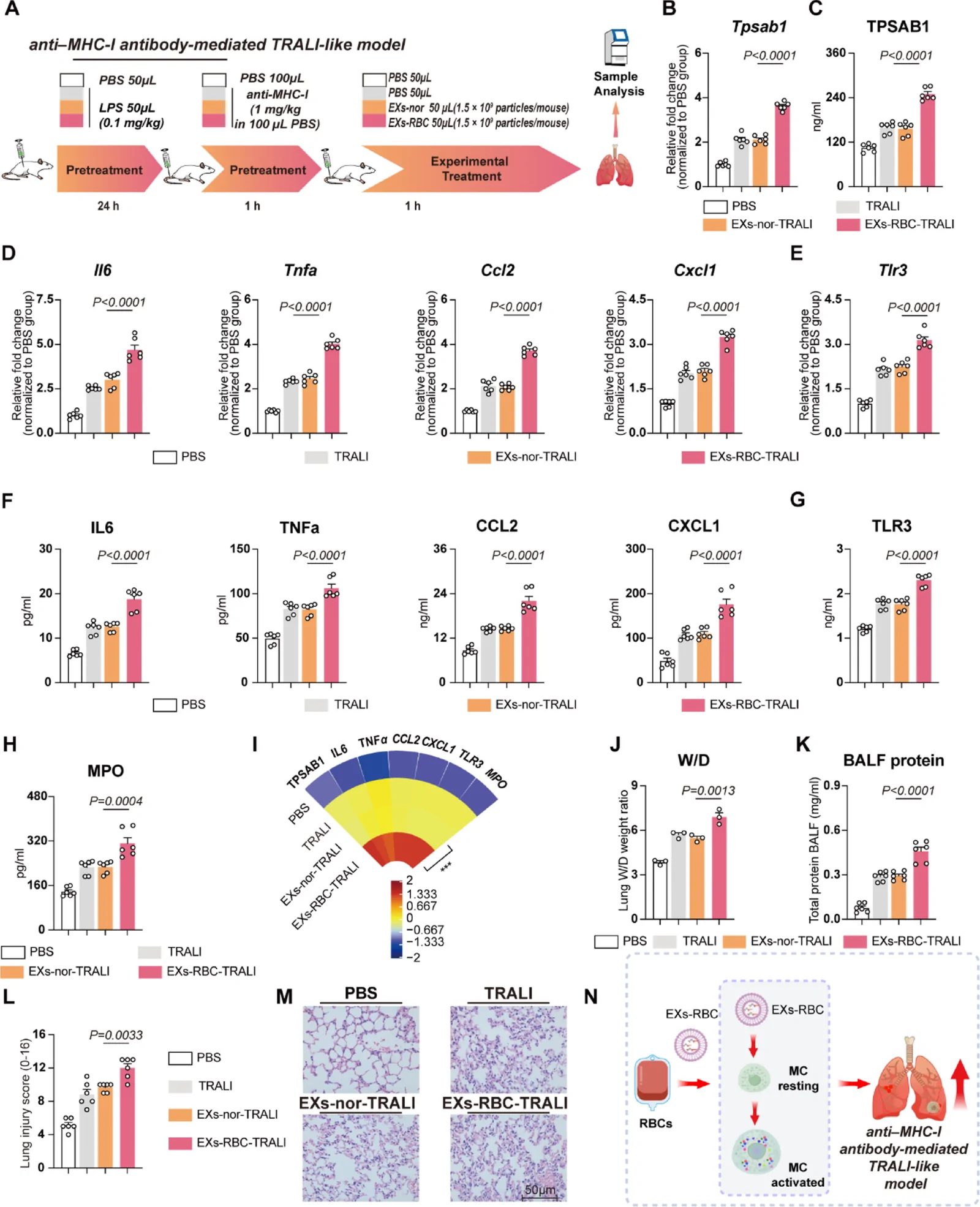
EXs-RBC further exacerbate lung inflammation in an anti-MHC-I antibody-mediated TRALI-like model. **(A)** Experimental scheme for the anti-MHC-I antibody-mediated TRALI-like model followed by administration of PBS, EXs-nor, or EXs-RBC. (**B**) qPCR analysis of pulmonary Tpsab1 expression. (**C**) ELISA analysis of TPSAB1. (**D**) qPCR analysis of pulmonary Il6, Tnfα, Ccl2, and Cxcl1 expression. (E) qPCR analysis of pulmonary Tlr3 expression. (**F**) ELISA analysis of IL6, TNFα, CCL2, and CXCL1. (**G**) ELISA analysis of TLR3. (**H**) ELISA analysis of MPO. (**I**) Heatmap of row-wise Z-score-normalized inflammatory and injury-associated readouts. (**J**) Lung wet-to-dry weight ratio. (**K**) BALF total protein concentration. (**L**) Quantitative histological lung injury score. (**M**) Representative H&E-stained lung sections. (**N**) Schematic summary showing EXs-RBC-associated exacerbation of lung inflammation under anti-MHC-I antibody-mediated TRALI-like conditions. Data are presented as mean ± SEM; *n* = 6 mice per group; statistical significance was determined by one-way ANOVA followed by post hoc multiple-comparison testing; exact P values are shown in the panels. Scale bar, 50 μm

Following the experimental paradigm shown in Fig. 10A, mice were primed with LPS and then challenged with anti-MHC-I antibody to establish an antibody-mediated TRALI-like model before administration of PBS, EXs-nor, or EXs-RBC. Compared with TRALI and EXs-nor-TRALI controls, EXs-RBC-TRALI further increased pulmonary Tpsab1 mRNA expression and TPSAB1 protein levels (Fig. 10B, C). EXs-RBC-TRALI also showed higher pulmonary Il6, Tnfα, Ccl2, Cxcl1, and Tlr3 mRNA expression (Fig. 10D, E), accompanied by increased IL6, TNFα, CCL2, CXCL1, and TLR3 protein levels (Fig. 10F, G, I). Consistently, EXs-RBC-TRALI further aggravated lung injury, as indicated by increased MPO levels, higher lung W/D ratios, elevated BALF total protein concentrations, increased histological lung injury scores, and more severe pathological changes on H&E staining (Fig. 10H–M). Together, these data indicate that EXs-RBC can further amplify mast-cell-associated inflammation and lung injury in an established anti-MHC-I antibody-mediated TRALI-like background (Fig. 10N).

## Discussion

This study supports the feasibility of using a CD63 E-AB sensor to rapidly assess relative EXs-RBC burden directly in stored RBC units and shows that EXs-RBC can amplify mast-cell-associated inflammatory lung injury in TRALI-relevant models. These findings support further evaluation of EXs-RBC burden as a candidate donor-side screening readout and provide experimental insight into mast-cell-associated inflammatory pathways relevant to TRALI. The CD63 E-AB sensor provides an inverse, concentration-dependent signal and reliably discriminates EXs-RBC burdens across units. We show that EXs‑RBC activate HMC‑1 mast cells and induce cytokine production. EXs‑RBC exacerbate lung inflammation and injury in a dose‑dependent manner, while mast‑cell inhibition or genetic deficiency attenuates these effects, supporting a contributory role of mast‑cell activation in EXs‑RBC–induced lung injury. Pharmacologic blockade of TLR3 or MAPK mitigates mast-cell activation and lung injury, supporting their involvement. Intravenous administration of EXs-RBC exacerbated pulmonary inflammation and injury in antibody-independent LPS-induced ARDS-like and sepsis-like models, with supportive evidence from an anti–MHC-I antibody-mediated TRALI-like model, supporting a contributory role of EXs-RBC in TRALI-relevant lung inflammation.

Analytically, our data support the CD63 aptamer–based sensor as a rapid, isolation‑free pretransfusion tool for assessing EXs-RBC burden. NTA is widely used to characterize exosomes by measuring particle size distribution and particle concentration but does not provide marker-specific information [56]. Nano-flow cytometry enables marker-based single-particle analysis [57]. However, both approaches generally require prior exosome enrichment or isolation and sample preparation. The CD63 aptamer-based E-AB sensor may provide a rapid, label-free electrochemical readout for assessing relative exosome burden with minimal sample processing, but it does not provide detailed size distribution, absolute particle concentration, or multiparameter vesicle phenotyping. Exosomes in blood products are implicated in TRALI and other transfusion‑related complications [6, 58], yet routine quantification requires prior vesicle isolation for protein or particle assays—a labor‑intensive, time‑consuming workflow that limits clinical implementation [59]. In our models, EXs-RBC dose-dependently exacerbated lung injury, indicating a need for pretransfusion assessment of EXs-RBC burden. Given reports that exosome burden increases with RBC storage [31], a direct in-unit assay may support future studies evaluating whether EXs-RBC burden can contribute to donor-side risk assessment after clinical validation. Potential mitigation strategies—such as exosome reduction—warrant evaluation to enhance transfusion safety and optimize inventory use.

Co-incubation of HMC-1 mast cells with EXs‑RBC demonstrated the immunomodulatory activity of EXs‑RBC, consistent with prior work [28]. Dose-escalated intratracheal challenge, together with mast-cell deficiency or cromolyn treatment, further supports a contributory role of mast cells in EXs-RBC–associated inflammatory lung injury. Findings from intravenous administration of EXs-RBC, which mimics transfusion exposure, suggest that EXs-RBC may represent a transfusion product-derived, non-antibody mediator that amplifies lung inflammation under recipient inflammatory priming and can further aggravate injury in an anti–MHC-I antibody-mediated TRALI-like background. Historically, “second hits” have included donor antibodies [23, 24] and bioactive lipids [60], our findings—consistent with prior reports [28, 58]—suggest that extracellular vesicles in blood products contribute to TRALI. While neutrophils and endothelial cells remain primary contributors to TRALI pathogenesis [61–63], emerging evidence highlights a mast‑cell role in ARDS and TRALI [6, 39, 40], and prior studies have implicated mast cells in antibody‑ and LPS‑induced TRALI [39]. We extend these observations by showing that mast cells may represent a mechanistically relevant link between EXs-RBC and inflammatory lung injury in TRALI-relevant models. Mediators generated during lung injury, such as thrombin, can further activate mast cells [64, 65], and—given TRALI’s complex etiology and the primed activation baseline of mast cells [66]—this pathway could amplify classical, antibody‑mediated TRALI and accelerate disease progression. Accordingly, mast‑cell–directed interventions warrant evaluation in future TRALI mitigation strategies.

Pharmacologic blockade of TLR3 or MAPK (JNK/p38/ERK) and mast-cell stabilization attenuated EXs-RBC–driven lung injury, indicating a potential mechanism- and target-based therapeutic strategy for TRALI. Clinically, TRALI management remains largely supportive and preventive (donor deferral, male-only plasma, leukoreduction, washing or solvent-detergent plasma) [1, 67]. Prior studies indicate that neutrophil-directed approaches [68], endothelial-stabilizing strategies [69], and complement inhibition [70] can ameliorate injury in preclinical TRALI models, yet no mechanism-directed therapy is currently approved [6, 12]. Our data suggest that targeting TLR3 and MAPK signaling could provide therapeutic benefit, and the FDA-approved mast-cell stabilizer cromolyn—recently re-evaluated in ARDS [71, 72]—may also merit exploration in TRALI management, providing a mechanistic rationale for developing mast-cell–directed interventions to treat this life‑threatening complication.

Despite these insights, several limitations warrant mention. First, although TEM/NTA/western blot characterization and exosome-depleted/add-back controls support that the observed effects were mainly associated with the EXs-RBC-enriched fraction, ultracentrifugation-enriched preparations may still contain other extracellular vesicle subtypes or co-isolated protein aggregates. Future studies using exosomes from fresh RBCs, short-storage RBC units, and storage-matched low-exosome RBC units would be clinically meaningful for clarifying how storage duration and exosome burden affect EXs-RBC inflammatory activity. Although DiI/Rab7 colocalization supports late endosomal access of internalized EXs-RBC, this evidence remains spatial and does not by itself identify the ligand responsible for TLR3 engagement. RNase pretreatment implicates RNA-sensitive cargo, but the specific bioactive RNA species in EXs-RBC remain undefined and require future RNA profiling and functional validation. Second, we used human EXs-RBC in murine models; although cross-species uptake and biodistribution of extracellular vesicles have been documented [73], translational caution is warranted. Third, because TRALI is a rapid multicellular inflammatory syndrome, our mast-cell-focused experiments do not exclude contributions from neutrophils, monocytes/macrophages, platelets, complement activation, or the pulmonary endothelium. Moreover, HMC-1 cells are an immature mast-cell leukemia-derived cell line and may not fully recapitulate primary human mast-cell biology. The HMC-1 experiments were designed to assess direct EXs-RBC–mast-cell interactions and do not address activation of other pathogenic cells or cell–cell communication; therefore, validation in primary human mast cells or tissue-resident mast-cell models should be pursued in future studies. Fourth, although cromolyn treatment and Kit^W-sh/W-sh^ mice support a contributory role of mast cells in EXs-RBC–associated inflammatory lung injury, the Kit^W-sh/W-sh^ model has c-Kit–related abnormalities beyond mast-cell deficiency; therefore, future mast-cell-specific genetic or reconstitution approaches will be needed to further validate mast-cell causality. Fifth, because clinically confirmed TRALI-implicated units were not available, we could not directly link the EXs-RBC burden measured in this study to actual post-transfusion TRALI events. Accordingly, this study should be interpreted as a proof-of-concept investigation of analytical feasibility and TRALI-relevant mechanisms. Clinical validation of EXs-RBC burden as a candidate donor-side metric will require further studies using clinically relevant donor–recipient samples. Lastly, owing to specimen availability and the proof-of-concept nature of this platform, the CD63 aptamer sensor was assessed in a limited sample set; therefore, further dedicated technical validation, potentially as a separate technical report, will be required to define its limit of detection, limit of quantification, linear dynamic range, analytical performance, reproducibility, clinically relevant thresholds, and workflow integration before clinical implementation. Nevertheless, our findings support a rapid, isolation-free approach for assessing relative RBC-derived exosome burden directly in stored RBC units and provide mechanistic insight into an EXs-RBC–mast-cell inflammatory axis in TRALI-relevant models. These findings support future evaluation of EXs-RBC burden as a candidate donor-side screening readout and of mast-cell-associated pathways in TRALI research.

## Conclusions

We introduce a CD63 E-AB that exhibits an inverse, concentration-dependent response to EXs-RBC and supports preliminary, rapid assessment of EXs-RBC burden in stored RBC units. We show that EXs-RBC elicit inflammatory cytokine production in HMC-1 mast cells and exacerbate mast-cell–associated lung injury in TRALI-relevant models. Pharmacologic blockade supports the involvement of TLR3 and MAPK signaling, providing mechanistic evidence for an EXs-RBC–mast-cell inflammatory axis. These findings support future evaluation of EXs-RBC burden as a candidate donor-side screening readout and of mast-cell-associated pathways in TRALI research. Future studies should define the causal roles of specific exosomal cargo and validate sensor thresholds, reproducibility, and workflow integration in larger, prospective clinical cohorts.

## Supplementary Information

Supplementary Material 1: DOCX. Supplementary methods, figures, and tables. This file contains detailed experimental procedures, supplementary figures, supplementary tables, reagent information, primer sequences, detailed cell and animal protocols, and additional biochemical analyses supporting the conclusions of this study.

Supplementary Material 2: PDF. Raw image data. This file contains original uncropped western blot images and representative raw image data corresponding to the main figures, including TEM, NTA, fluorescence microscopy, histology, and immunoblot images.

Supplementary Material 3: XLSX. Source data. This file contains the quantitative source data underlying the main and supplementary figures, including qPCR, ELISA, western blot densitometry, and related numerical datasets used for statistical analysis.

## Data Availability

The datasets supporting the conclusions of this article are included within the article and its additional files. Further information is available from the corresponding author on reasonable request.
