# Supplementary material for: CD63 aptamer–based electrochemical sensor assesses stored red blood cell–derived exosome burden associated with mast-cell–mediated transfusion-related acute lung injury
Source: J Nanobiotechnology. 2026 Aug 12;24:1048. doi: 10.1186/s12951-026-04917-x (PMC13749831; doi:10.1186/s12951-026-04917-x)

Figure 2

Figure 2B

NTA

EXs-nor

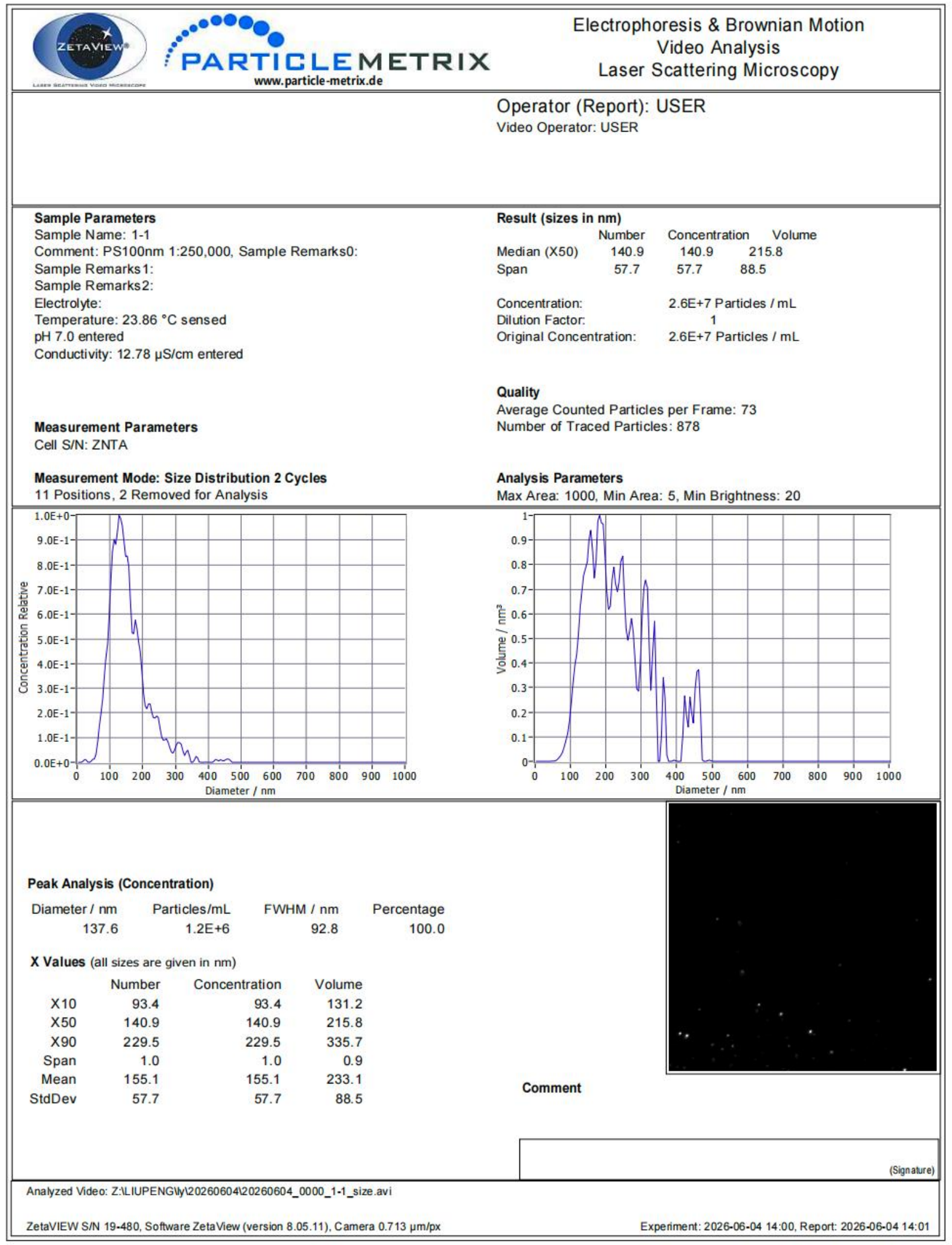

Electrophoresis & Brownian Motion
Video Analysis
Laser Scattering Microscopy

Operator (Report): USER
Video Operator: USER

Sample Parameters
Sample Name: 1-1
Comment: PS100nm 1:250,000, Sample Remarks0:
Sample Remarks1:
Sample Remarks2:
Electrolyte:
Temperature: 23.86 °C sensed
pH 7.0 entered
Conductivity: 12.78 µS/cm entered

Result (sizes in nm)

	Number	Concentration	Volume
Median (X50)	140.9	140.9	215.8
Span	57.7	57.7	88.5

Concentration: 2.6E+7 Particles / mL
Dilution Factor: 1
Original Concentration: 2.6E+7 Particles / mL

Quality
Average Counted Particles per Frame: 73
Number of Traced Particles: 878

Measurement Parameters
Cell S/N: ZNTA

Measurement Mode: Size Distribution 2 Cycles
11 Positions, 2 Removed for Analysis

Analysis Parameters
Max Area: 1000, Min Area: 5, Min Brightness: 20

Peak Analysis (Concentration)

Diameter / nm	Particles/mL	FWHM / nm	Percentage
137.6	1.2E+6	92.8	100.0

X Values (all sizes are given in nm)

	Number	Concentration	Volume
X10	93.4	93.4	131.2
X50	140.9	140.9	215.8
X90	229.5	229.5	335.7
Span	1.0	1.0	0.9
Mean	155.1	155.1	233.1
StdDev	57.7	57.7	88.5

Comment

(Signature)

Analyzed Video: Z:\LIUPENG\ly\20260604\20260604_0000_1-1_size.avi

ZetaVIEW S/N 19-480, Software ZetaView (version 8.05.11), Camera 0.713 µm/px

Experiment: 2026-06-04 14:00, Report: 2026-06-04 14:01

EXs-RBC

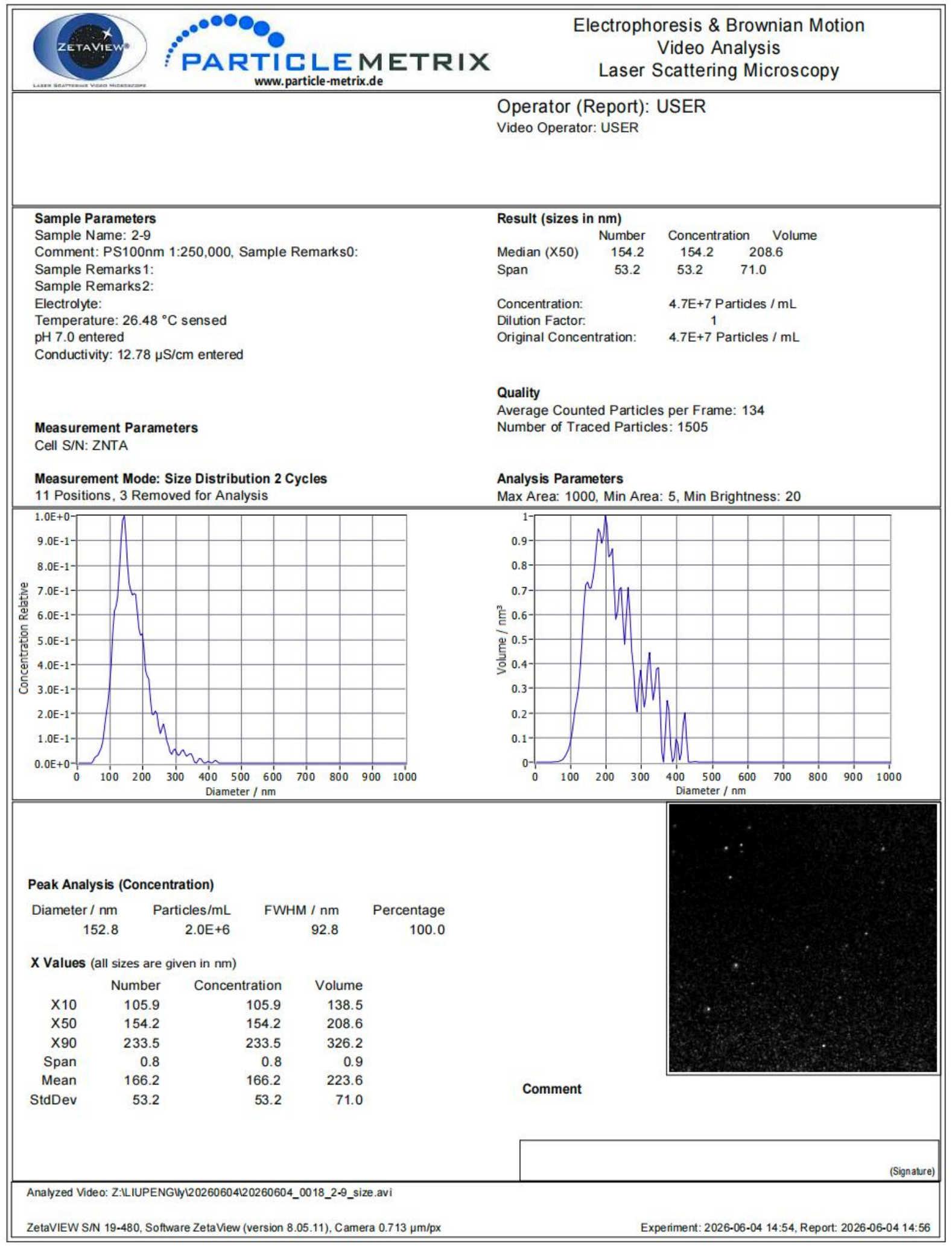

Electrophoresis & Brownian Motion
Video Analysis
Laser Scattering Microscopy

Operator (Report): USER
Video Operator: USER

Sample Parameters
Sample Name: 2-9
Comment: PS100nm 1:250,000, Sample Remarks0:
Sample Remarks1:
Sample Remarks2:
Electrolyte:
Temperature: 26.48 °C sensed
pH 7.0 entered
Conductivity: 12.78 µS/cm entered

Result (sizes in nm)

	Number	Concentration	Volume
Median (X50)	154.2	154.2	208.6
Span	53.2	53.2	71.0

Concentration: 4.7E+7 Particles / mL
Dilution Factor: 1
Original Concentration: 4.7E+7 Particles / mL

Quality
Average Counted Particles per Frame: 134
Number of Traced Particles: 1505

Measurement Parameters
Cell S/N: ZNTA

Measurement Mode: Size Distribution 2 Cycles
11 Positions, 3 Removed for Analysis

Analysis Parameters
Max Area: 1000, Min Area: 5, Min Brightness: 20

Peak Analysis (Concentration)

Diameter / nm	Particles/mL	FWHM / nm	Percentage
152.8	2.0E+6	92.8	100.0

X Values (all sizes are given in nm)

	Number	Concentration	Volume
X10	105.9	105.9	138.5
X50	154.2	154.2	208.6
X90	233.5	233.5	326.2
Span	0.8	0.8	0.9
Mean	166.2	166.2	223.6
StdDev	53.2	53.2	71.0

Comment

(Signature)

Analyzed Video: Z:\LIUPENG\ly\20260604\20260604_0018_2-9_size.avi

ZetaVIEW S/N 19-480, Software ZetaView (version 8.05.11), Camera 0.713 µm/px

Experiment: 2026-06-04 14:54, Report: 2026-06-04 14:56

Figure 2D

TEM

EXs-nor EXs-RBC

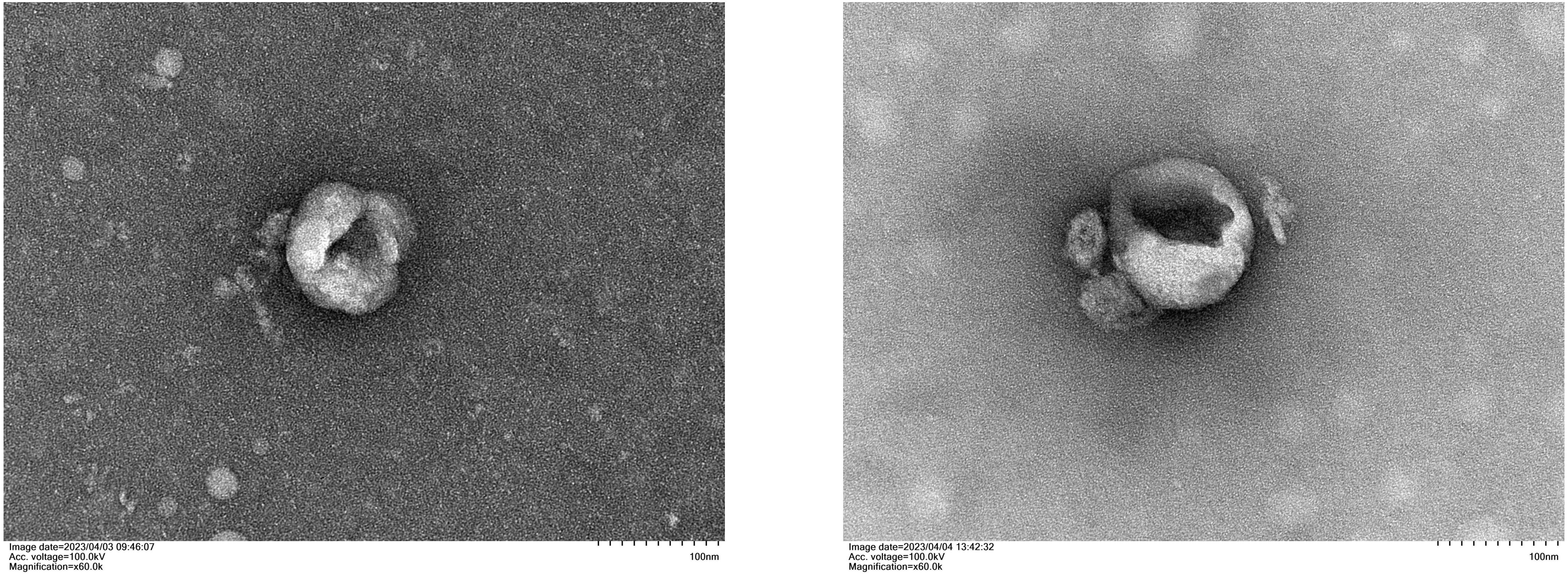

Figure 2E

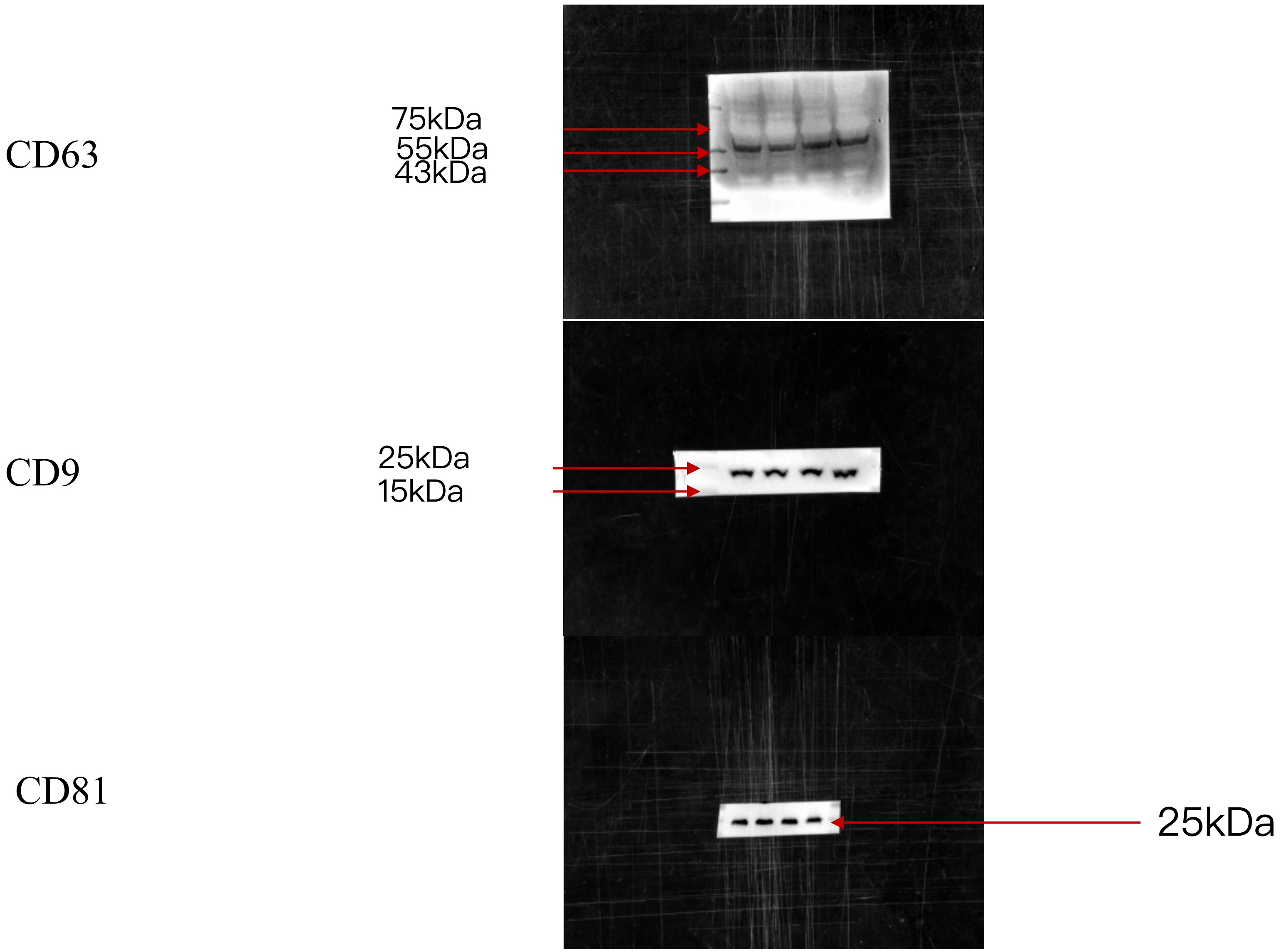

Figure 2E

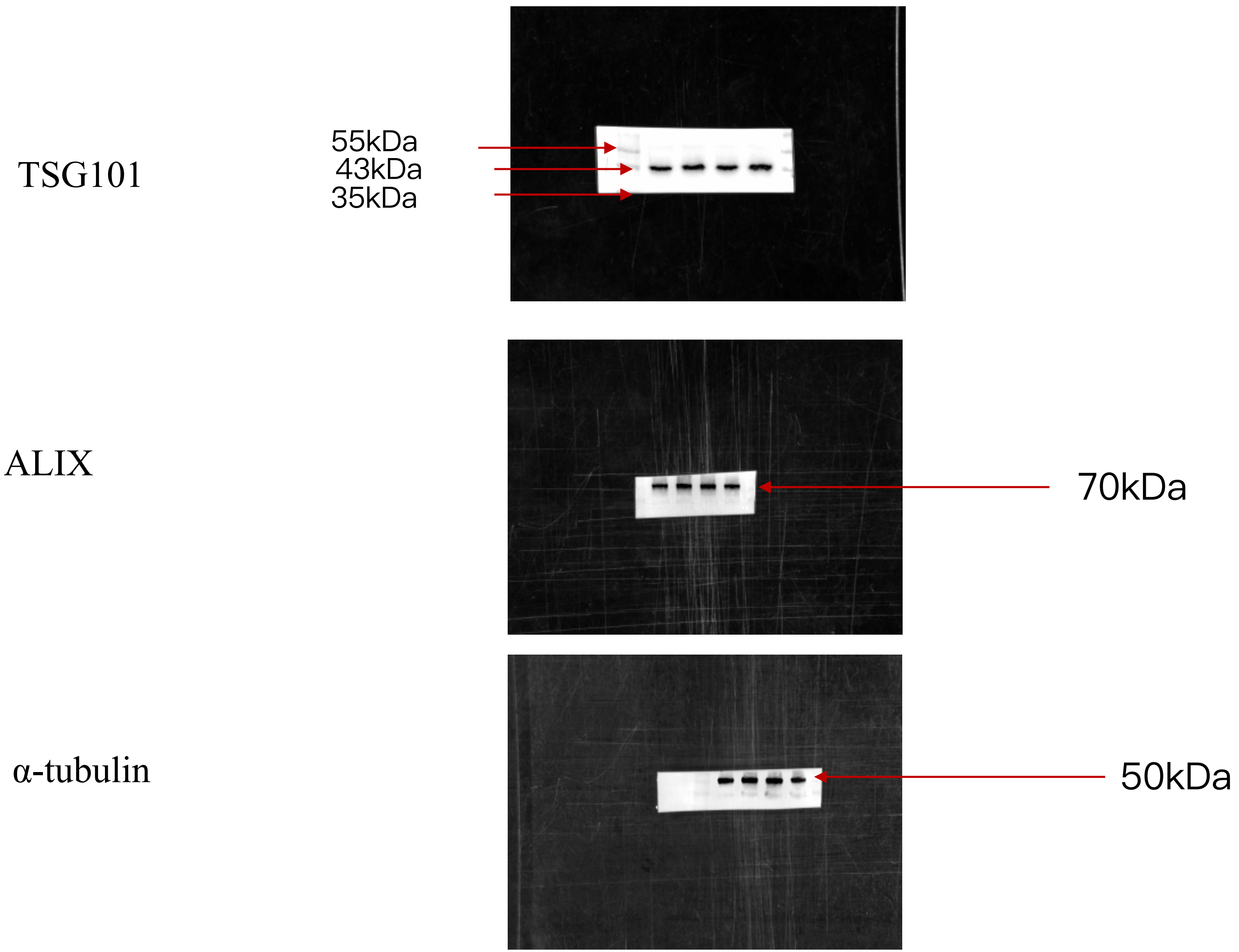

Figure 2E

Calnexin

130kDa
100kDa
75kDa

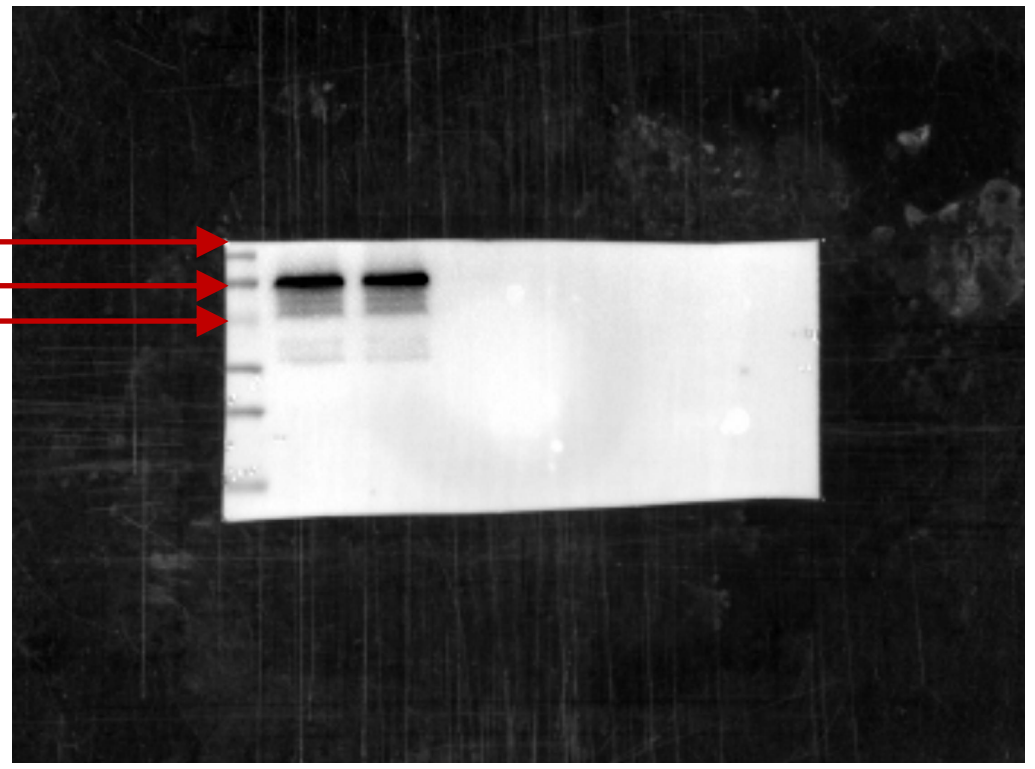

tubulin

55kDa

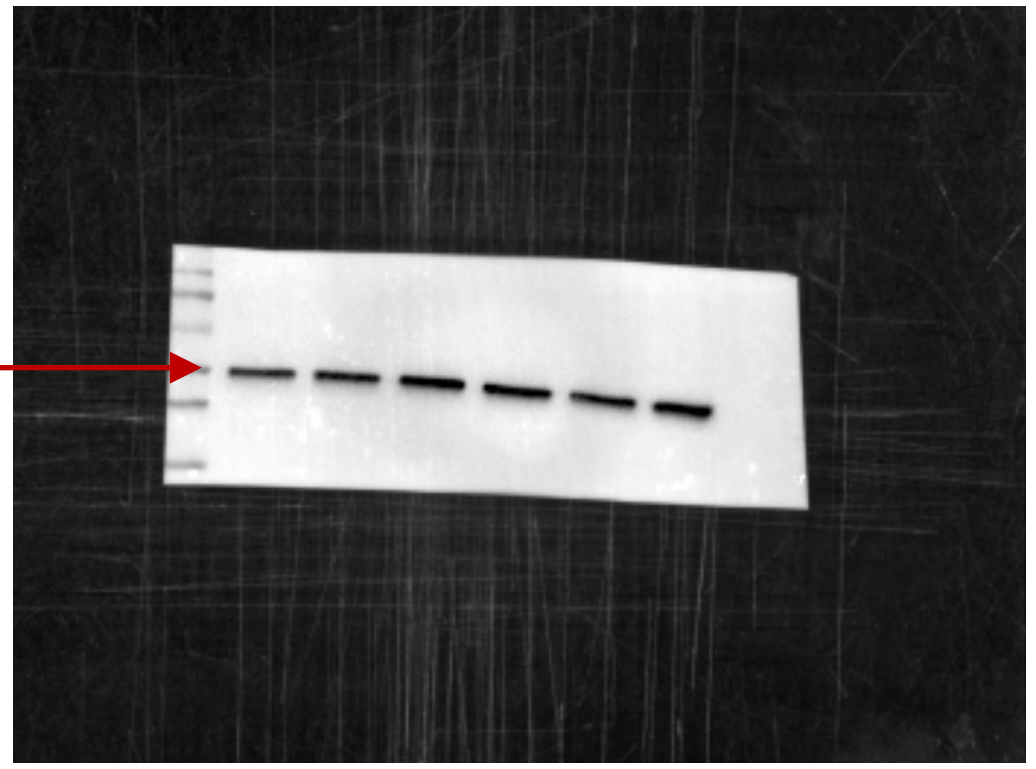

Figure 2F

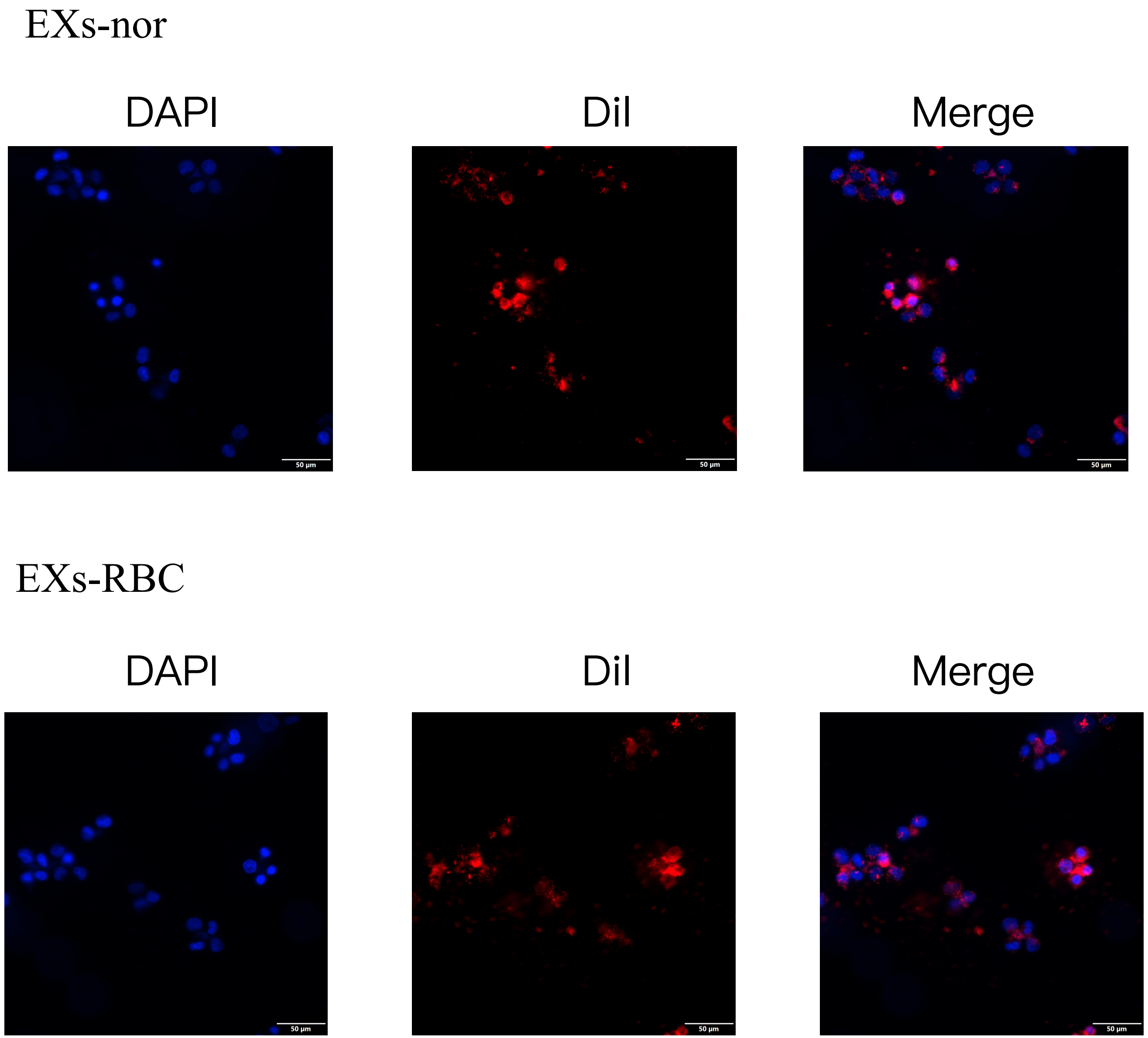

Figure 4

Figure 4B

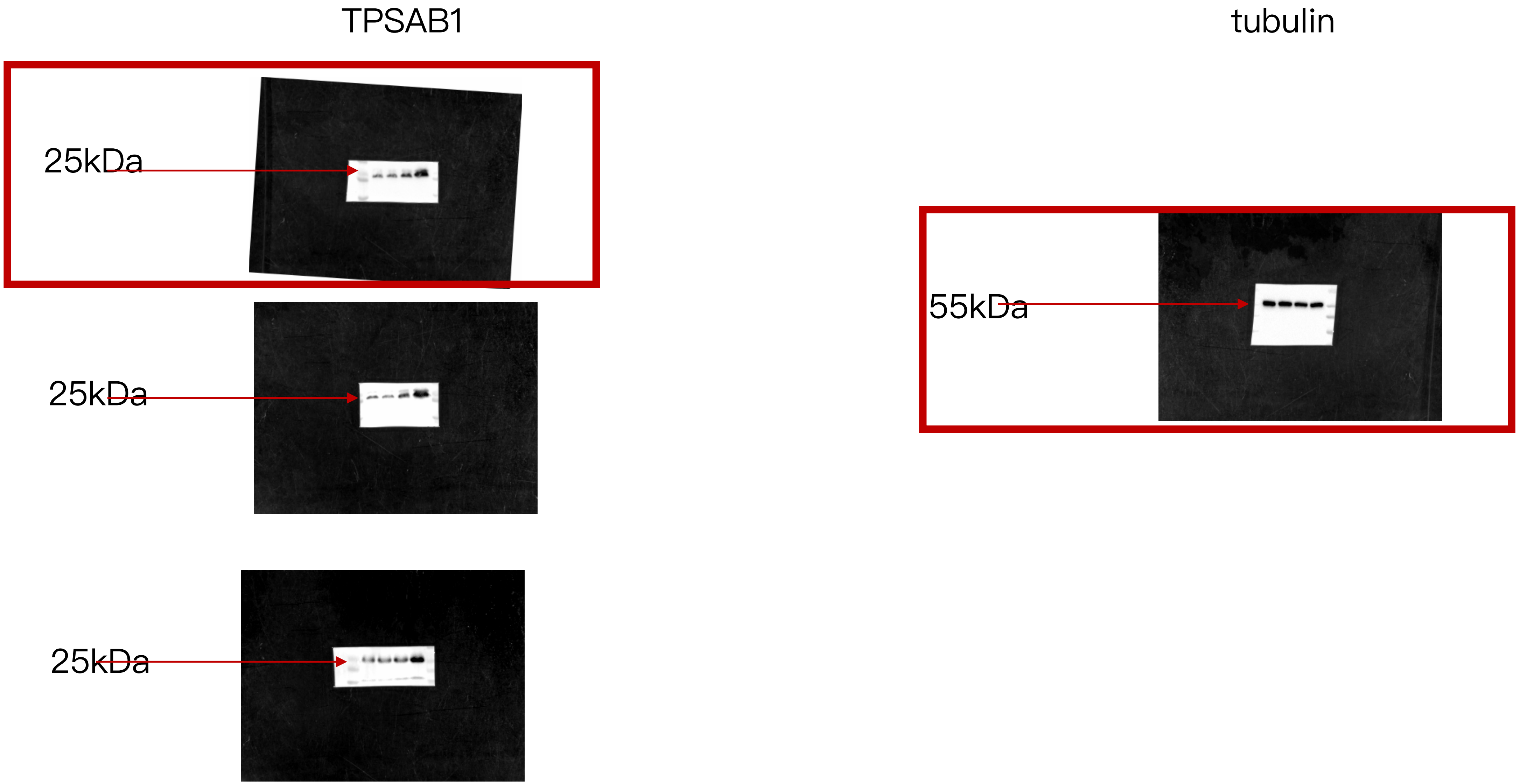

Figure 4I

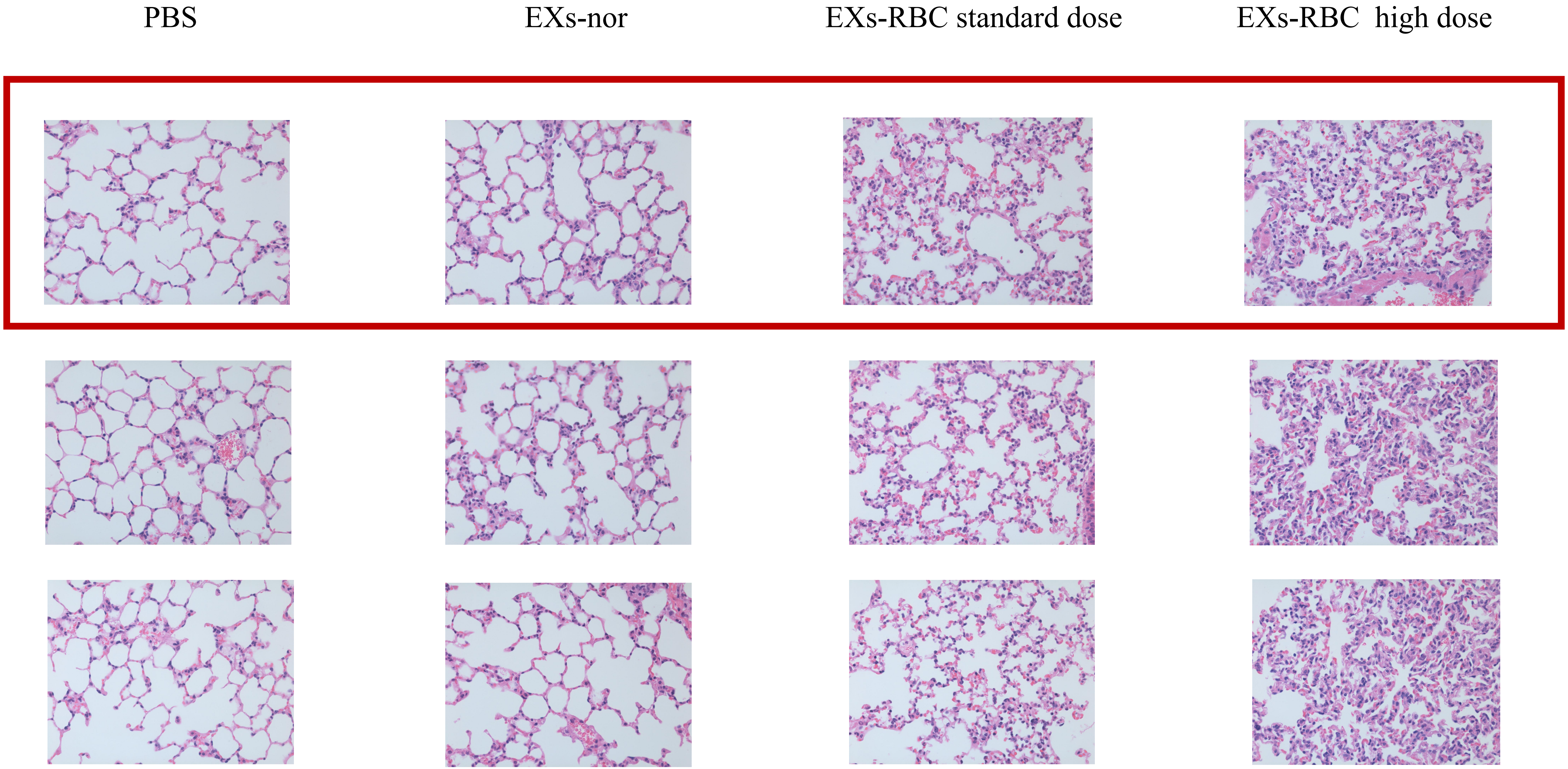

Figure 5

Figure 5J

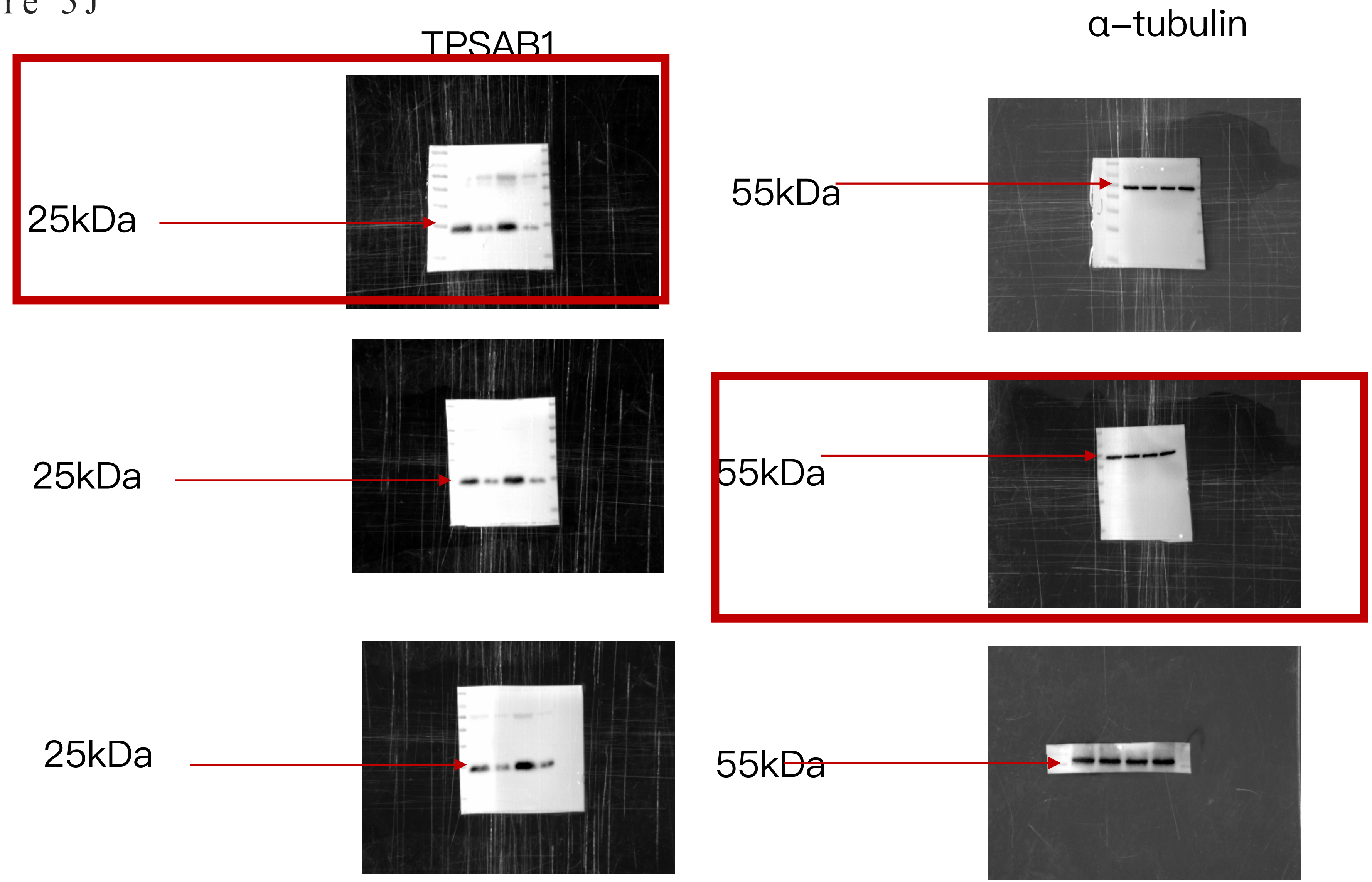

Figure 5N

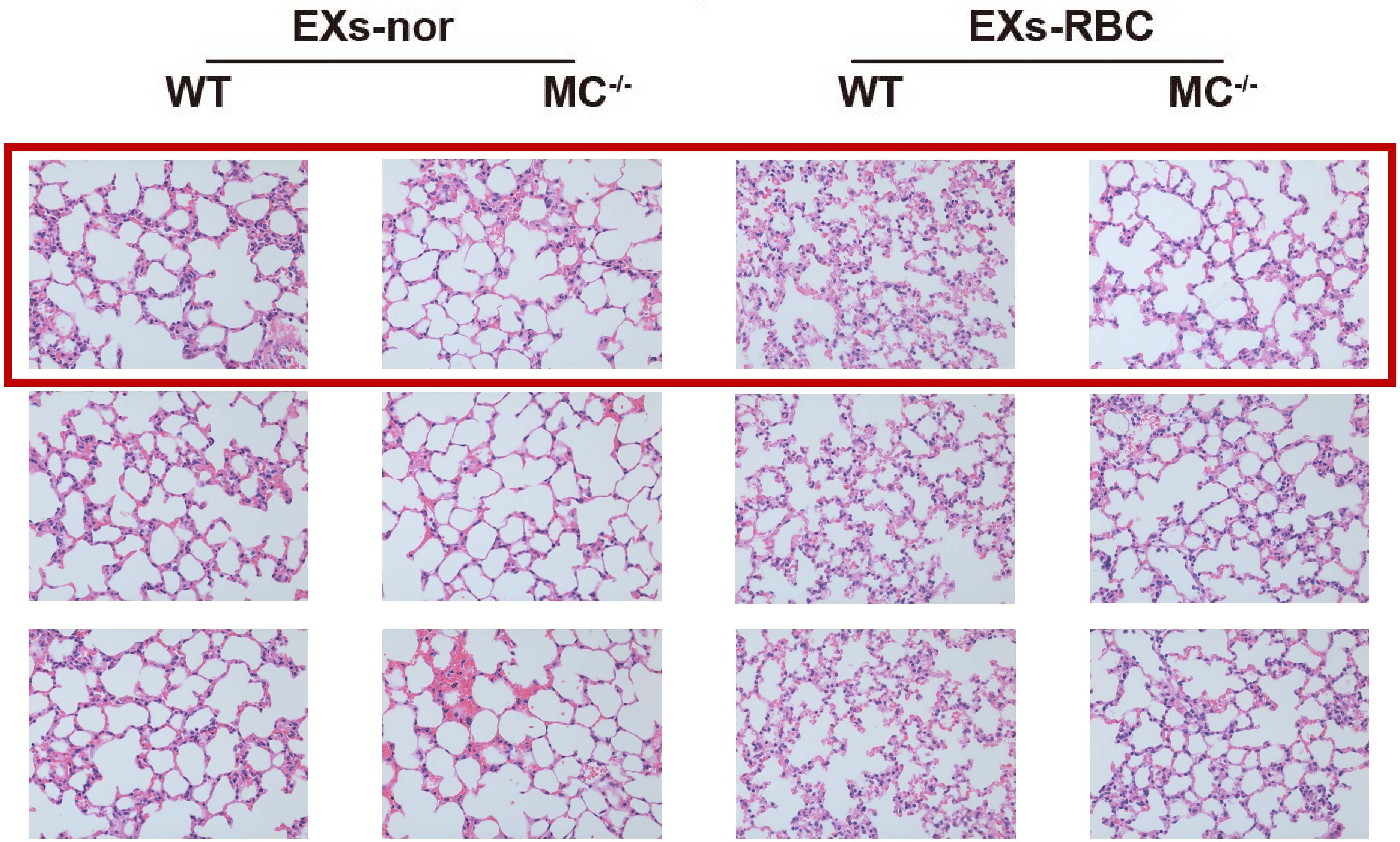

Figure 6

Figure 6B

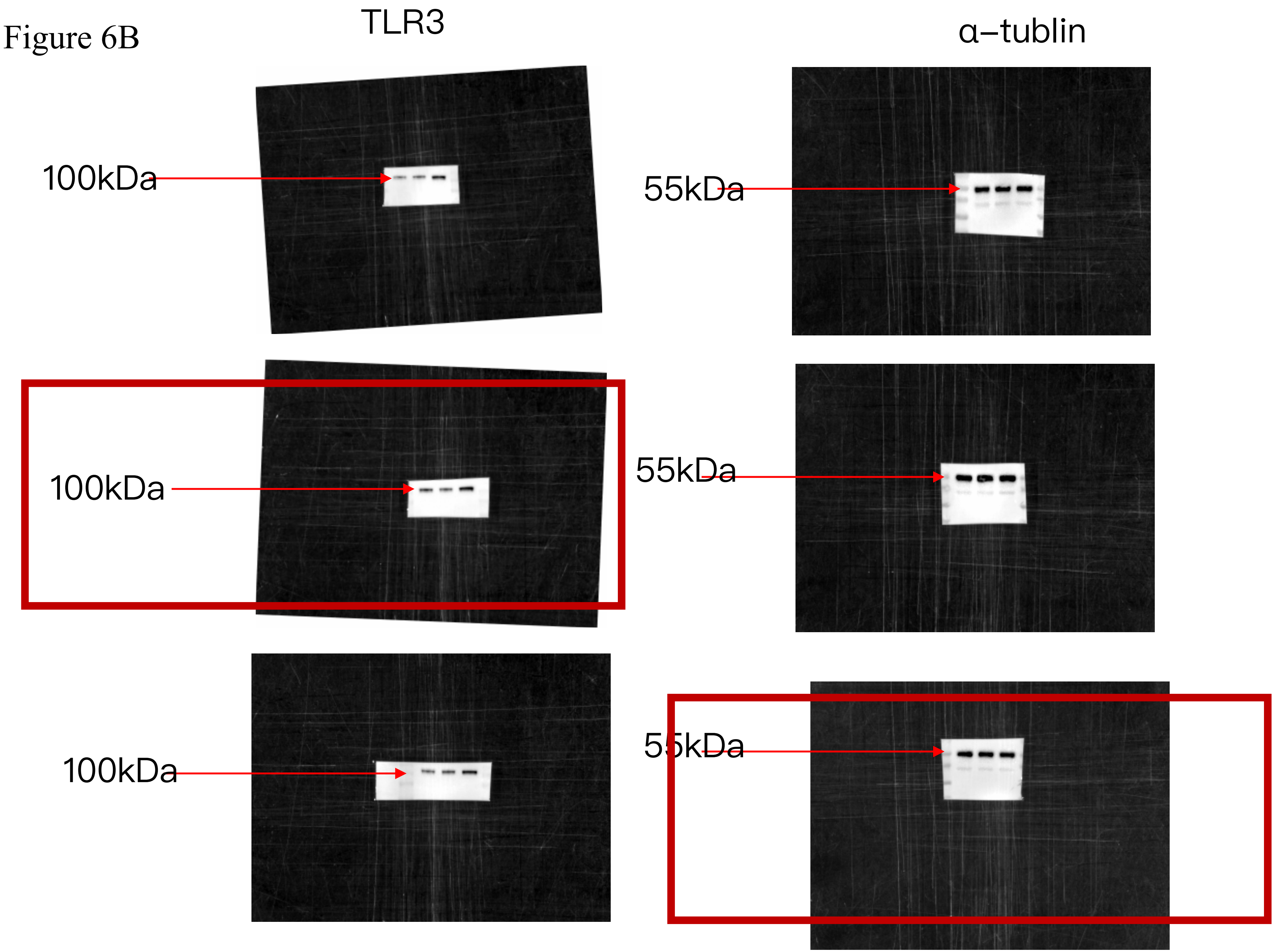

Figure 6C

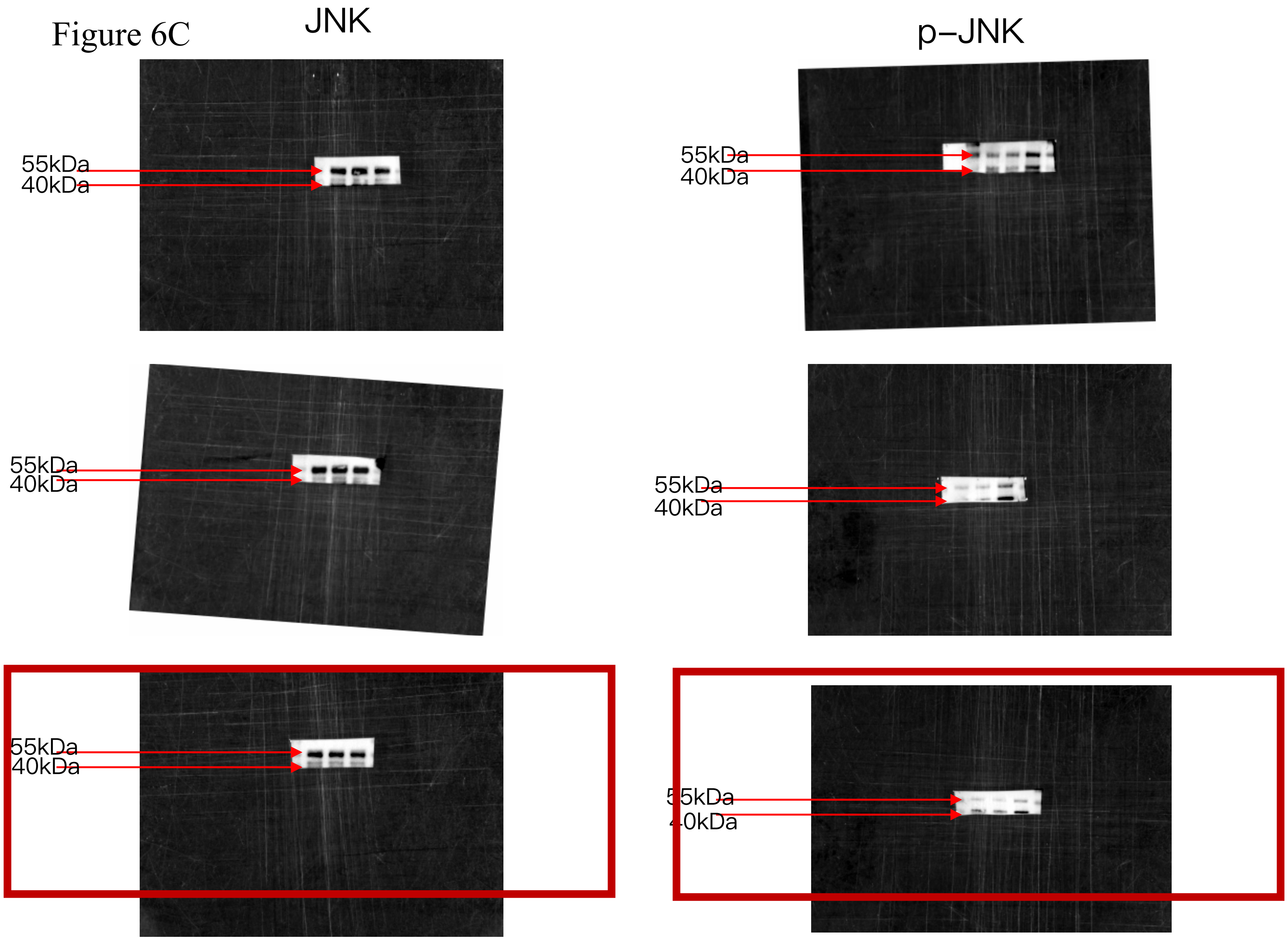
JNK
p−JNK
55kDa
40kDa
55kDa
40kDa
55kDa
40kDa
55kDa
40kDa
55kDa
40kDa
55kDa
40kDa

Figure 6C

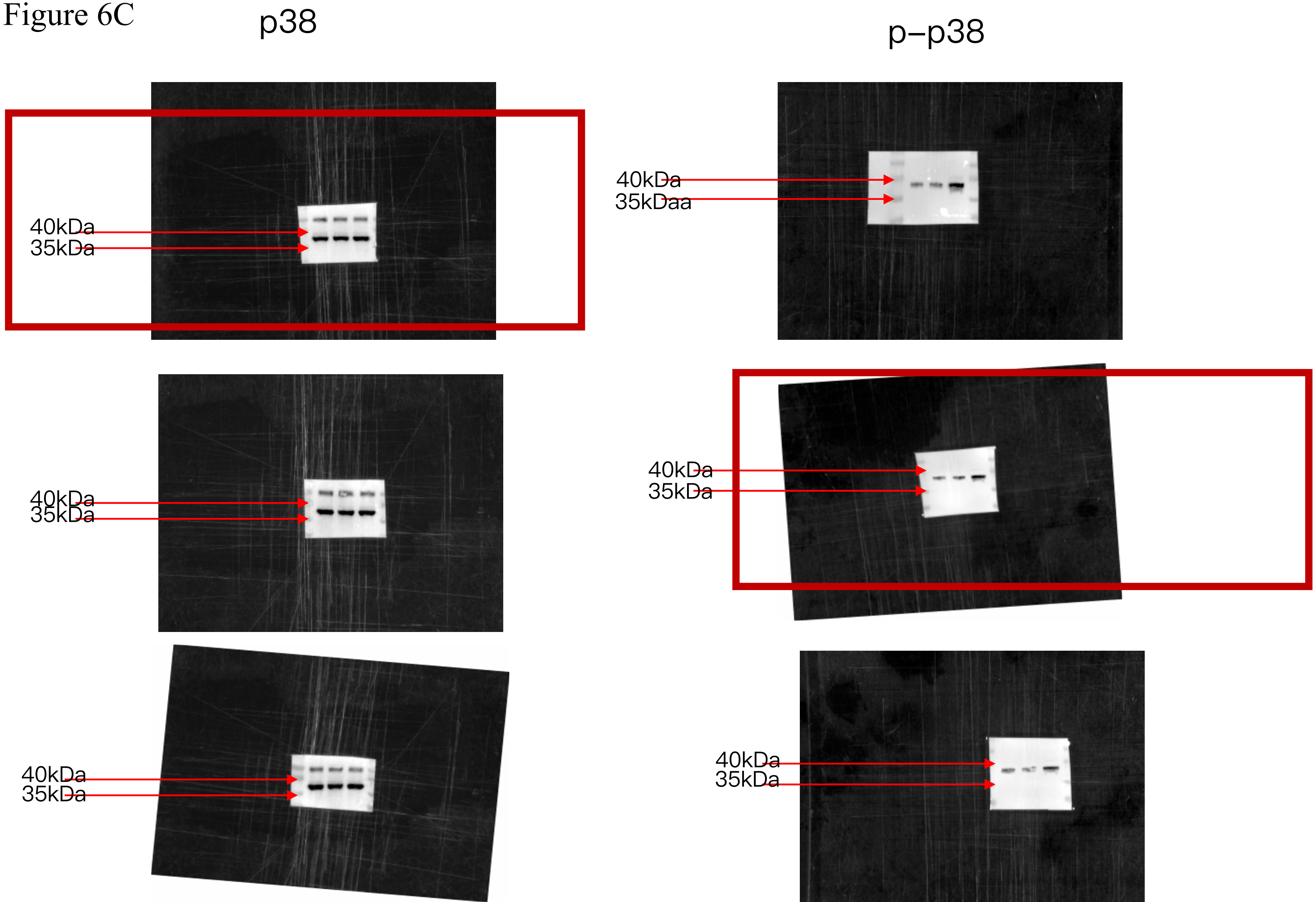
p38
p−p38
40kDa
35kDa
40kDa
35kDaa
40kDa
35kDa
40kDa
35kDa
40kDa
35kDa
40kDa
35kDa

Figure 6C

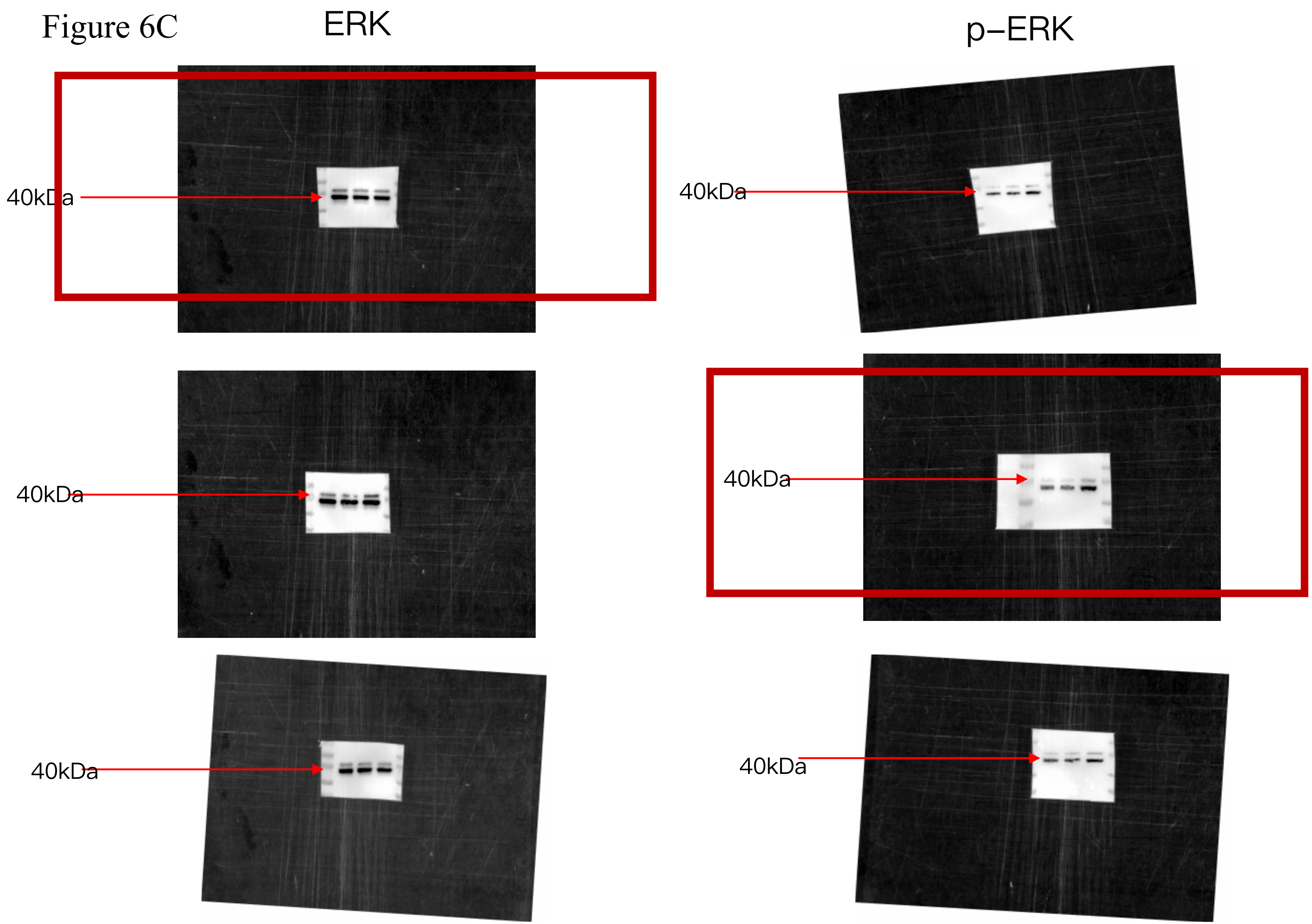

Figure 6J

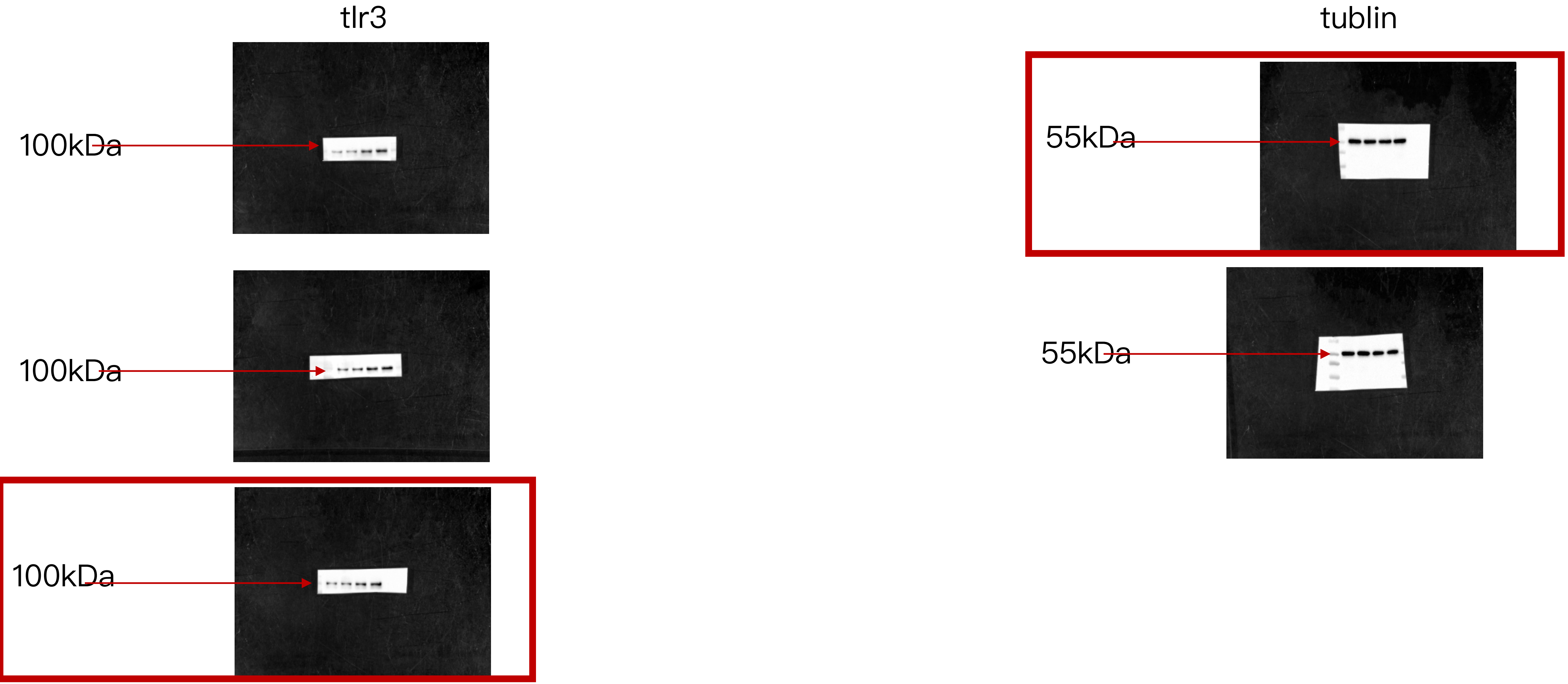

Figure 6L

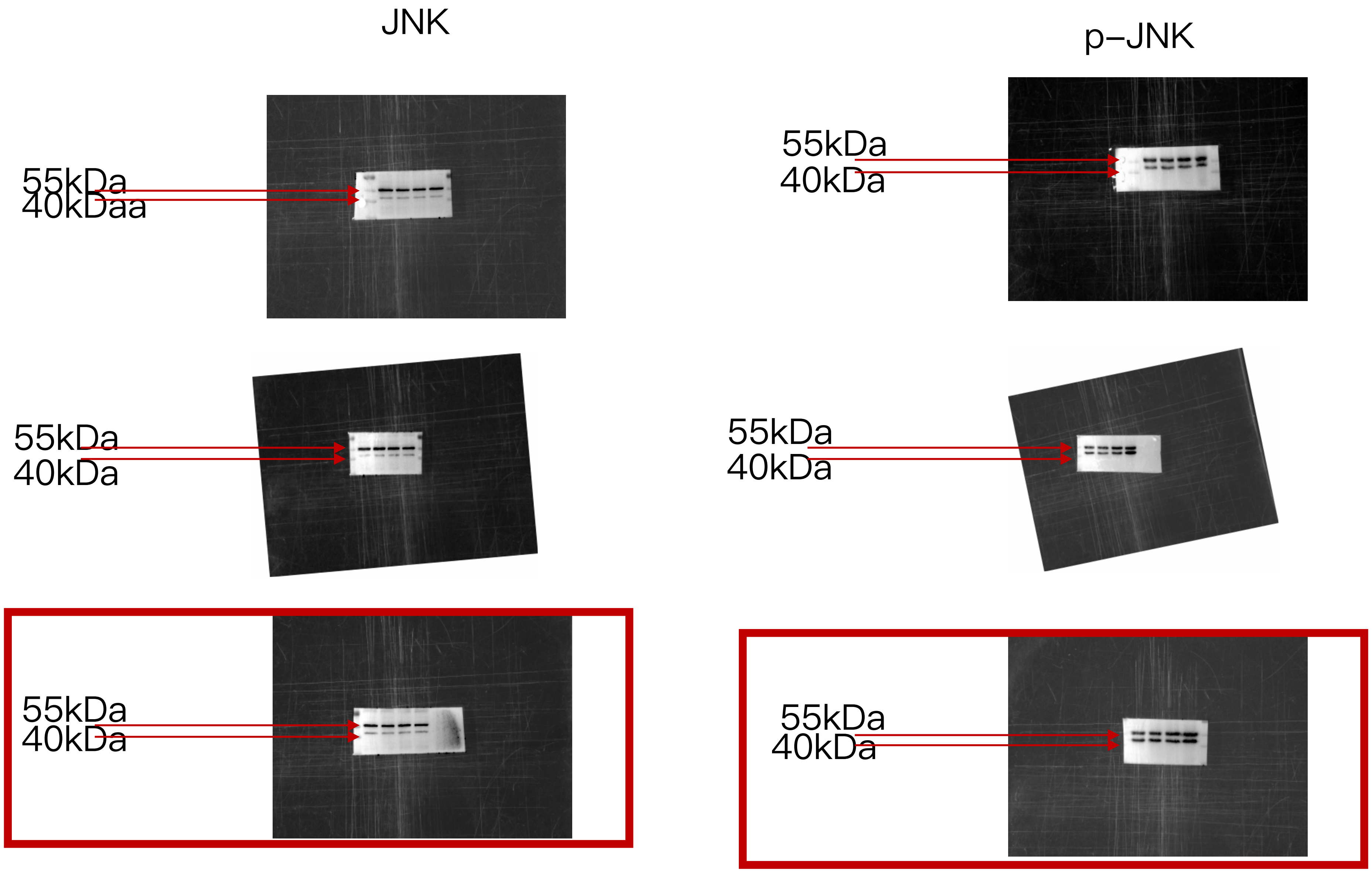
JNK
p–JNK
55kDa
40kDaa
55kDa
40kDa
55kDa
40kDa
55kDa
40kDa
55kDa
40kDa
55kDa
40kDa

Figure 6L

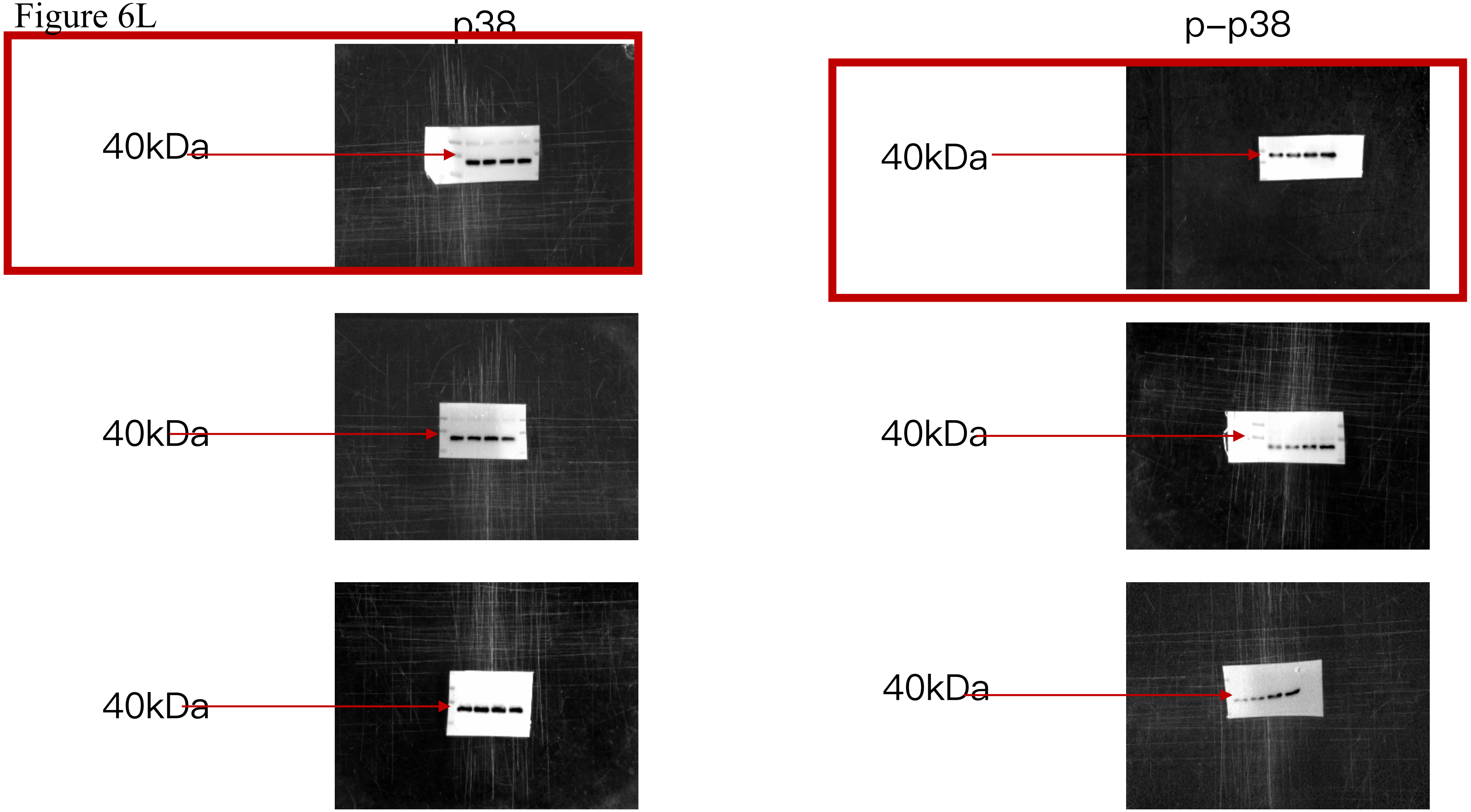

Figure 6L

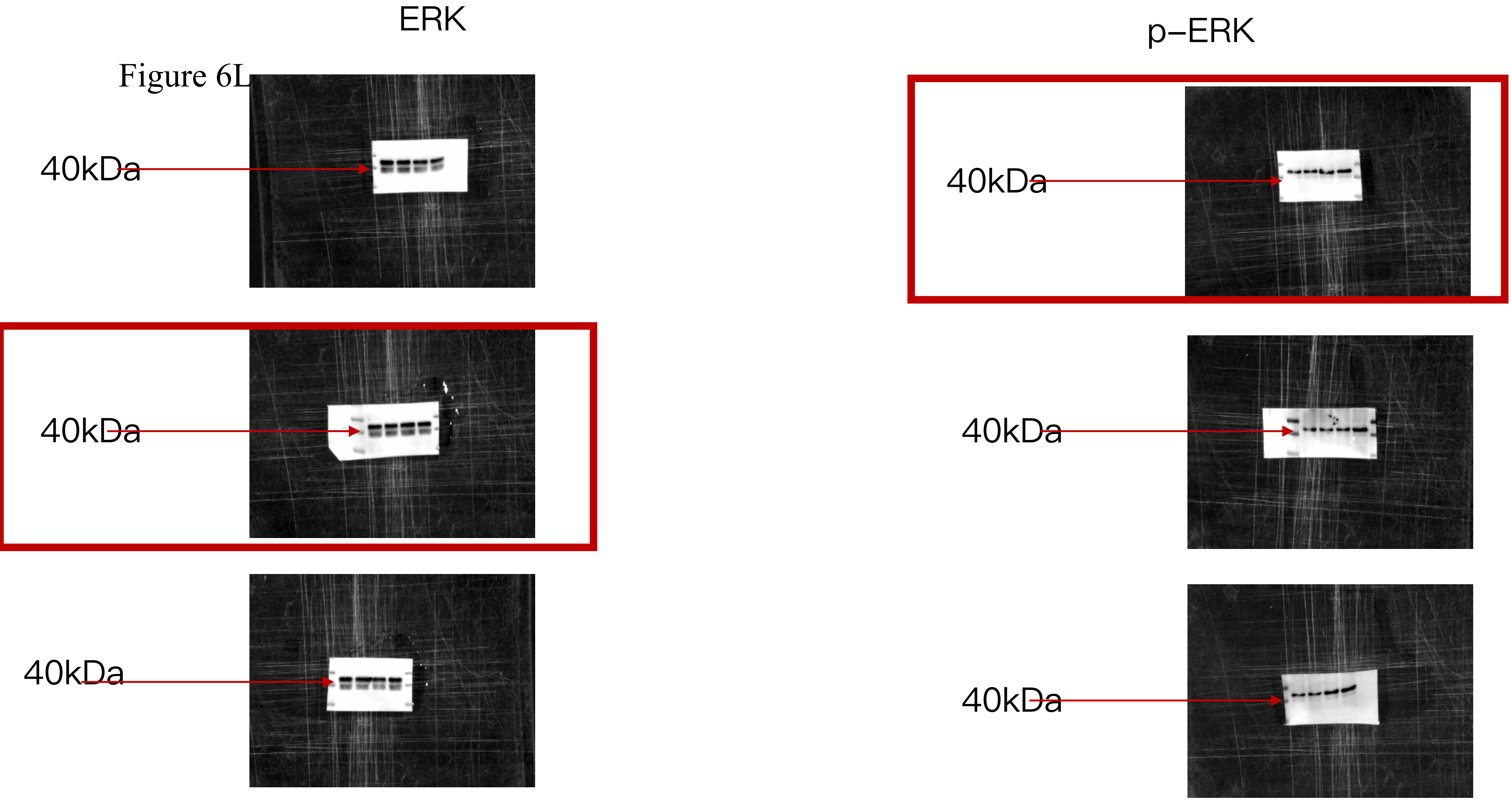

Figure 6O

tlr3

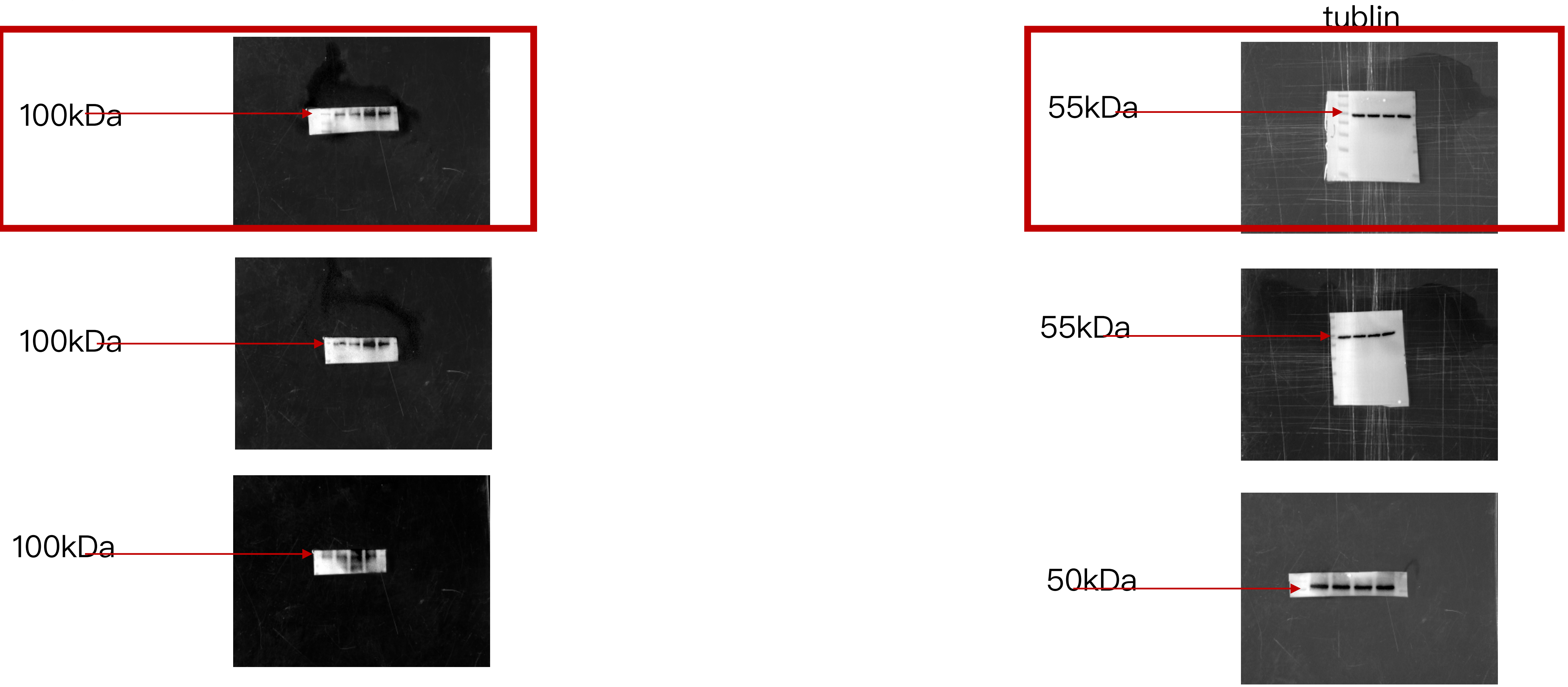

Figure 6P

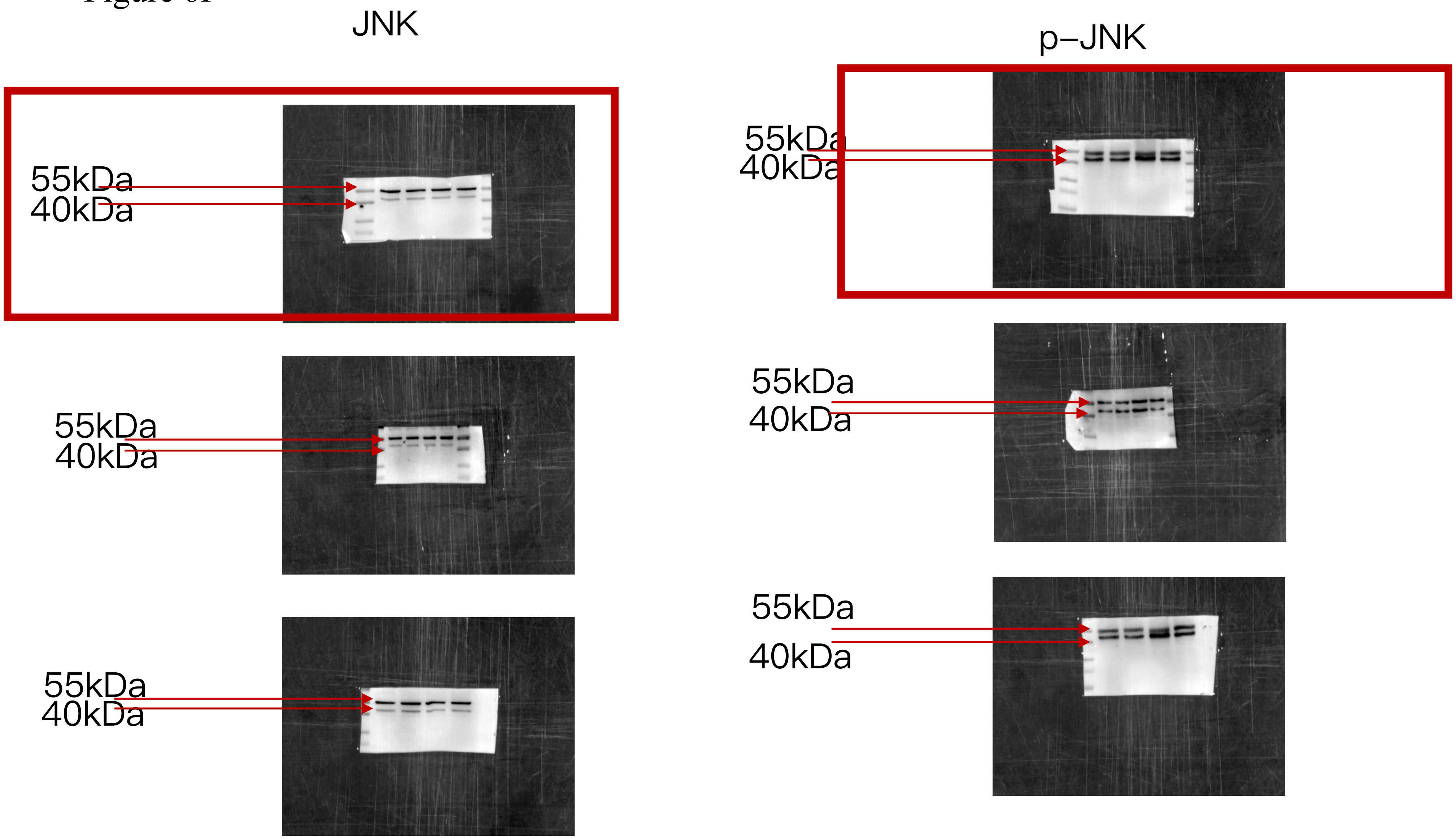
JNK
55kDa
40kDa
55kDa
40kDa
55kDa
40kDa
p–JNK
55kDa
40kDa
55kDa
40kDa
55kDa
40kDa

Figure 6P

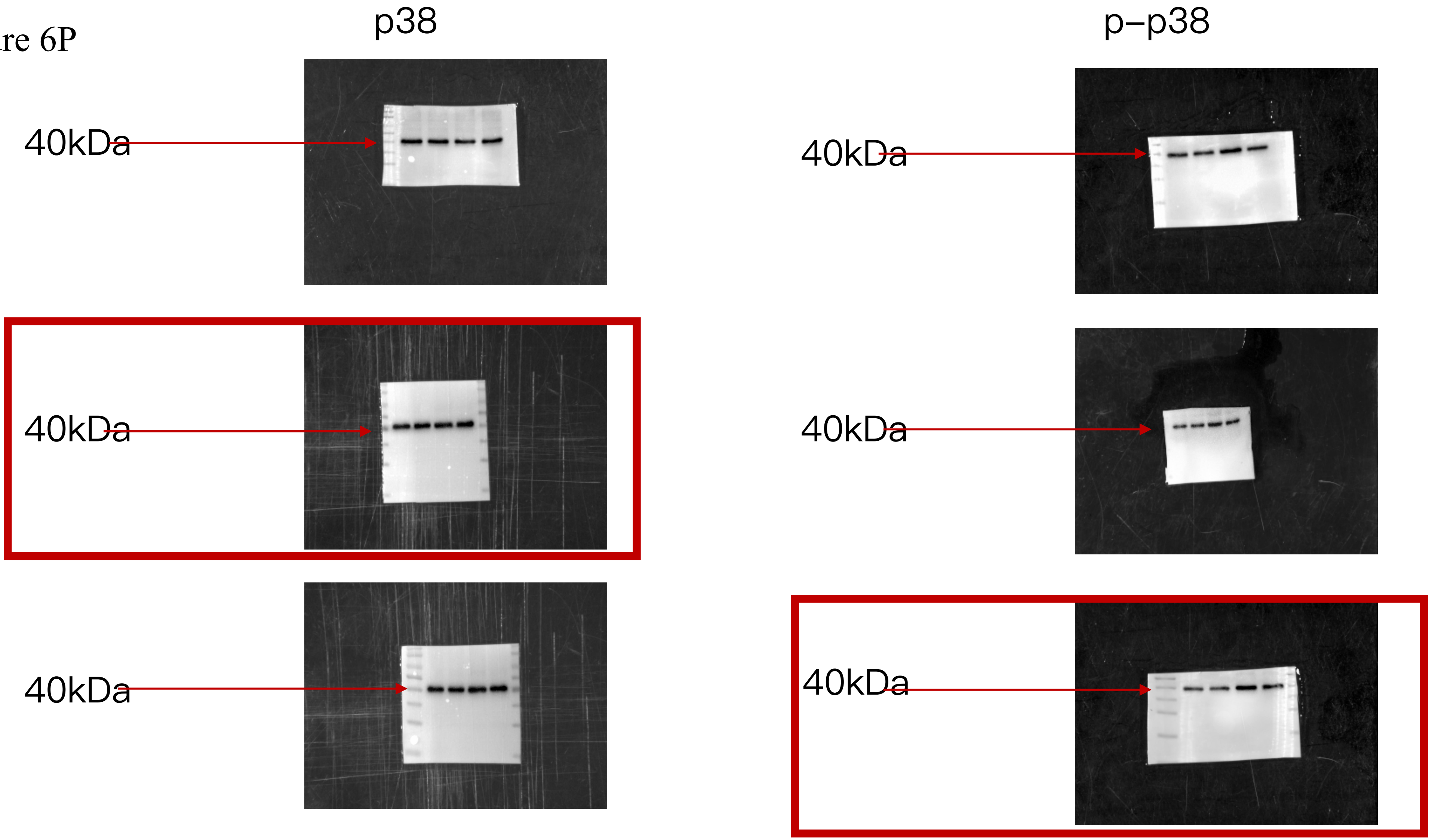

Figure 6P

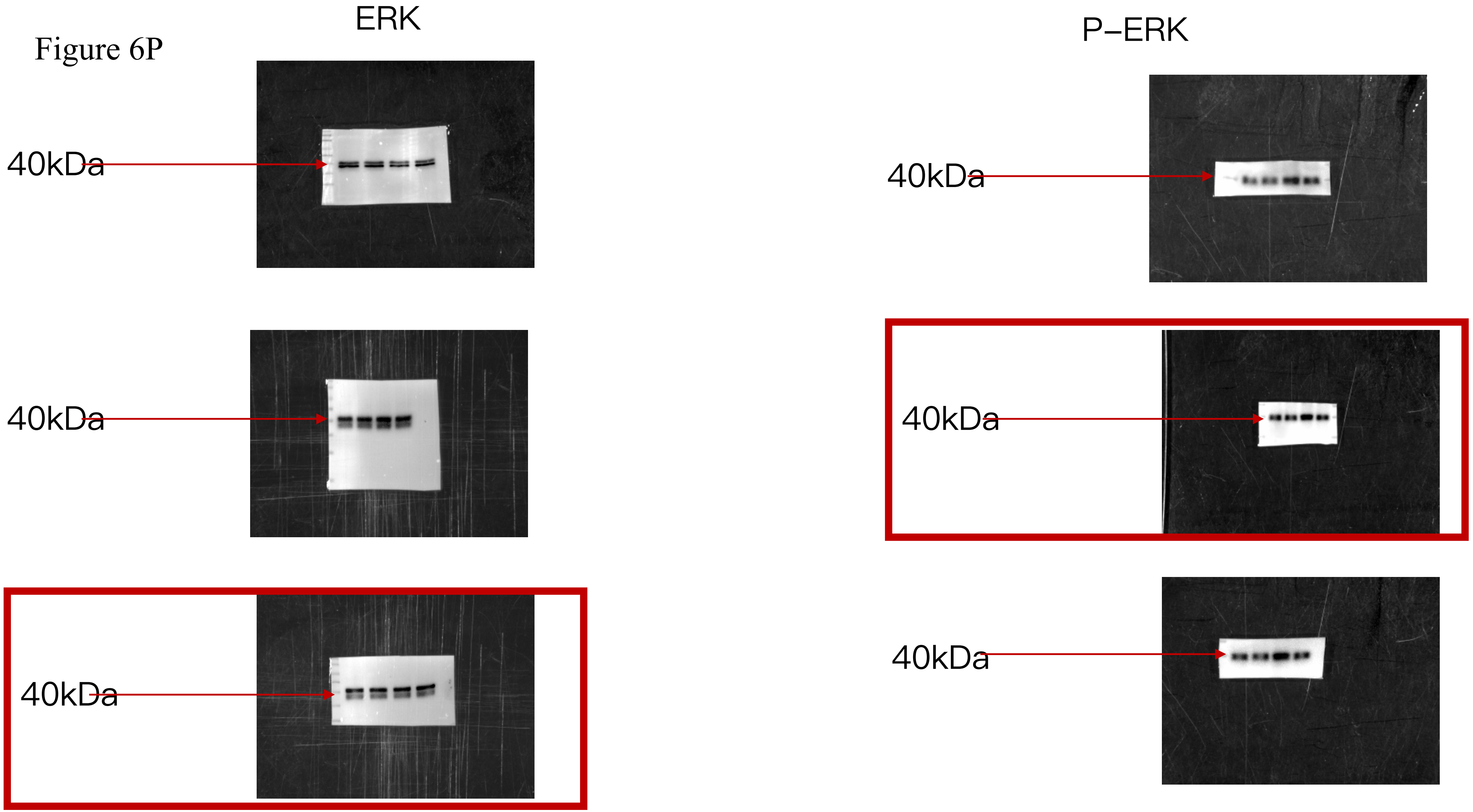

Figure 8

Figure 8O

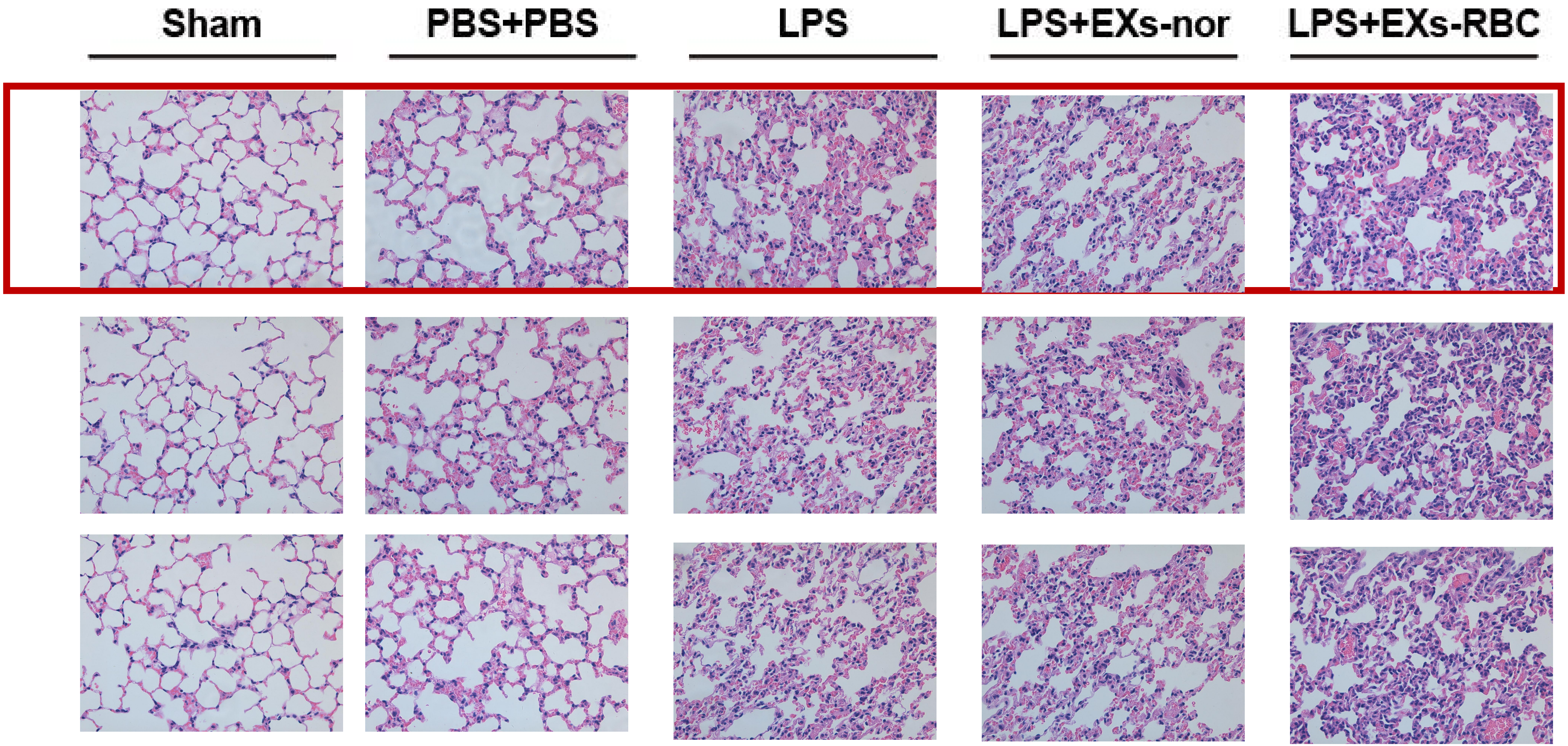

Figure 9

Figure 9O

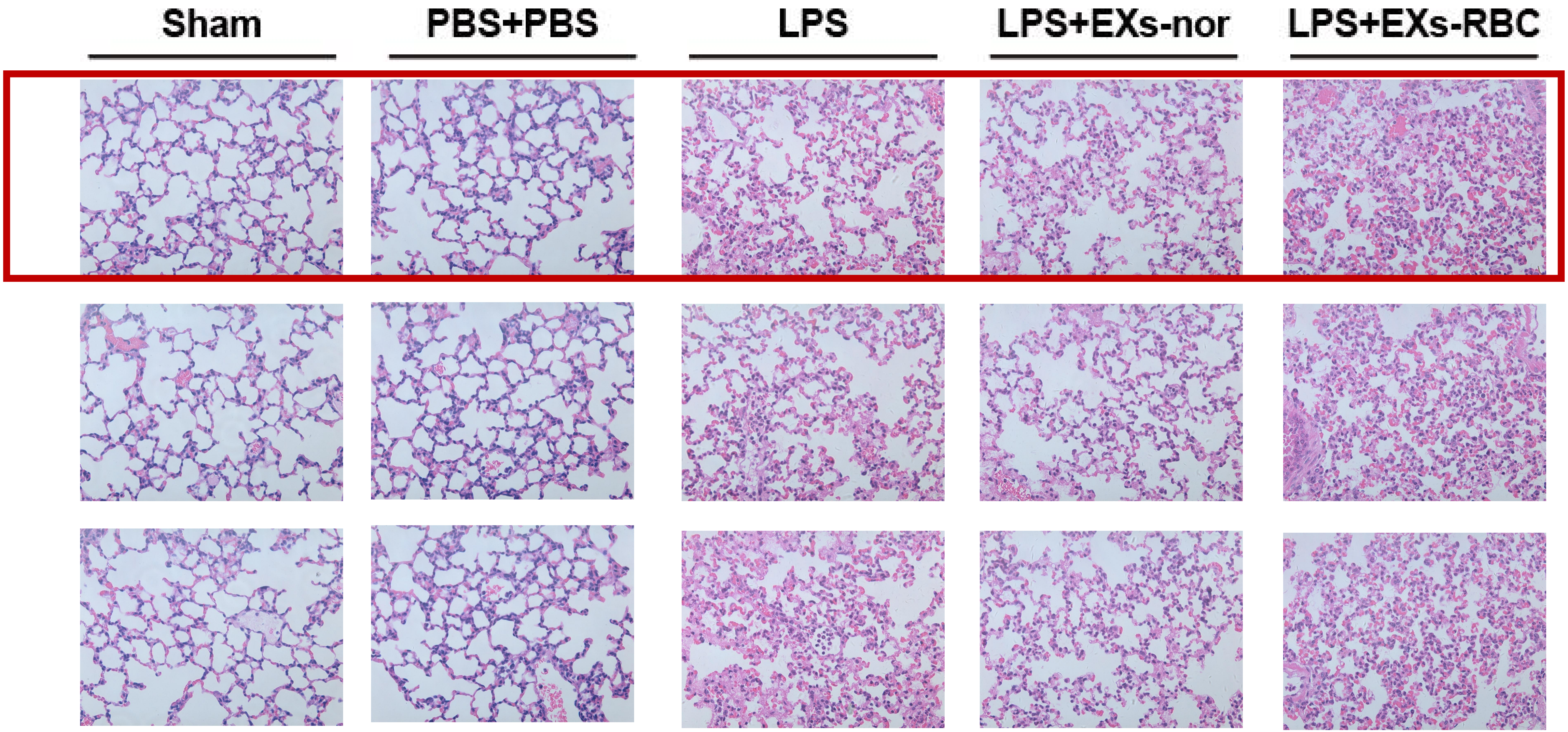

Figure 10

Figure 10M

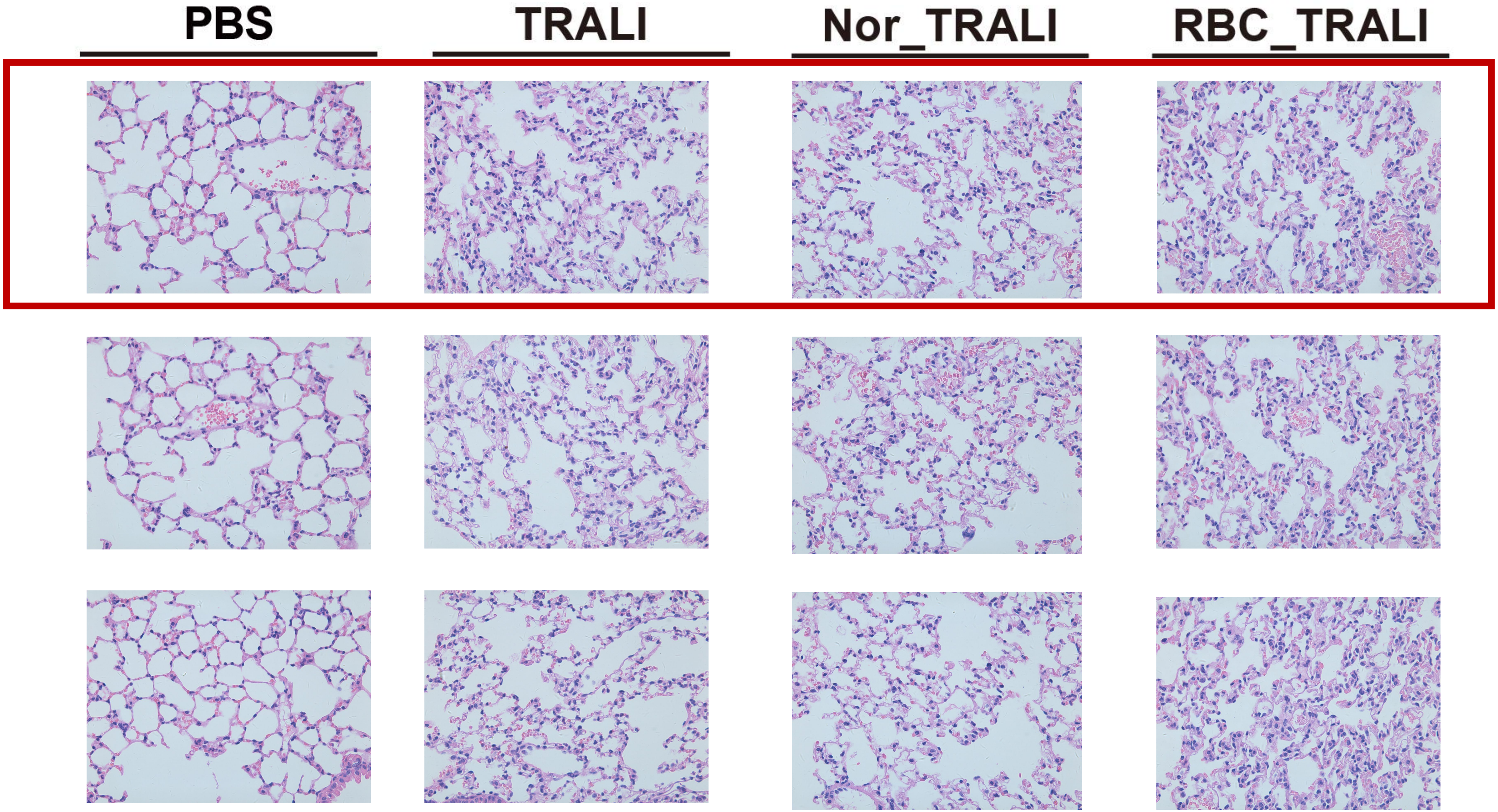

Figure S4

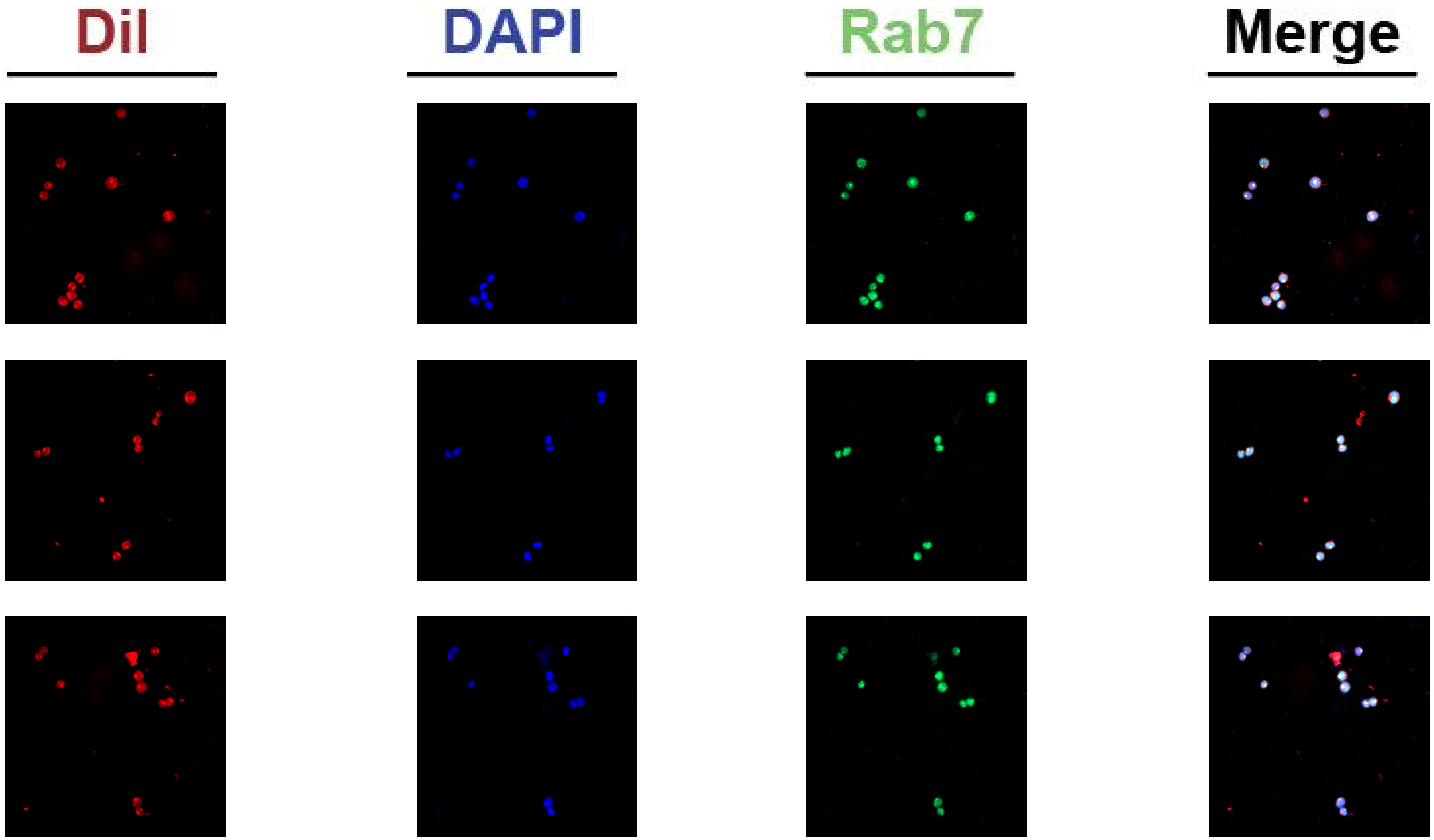
DiI
DAPI
Rab7
Merge

Figure S5

Figure S5H

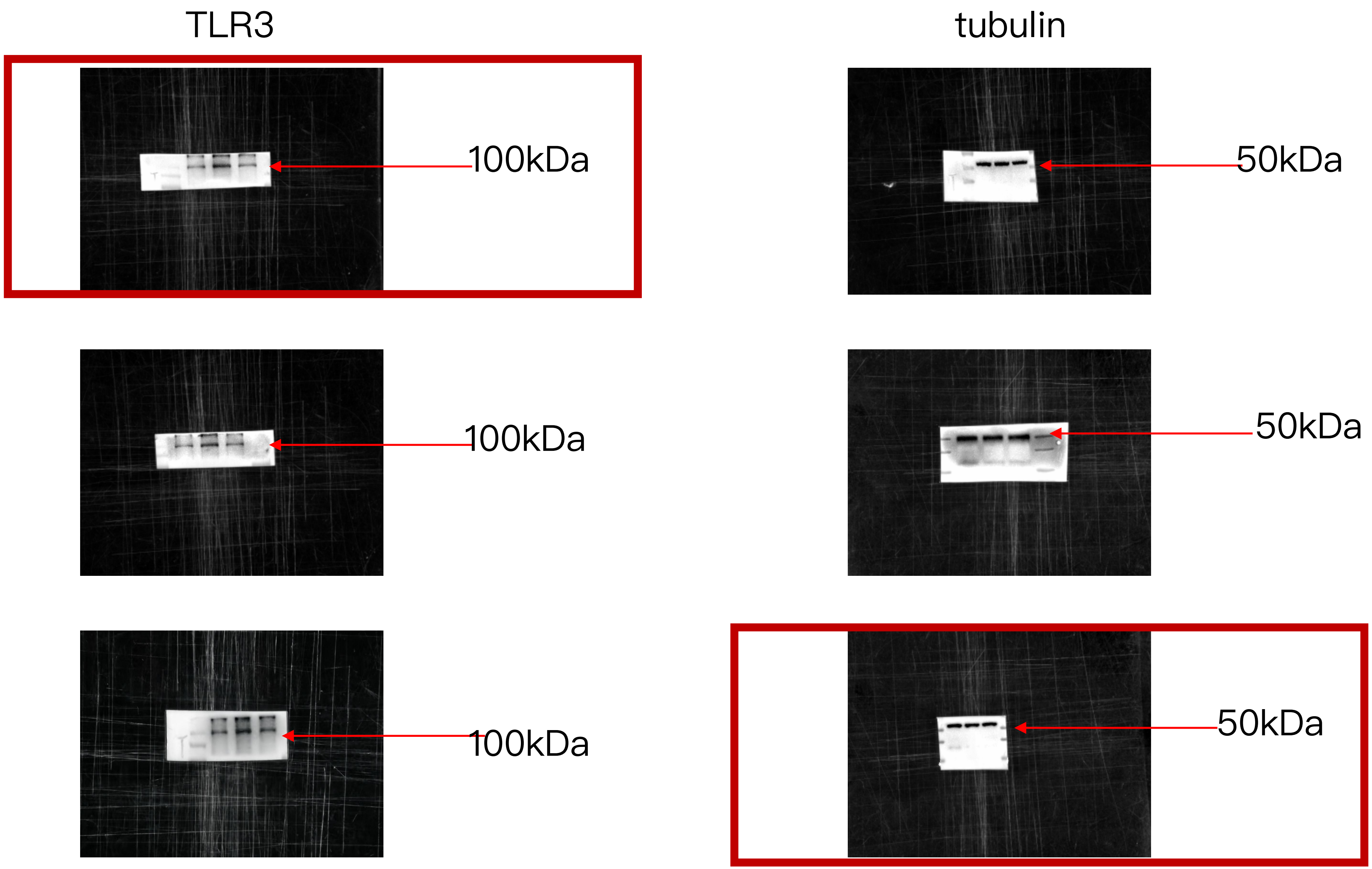

Figure S5I

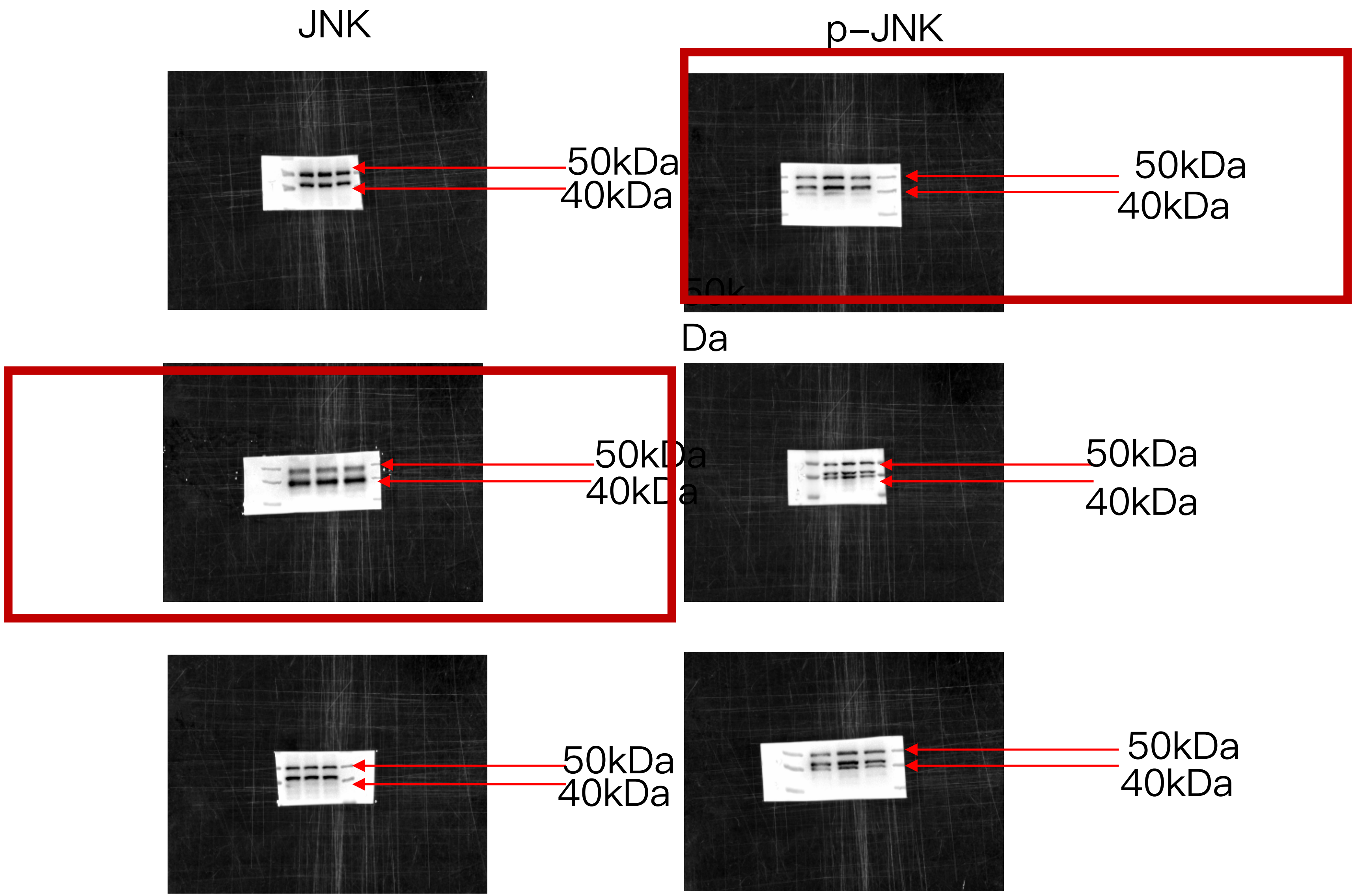

JNK
p–JNK
50kDa
40kDa
50kDa
40kDa
50k
Da
50kDa
40kDa
50kDa
40kDa
50kDa
40kDa
50kDa
40kDa

Figure S5I

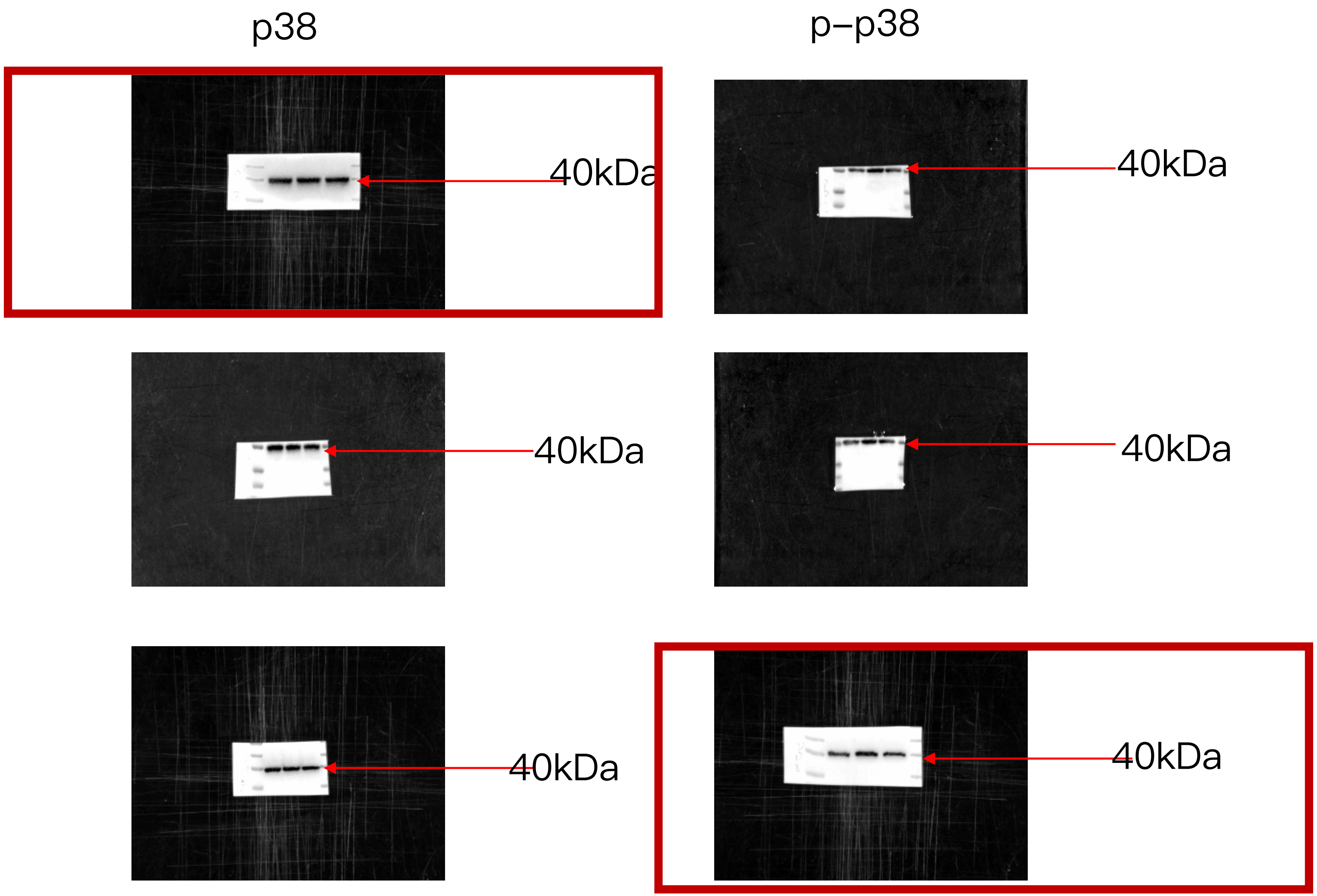

Figure S5I

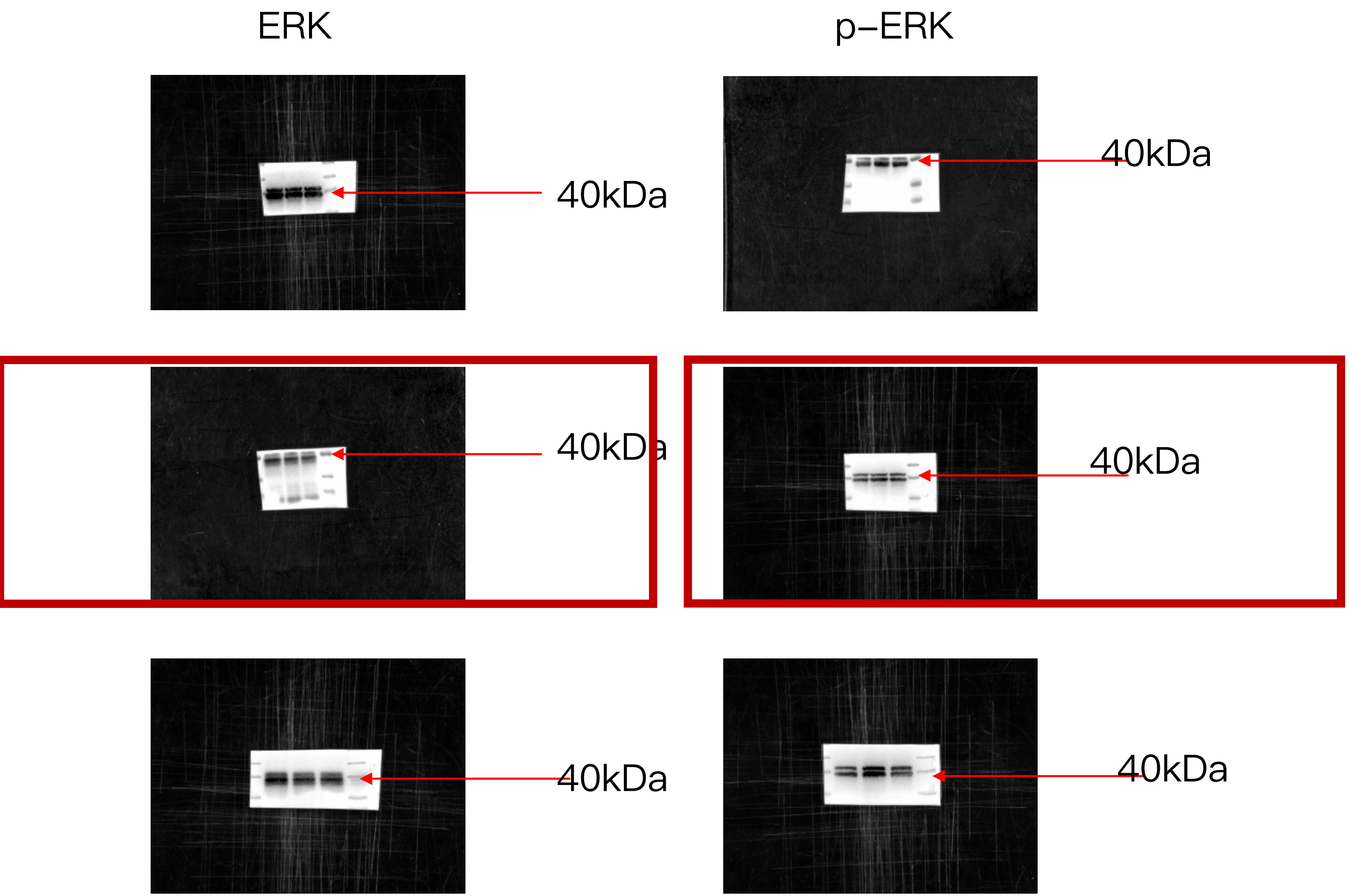

Supplement: Supplementary file 1 [file 12951_2026_4917_MOESM3_ESM.pdf]
