# Supplementary material for: CD63 aptamer–based electrochemical sensor assesses stored red blood cell–derived exosome burden associated with mast-cell–mediated transfusion-related acute lung injury
Source: J Nanobiotechnology. 2026 Aug 12;24:1048. doi: 10.1186/s12951-026-04917-x (PMC13749831; doi:10.1186/s12951-026-04917-x)
Supplement: Supplementary file 2 [file 12951_2026_4917_MOESM2_ESM.docx]

**CD63 Aptamer–Based Electrochemical Sensor Assesses Stored Red Blood Cell–Derived Exosome Burden Associated with Mast-Cell–Mediated Transfusion-Related Acute Lung Injury**

**Supplemental Contents**

**1. Supplemental Figures**

Supplementary Figure S1. Schematic overview of the study design and experimental workflow

Supplementary Figure S2. Specificity controls for CD63 aptamer-based E-AB detection of EXs-RBC

Supplementary Figure S3. Cromolyn pre-administration or mast-cell deficiency diminishes EXs-RBC-induced mast-cell activation and inflammatory lung injury

Supplementary Figure S4. DiI/Rab7 fluorescence colocalization analysis of EXs-RBC in HMC-1 cells

Supplementary Figure S5. RNase pretreatment attenuates EXs-RBC-induced mast-cell activation and TLR3-MAPK signaling in HMC-1 cells

Supplementary Figure S6. Intravenous EXs-RBC administration does not significantly alter pulmonary inflammatory gene expression in healthy mice

**2. Supplemental Tables**

Supplementary Table S1. Information on the reagents used in this study

Supplementary Table S2. Primers used in this study

**3. Supplemental Texts (Methods)**

3.1. Materials and instrumentation

3.2. Detailed cell assay procedures

3.3. Detailed animal experimental procedures

3.4. Detailed biochemical analyses

**1.Supplemental Figures**

**Figure S1. Schematic overview of the study design and experimental workflow.** (A) Construction of the CD63 aptamer-based E-AB sensor. (B) Analytical validation of the CD63 E-AB sensor for rapid assessment of EXs-RBC burden in stored RBC units. (C) Isolation, characterization, and HMC-1 uptake assessment of exosomes from healthy donor plasma and stored RBC units. (D) Exosome-depleted supernatant and EXs-RBC add-back experiments used to assess whether the inflammatory effects were associated with the EXs-RBC-enriched fraction. (E) In vivo evaluation of lung inflammation and injury after EXs-RBC exposure. (F) Pharmacologic mast-cell stabilization and mast-cell-deficient mouse experiments used to assess the contribution of mast cells to EXs-RBC-associated lung injury. (G) RNase pretreatment and DiI/Rab7 colocalization experiments used to probe RNA-sensitive cargo and endosomal access of EXs-RBC. (H) Pharmacologic inhibition experiments used to assess the involvement of TLR3-MAPK signaling. (I) Evaluation of EXs-RBC effects in healthy mice, antibody-independent LPS-induced ARDS-like and sepsis-like models, and an anti-MHC-I antibody-mediated TRALI-like model.

**Supplementary Figure S2. Specificity controls for CD63 aptamer-based E-AB detection of EXs-RBC. (A)** Schematic of the aptamer-dependent specificity-control experiment comparing CD63 aptamer-modified electrodes with electrodes modified with non-CD63 aptamers (Apt-12, Apt-32, and Apt-33) for EXs-RBC burden assessment. **(B, C)** Predicted secondary structures and nucleotide sequences of the CD63 aptamer and non-CD63 aptamers. **(D)** Quantification of current change showing that the CD63 aptamer generated a markedly stronger response to EXs-RBC than the non-CD63 aptamers. **(E)** Schematic of CD63 E-AB detection in PBS, exosome-depleted RBC-unit supernatant (EX-depleted Sup), and EXs-RBC samples. **(F)** Electrochemical current responses showing comparable signals between PBS and EX-depleted Sup, whereas EXs-RBC produced a significantly reduced current response. **(G)** Blood types and storage durations of stored RBC units used for E-AB and NTA analyses. Data are presented as mean ± SEM; exact P values are shown in the panels.

**Supplementary Figure S3. Cromolyn pre-administration or mast-cell deficiency markedly diminishes EXs-RBC–induced mast cell activation and associated inflammatory lung injury.** (A–C) Mice were treated with PBS, EXs-nor, EXs-RBC, and cromolyn plus EXs-RBC. Lungs were collected for analysis (n = 6 per group). (A) ELISA results for TPSAB1 in mouse lungs. (B) ELISA results for IL6, TNFα, CCL2, and CXCL1 in mouse lungs. (C) ELISA results for MPO. (D–E) WT and MC⁻/⁻ mice were treated with EXs-nor or EXs-RBC, and lungs were collected for analysis after treatment (n = 6 per group). (D) ELISA results for IL6, TNFα, CCL2, and CXCL1 in mouse lungs. (E) ELISA results for MPO. Data are expressed as mean ± SEM from three independent experiments; each group had six mice (n = 6); statistical significance was determined by one-way ANOVA followed by Dunnett’s multiple comparisons test. ns, no statistical significance; ***, *P* < 0.001. MC⁻/⁻: mast cell-deficient C57BL/6-Kit^W-sh/W-sh^ mice; WT: wild-type mice.

**Supplementary Figure S4. DiI/Rab7 fluorescence colocalization analysis of EXs-RBC in HMC-1 cells.** DiI-labeled EXs-RBC are shown in red, nuclei were counterstained with DAPI in blue, and Rab7-positive late endosomal compartments are shown in green. Representative merged images show partial overlap of DiI-labeled EXs-RBC signals with Rab7-positive compartments in HMC-1 cells. Boxed regions 1 and 2 in the merged image are shown at higher magnification. Scale bar, 50 μm.

**Supplementary Figure S5. RNase pretreatment attenuates EXs-RBC-induced mast-cell activation, β-Hex A release, inflammatory mediator expression, and TLR3-MAPK signaling in HMC-1 cells. (A)** Experimental workflow for RNase pretreatment of EXs-RBC before HMC-1 stimulation. **(B)** qPCR analysis of TPSAB1 expression. **(C)** ELISA quantification of β-Hex A release in HMC-1 culture supernatants. **(D, E)** qPCR analysis of inflammatory mediator expression, including IL6, TNFα, CCL2, and CXCL1. (F) qPCR analysis of TLR3 expression. **(G, H)** Western blot analysis and quantification of TLR3 protein. **(I)** Western blot analysis and quantification of MAPK phosphorylation. Data are presented as mean ± SEM. Statistical significance was determined by one-way ANOVA followed by post hoc multiple-comparison testing.

**Supplementary Figure S6. Intravenous EXs-RBC administration does not significantly alter pulmonary inflammatory gene expression in healthy mice.** (A) Experimental workflow. Healthy mice received intravenous PBS, EXs-nor, or EXs-RBC in a fixed 50 μL volume; exosome-treated groups received 1.5 × 10⁹ particles/mouse, and lung samples were collected 24 h later. (B) qPCR analysis of pulmonary Tpsab1 expression. (C) qPCR analysis of pulmonary Il6, Tnfα, and Ccl2 expression. (D) qPCR analysis of pulmonary Cxcl1 expression. (E) qPCR analysis of pulmonary Tlr3 expression. Data are presented as mean ± SEM; n = 6 mice per group. Statistical significance was determined by one-way ANOVA followed by post hoc multiple-comparison testing; exact P values are shown in the panels.

**2. Supplemental Tables**

**Supplementary Table S1: Information on the reagents in current study**

| Material | Species | Manufacturer (City, Country) | Catalog / Clone |
| --- | --- | --- | --- |
| anti-TLR3 antibody  Anti-Rab7 antibody  Anti-cd63 antibody  Anti-cd9 antibody | Mouse  Rabbit  Rabbit  Rabbit | Abcam (Cambridge, UK)  HUABIO(Hangzhou,China)  HUABIO(Hangzhou,China)  HUABIO(Hangzhou,China) | ab13915; Clone 40c1285  ET1611-96;  HA722731;  HA721533; |
| anti-TPSAB1 antibody | Rabbit | HUABIO (Hangzhou, China) | ET1610-64; Clone SC68-07 |
| anti-CD81 antibody | Rabbit | HUABIO (Hangzhou, China) | ET1611-87 |
| anti-ALIX antibody  Anti-Calnexin antibody | Rabbit  Rabbit | HUABIO (Hangzhou, China)  Promab (Changsha,China) | ET1705-74  P23554 |
| anti-α-tubulin antibody  Anti-TSG101 antibody | Mouse  Rabbit | Proteintech (Wuhan, China)  Proteintech (Wuhan, China) | 11224-1-AP  28283-1-AP |
| Phospho-JNK antibody | Rabbit | HUABIO (Hangzhou, China) | ET1609-42; Clone ST500 |
| Phospho-p38 antibody | Rabbit | Cell Signaling Technology (Danvers, MA, USA) | #4511; Clone D3F9 |
| Phospho-ERK1/2 antibody | Rabbit | Cell Signaling Technology (Danvers, MA, USA) | #4370; Clone D13.14.4E |
| Total p38 antibody | Rabbit | Proteintech (Wuhan, China) | 14064-1-AP |
| Total ERK1/2 antibody | Rabbit | Cell Signaling Technology (Danvers, MA, USA) | #4695; Clone 137F5 |
| Total JNK antibody | Rabbit | Abcam (Cambridge, UK) | ab244205 |
| HRP-goat anti-rabbit IgG | Goat | ZSGB-BIO (Beijing, China) | ZB-2301 |
| HRP-goat anti-mouse IgG | Goat | ZSGB-BIO (Beijing, China) | ZB-5305 |
| LPS (E. coli) | — | Solarbio Life Sciences (Beijing, China) | L8880 |
| Cromolyn sodium | — | GLPBIO (Montclair, CA, USA) | GC76086 |
| CU-CPT-4a (TLR3/dsRNA complex inhibitor) | — | GLPBIO (Montclair, CA, USA) | GC11461 |
| CC-401 (JNK inhibitor) | — | GLPBIO (Montclair, CA, USA) | GC14197 |
| SCH772984 (ERK1/2 inhibitor) | — | GLPBIO (Montclair, CA, USA) | GC16001 |
| TAK-715 (p38 inhibitor) | — | GLPBIO (Montclair, CA, USA) | GC16543 |
| DiI [DiIC18(3)] | — | MCE (Monmouth Junction, NJ, USA) | HY-D0083 |
| ELISA kit: CXCL1 (Mouse) | Mouse | Ruixin biotech(quan zhou,China) | Cat:RXW203185M |
| ELISA kit: CCL2 (Mouse) | Mouse | Ruixin Biotech (Quanzhou, China) | RX202682M |
| ELISA kit: IL-6 (Mouse) | Mouse | Elabscience (Wuhan, China) | E-EL-M0044c |
| ELISA kit: TNF-α (Mouse) | Mouse | Elabscience (Wuhan, China) | E-EL-M0002 |
| ELISA kit: TPSAB1 (Mouse)  ELISA kit: β-Hex A (Mouse) | Mouse  Human‌ | Ruixin Biotech (Quanzhou, China)  Ruixin Biotech (Quanzhou, China) | RX202771M  RX101737H |
| ELISA kit: TLR3 (Mouse) | Mouse | Ruixin Biotech (Quanzhou, China) | RX203136M |
| ELISA kit: MPO (Mouse) | Mouse | MBbiology (Jiangsu, China) | MB-3074A |

*Other reagents were of analytical grade and obtained from Sigma-Aldrich (St. Louis, MO, USA)

**Supplementary Table S2:The primers used in our study.**

| **Target (Species)** | **Forward (5’→3’)** | | **Reverse (5’→3’)** |
| --- | --- | --- | --- |
| TLR3-Human | | GCCTCTTCGTAACTTGACCATTCTG | ACCAGGGTTTGCGTGTTTCCAG |
| *Tlr3*-Mouse | | ACTGGGTCTGGGAACATTTCTC | AGGACGCCTGCTTCAAAGTC |
| TPSAB1-Human | | GACAGGCTGGGGTAACATCG | GAAGGCAGAAGAATTGGGACTC |
| *Tpsab1*-Mouse | | GACAGGCTGGGGTAACATCG | GAAGGCAGAAGAATTGGGACTC |
| IL6-Human | | CAATATTAGAGTCTCAACCCCCAA | TCACCAGGCAAGTCTCCTCA |
| *Il6*-Mouse | | GGAGCCCACCAAGAACGATAGTC | CACCAGCATCAGTCCCAAGAAGG |
| TNFα-Human | | CTCGAACCCCGAGTGACAAG | TGAGGTACAGGCCCTCTGAT |
| *Tnfα*-Mouse | | GGACTAGCCAGGAGGGAGAACAG | GCCAGTGAGTGAAAGGGACAGAAC |
| CCL2-Human | | GATCTCAGTGCAGAGGCTCG | TTTGCTTGTCCAGGTGGTCC |
| *Ccl2*-Mouse | | TTCCACAACCACCTCAAGCACTTC | ATTAAGGCATCACAGTCCGAGTCAC |
| CXCL1-Human | | CGCCTCTGATCCAAGCCAC | CTGCACATAGTCCTGCACCA |
| *Cxcl1-*Mouse | | GGCTGGGATTCACCTCAAGAACATC | AGTGTGGCTATGACTTCGGTTTGG |

The primers used for qPCR in our study.

**3. Supplemental Texts (Methods)**

**3.1.Materials and instrumentation**

6-Mercapto-1-hexanol (MCH, ≥97%) and tris(2-carboxyethyl)phosphine hydrochloride (TCEP ≥98%) were purchased from Aladdin (China). Phosphate-buffered saline (PBS; 1×, pH 7.4) and ultrapure water (18.2 MΩ·cm) were used throughout. Gold disk electrodes (2.0 mm diameter) were obtained from Nanjing Jinhuice Instrument Technology Co., Ltd. (China). Electrochemical measurements were performed on a 1030C electrochemical workstation (CH Instruments, China) using a three-electrode configuration with a gold working electrode, an Ag/AgCl (3 M KCl) reference electrode, and a platinum wire counter electrode. Circular dichroism (CD) spectra were recorded on a J-1500 spectropolarimeter (JASCO, Japan). Field-emission scanning electron microscopy (FE-SEM; FEI, USA) was used as indicated. The CD63 aptamer (Apt-63) bearing a 5′ disulfide-protected thiol (C6 linker) was synthesized and HPLC-purified by Sangon Biotech (China);

Sequence: 5′-HS-C6-CACCCCACCTCGCTCCCGTGACACTAATGCTA-MB-3′.

**Electrode preparation and sensor assembly.** Gold disks were mechanically polished with 0.05 µm alumina, sonicated in water and ethanol, and electrochemically cleaned by cyclic voltammetry (CV) in 0.5 M NaOH (−1.35 to −0.35 V, 2.0 V·s⁻¹, 20 cycles), followed by CV in 0.5 M H₂SO₄ (0.2–1.4 V vs Ag/AgCl, 0.1 V·s⁻¹, 20 cycles), and then rapid scans at 2.0 V·s⁻¹ until stable voltammograms were obtained. The disulfide-protected aptamer was reduced with 10 mM TCEP in PBS (60 min, room temperature, dark) and desalted (spin column) to remove residual reductant. The aptamer was pre-folded in PBS containing 2 mM MgCl₂ (95 °C for 5 min, ice for 5–10 min, then 10 min at room temperature) and self-assembled onto freshly cleaned gold (1–4 µM, 2 h at RT or overnight at 4 °C, dark). Electrodes were then backfilled with 1–3 mM MCH (in PBS, 1–2 h, RT, dark), rinsed, and stored in PBS.

**Electrochemical characterization and detection.** All electrochemical measurements were performed on a 1030C electrochemical workstation (China Instrumentation Inc.). A standard three-electrode system was used, consisting of a CD63 aptamer-modified gold working electrode, an Ag/AgCl reference electrode, and a platinum counter electrode. Square wave voltammetry (SWV) was applied to record peak currents in PBS and exosome-containing samples. Frequency scans were carried out from 5 to 300 Hz, and 200 Hz was selected for subsequent measurements to ensure signal stability and reproducibility. Dose–response experiments were performed by incubating the electrodes with different concentrations of EXs-RBC, and the corresponding SWV peak currents were recorded. The signals were expressed as relative changes compared with the post-MCH baseline according to the following equation:

Signal gain (S) = (I_blank_ -I_target_) / I_blank_ × 100%

where I_blank_ and I_target_ represent the peak currents in the absence and presence of exosomes, respectively.

**Exosome assay workflow and validation.** To establish detectability and dose–response, exosomes isolated from EXs-RBC were spiked into PBS at three NTA-calibrated particle concentrations (3.0 × 10¹⁰, 9.0 × 10¹⁰, and 15.0 × 10¹⁰ particles/mL) and measured on the sensor. To assess the correlation between E-AB sensor output and exosome particle concentration, stored RBC-unit samples were measured directly using the CD63 E-AB sensor, whereas EXs-RBC isolated from matched samples were quantified by NTA. NTA-measured particle concentrations were first log_10_-transformed and then normalized using the min–max method. Electrochemical current signals were normalized using the same method. Linear regression analysis was subsequently performed using the normalized datasets. To evaluate the effect of the RBC matrix on sensor performance, pre-quantified EXs-RBC suspensions with particle concentrations of 3.0 × 10¹⁰, 6.0 × 10¹⁰, and 12 × 10¹⁰ particles/mL were spiked into PBS-diluted RBC suspensions. All samples were analyzed under identical incubation and electrochemical conditions. To verify that the electrochemical signals were primarily associated with the EXs-RBC fraction, three sample types were analyzed: (i) PBS as the blank control, (ii) isolated EXs-RBC as the positive control, and (iii) exosome-depleted RBC-unit supernatant (EX-depleted Sup). All samples were adjusted to the same final volume and analyzed using the same electrochemical protocol. For specificity evaluation, four aptamer-modified electrode configurations were fabricated under identical conditions, including one CD63 aptamer-modified electrode and three non-CD63 aptamer-modified electrodes (Apt-12, Apt-32, and Apt-33). All aptamers contained the same 5′SH-(CH₂)₆ anchoring group and 3′MB redox reporter. Electrodes were incubated with the same EXs-RBC sample for 30 min at room temperature before SWV measurement, and electrochemical responses were compared to evaluate the specificity of the CD63 aptamer.

The sequences of the non-CD63 aptamers were as follows:

Apt-12:

5′-SH-(CH₂)₆-GGGGGTGCAGGCATCAACTAGCATTTTT-MB-3′

Apt-32:

5′-SH-(CH₂)₆-AGACAAGGAAAATCCTTCAATGAAGTGGGTCT-MB-3′

Apt-33:

5′-SH-(CH₂)₆-ACGTTTCGCATGAGAAACGTATGGTTTGAAGGT-MB-3′

**3.2. Detailed cell assays procedures**

**Cell culture, exosome isolation, and characterization**

HMC-1 cells were obtained from the Shanghai Cell Bank of the Chinese Academy of Sciences (Shanghai, China) and cultured in RPMI-1640 (Gibco, Cat. No. 8123259, USA) supplemented with 100 µg mL⁻¹ streptomycin, 100 U mL⁻¹ penicillin, and 10% heat-inactivated fetal bovine serum (FBS). Cells were maintained at 37 °C in a humidified atmosphere containing 5% CO₂. Exosomes were isolated by sequential differential centrifugation and ultracentrifugation at 4 °C from platelet-free plasma of healthy donors or supernatants from stored RBC units. Samples were centrifuged at 450 × g for 10 min to remove residual cells, 2,000 × g for 10 min to remove cell debris and large particles, and 10,000 × g for 10 min to deplete apoptotic bodies and large vesicles, followed by PBS washing and ultracentrifugation at 100,000 × g for 90 min to collect the EXs-enriched pellet and reduce soluble co-isolates. The final EXs pellet was resuspended in PBS. Particle concentration and size distribution were determined by NTA after dilution and back-calculated to the original EXs stock.

Exosomes were characterized by transmission electron microscopy (TEM), nanoparticle tracking analysis (NTA), and Western blotting. For TEM, samples were negatively stained with 3% uranyl acetate for 1 min and examined on an HT-7700 TEM (Hitachi, Tokyo, Japan). For NTA, 50 µL of exosomes was diluted in 50 mL PBS (1:1000) and analyzed on a ZetaView instrument (Particle Metrix, Munich, Germany). For Western blotting, exosomes were lysed in RIPA buffer, separated by SDS-PAGE (12% gels), and probed with primary antibodies against CD63, CD9, CD81, TSG101, and ALIX, with calnexin assessed as a cellular contamination marker.

**Exosome-depleted supernatant and EXs-RBC add-back control experiments**

To distinguish the effects of EXs-RBC from soluble components remaining in stored RBC-unit supernatants, exosome-depleted supernatant and add-back control experiments were performed. After ultracentrifugation, the post-ultracentrifugation supernatant was carefully collected without disturbing the exosome-enriched pellet and was defined as exosome-depleted RBC-unit supernatant (EX-depleted Sup).

For these experiments, concentrated EXs-RBC suspensions were prepared at approximately 6 × 10¹⁰ particles/mL to maintain a fixed 50 µL treatment volume. Thus, 25 µL concentrated EXs-RBC contained approximately 1.5 × 10⁹ particles. Four treatment mixtures were prepared immediately before use: PBS group, 50 µL PBS; EXs-RBC group, 25 µL concentrated EXs-RBC plus 25 µL PBS; EX-depleted Sup group, 25 µL EX-depleted Sup plus 25 µL PBS; and EX-depleted Sup + EXs-RBC group, 25 µL concentrated EXs-RBC plus 25 µL EX-depleted Sup.

For HMC-1 cells, each 50-µL treatment mixture was added to 950 µL culture medium, yielding a final volume of 1 mL. For mouse experiments, the same 50 µL treatment mixtures were administered by intratracheal instillation.

**Exosome labeling with DiI and uptake assay**

To evaluate the in vitro uptake of exosomes by HMC-1 cells, purified exosomes were labeled with the fluorescent lipophilic dye DiI using the DiI (DiIC18(3)) kit according to the manufacturer’s instructions. Briefly, exosomes were incubated with DiI at a final dye concentration of 10 µM for 15 min at room temperature. After staining, unincorporated dye was removed by ultracentrifugation at 100,000 × g for 2 h at 4 °C. The labeled exosomes were then incubated with HMC-1 cells at the indicated dose for 24 h at 37 °C. Following incubation, cells were washed twice with PBS and fixed in 4% paraformaldehyde for 15 min at room temperature. Cellular uptake of exosomes was visualized using an automated inverted fluorescence microscope (Olympus, Tokyo, Japan). For immunofluorescence staining, cells were permeabilized with 0.2% Triton X-100 for 10 min and washed with PBS. After blocking with 5% BSA for 30–60 min at room temperature, cells were incubated with anti-Rab7 primary antibody at 4°C overnight. The next day, cells were washed with PBS and incubated with fluorescence-conjugated secondary antibody for 60 min at room temperature under light-protected conditions. Cell nuclei were stained with DAPI for 5 min, followed by PBS washing. Finally, colocalization of exosomes with Rab7-positive endosomes was visualized using a confocal laser scanning microscope.

**Co-culture of HMC-1 cells with plasma-derived and RBC-derived exosomes**

Prepared HMC-1 cells were plated in 1 mL of exosome-depleted complete RPMI-1640 and exposed at 37 °C in 5% CO₂ to one of three conditions: (i) PBS vehicle, (ii) plasma-derived exosomes from healthy donors (EXs-nor), or (iii) RBC-derived exosomes from stored units (EXs-RBC). Exosome treatments were performed at 1.5 × 10⁹ particles/mL (corresponding to approximately 50 µg exosome protein/mL), with PBS added to maintain a 1 mL final volume. After 6 h, cells and conditioned media (supernatants) were collected for downstream analyses.

Exosome-depleted supernatant and add-back experiments

**Cromolyn pretreatment and exosome exposure in HMC-1 cells.**

To assess whether cromolyn attenuates mast-cell activation and inflammatory responses upon exosome exposure, HMC-1 cells were cultured in exosome-depleted complete RPMI-1640 (1 mL per well, 37 °C, 5% CO₂) and assigned to four groups, with PBS added to maintain a 1 mL final volume per well. For these experiments, EXs-nor and EXs-RBC suspensions were prepared at approximately 3 × 1010 particles/mL to maintain a fixed 50 µL treatment volume. Thus, 50 µL concentrated EXs-RBC contained approximately 1.5 × 10^9^ particles, corresponding to 50 µg total exosome protein.

(i) PBS control: 100 µL PBS, followed 1 h later by 100 µL PBS.

(ii) EXs-nor: 100 µL PBS, followed 1 h later by EXs-nor at 1.5 × 10⁹ particles/mL.

(iii) EXs-RBC: 100 µL PBS, followed 1 h later by EXs-RBC at 1.5 × 10⁹ particles/mL.

(iv) Cromolyn-pretreated + EXs-RBC: cromolyn sodium (50 µM) for 1 h, followed by EXs-RBC at 1.5 × 10⁹ particles/mL.

After 6 h of exosome exposure, cells and conditioned media (supernatants) were collected for downstream analyses.

**In vitro pharmacologic modulation of the TLR3/MAPK axis.**

To investigate the involvement of TLR3 and MAPK in EXs-RBC–induced mast-cell activation, HMC-1 cells were cultured in exosome-depleted complete RPMI-1640 (1 mL per well; 37 °C, 5% CO₂) and assigned to the following groups (PBS added to maintain a 1 mL final volume). For these experiments, EXs-nor and EXs-RBC suspensions were prepared at approximately 3 × 10^10^ particles/mL to maintain a fixed 50 µL treatment volume. Thus, 50 µL concentrated EXs-RBC contained approximately 1.5 × 10^9^ particles.

(i) PBS control: 50 µL PBS.

(ii) EXs-nor: 50 µL EXs-nor at 1.5 × 10⁹ particles/mL (final).

(iii) EXs-RBC: 50 µL EXs-RBC at 1.5 × 10⁹ particles/mL (final).

(iv) CU-CPT-4a + EXs-RBC: 5 µM CU-CPT-4a(TLR3 antagonist ;1 h pretreatment), followed by 50 µL EXs-RBC.

(v) CC-401 + EXs-RBC: 5 µM CC-401 (JNK inhibitor; 1 h pretreatment), followed by 50 µL EXs-RBC.

(vi) TAK-715 + EXs-RBC: 5 µM TAK-715 (p38 inhibitor; 1 h pretreatment), followed by 50 µL EXs-RBC.

(vii) SCH772984 + EXs-RBC: 5 µM SCH772984 (ERK1/2 inhibitor; 1 h pretreatment), followed by 50 µL EXs-RBC.

After exosome exposure (6 h), cells and conditioned media (supernatants) were collected for downstream analyses.

**RNase A pretreatment of EXs-RBC**

To assess whether RNA-sensitive vesicular cargo contributes to EXs-RBC-induced mast-cell activation, EXs-RBC were pretreated with RNase A before stimulation of HMC-1 cells. Briefly, the EXs-RBC pellet was resuspended in RNase-free PBS, and RNase A was added to a final concentration of 100 µg/mL. After gentle mixing, samples were incubated at 37 °C for 60 min, followed by two washes using 100 kDa molecular weight cut-off (MWCO) ultrafiltration tubes (3000 × g) to completely remove residual RNase A and degraded RNA fragments. The recovered EXs-RBC were resuspended in PBS and used for subsequent experiments. HMC-1 cells were cultured in exosome-depleted complete RPMI-1640 (1 mL per well; 37 °C, 5% CO₂) and assigned to the following groups (PBS added to maintain a 1 mL final volume). EXs-RBC were applied at a final concentration 1.5 × 10⁹ particles/mL (corresponding to approximately 50 µg exosome protein/mL):

(i) PBS control: 50 µL PBS.

(ii) EXs-RBC: 50 µL EXs-RBC (1.5 × 10⁹ particles).

(iii) EXs-RBC+ RNase: 50 µL RNase A-pretreated EXs-RBC (1.5 × 10⁹ particles).

After 6 h of exosome exposure, cells and conditioned media (supernatants) were collected for downstream analyses.

**3.3. Detailed animal experiment procedures**

**Animals**

Male BALB/c mice (20–22 g; 8–12 weeks of age) were supplied by DOSSY Experimental Animals Co., Ltd. (Chengdu, China) and were used for the antibody-mediated TRALI-like model, LPS-induced ARDS model, sepsis model, and EXs-RBC administration experiments. Male mast cell-deficient C57BL/6-Kit^W-sh/W-sh^ mice and their wild-type littermates (20–22 g; 8–12 weeks of age) were obtained from Cyagen Biosciences (Jiangsu, China; Stock No. C001378) and were used specifically for experiments assessing the role of mast-cell deficiency. All animals were housed under standard conditions at the Experimental Animal Center of Fujian Provincial Hospital. Prior to experimental procedures, mice were fasted for 8 h with free access to water. Animals were randomized to experimental groups (n=6 per group) using R software, with an additional 10% pre-randomized and held in reserve to accommodate potential attrition. Any animals that met prespecified exclusion criteria were replaced according to a predefined randomization sequence.

**Intratracheal, intraperitoneal, and tail vein injection protocols**

Mice were anesthetized by intraperitoneal injection of sodium pentobarbital (50 mg kg⁻¹; 1% w/v). Following anesthesia, animals received interventions via the indicated routes according to the experimental design: intratracheal instillation (50 µL), intraperitoneal injection (50 µL), or tail-vein injection according to the experimental design. Mice subjected to intratracheal instillation underwent endotracheal intubation and were mechanically ventilated for 5 min post-procedure. All animals were then placed on a warming pad until recovery.

**Part 1. Exosome-depleted supernatant and EXs-RBC add-back control experiments**

To determine whether the observed pulmonary inflammatory responses were mediated by EXs-RBC, exosome-depleted supernatant and EXs-RBC add-back experiments were performed in mice. After ultracentrifugation, the supernatant was carefully collected without disturbing the EXs-enriched pellet and was defined as exosome-depleted RBC supernatant (EX-depleted Sup). Concentrated EXs-RBC suspensions were prepared at approximately 6 × 10¹⁰ particles/mL. Thus, 25 µL of concentrated EXs-RBC contained approximately 1.5 × 10⁹ particles. Four 50 µL treatment preparations were prepared immediately before use:

(i) PBS control: 50 µL PBS (i.t.).

(ii) EXs-RBC: 25 µL EXs-RBC plus 25 µL PBS (i.t.).

(iii) EX-depleted Sup: 25 µL EX-depleted Sup plus 25 µL PBS (i.t.).

(iv) EX-depleted Sup + EXs-RBC: 25 µL EX-depleted Sup plus 25 µL EXs-RBC (i.t.).

At 24 h after intratracheal instillation, mice were euthanized by intraperitoneal overdose of sodium pentobarbital (100 mg kg⁻¹), and samples were collected for analysis.

**Part 2. Intratracheal dose–response of EXs-RBC**

To investigate dose-dependent effects of airway delivery of EXs-RBC on mast-cell activation, lung inflammation, and injury, mice received intratracheal instillation (i.t.) of 50 µL per mouse under anesthesia. EXs-nor and standard-dose EXs-RBC suspensions were prepared at approximately 3 × 10¹⁰ particles/mL, corresponding to approximately 1.5 × 10⁹ particles in 50 µL. High-dose EXs-RBC suspensions were prepared at approximately 6 × 10¹⁰ particles/mL, corresponding to approximately 3 × 10⁹ particles in 50 µL. Groups (all volume-matched to 50 µL):

(i) PBS control: 50 µL PBS (i.t.).

(ii) EXs-nor standard dose: 50 µL EXs-nor (1.5 × 10⁹ particles) (i.t.).

(iii) EXs-RBC standard dose: 50 µL EXs-RBC (1.5 × 10⁹ particles) (i.t.).

(iv) EXs-RBC high dose: 50 µL EXs-RBC (3 × 10⁹ particles) (i.t.).

At 24 h after intratracheal instillation, mice were euthanized by intraperitoneal overdose of sodium pentobarbital (100 mg kg⁻¹), and samples were collected for analysis.

**Part 3.** **Effect of cromolyn pre-administration in the EXs-RBC i.t. lung-injury model**

Under anesthesia, mice received intraperitoneal (i.p.) pre-administration, followed 1 h later by intratracheal instillation (i.t.). EXs-nor and EXs-RBC suspensions were prepared at approximately 3 × 10¹⁰ particles/mL, and each mouse received approximately 1.5 × 10⁹ particles in a total volume of 50 µL.

(i) PBS control (PBS, then PBS): i.p. 50 µL PBS; 1 h later, i.t. 50 µL PBS.

(ii) EXs-nor (PBS, then EXs-nor): i.p. 50 µL PBS; 1 h later, i.t. 50 µL EXs-nor.

(iii) EXs-RBC (PBS, then EXs-RBC): i.p. 50 µL PBS; 1 h later, i.t. 50 µL EXs-RBC.

(iv) Cromolyn-pretreated + EXs-RBC: i.p. cromolyn sodium (50 mg/ kg) in 50 µL; 1 h later, i.t. 50 µL EXs-RBC.

At 24 h after intratracheal instillation, mice were euthanized by intraperitoneal overdose of sodium pentobarbital (100 mg kg⁻¹), and samples were collected for analysis.

**Part 4:** **Effect of mast-cell deficiency on EXs-RBC–induced lung inflammation/injury in vivo**

Male C57BL/6-Kit^W-sh/W-sh^ (mast-cell–deficient) mice and wild-type littermates were enrolled. Under anesthesia, mice received intratracheal instillation (i.t.). EXs-nor and EXs-RBC suspensions were prepared at approximately 3 × 10¹⁰ particles/mL, and each mouse received approximately 1.5 × 10⁹ particles in a total volume of 50 µL.

(i) EXs-nor standard dose (1.5 × 10⁹ particles): i.t. 50 µL EXs-nor.

(ii) EXs-RBC standard dose (1.5 × 10⁹ particles): i.t. 50 µL EXs-RBC.

At 24 h after intratracheal instillation, mice were euthanized by intraperitoneal overdose of sodium pentobarbital (100 mg kg⁻¹), and samples were collected for analysis.

**Part 5:** **Pharmacologic inhibition of the TLR3/MAPK axis in vivo**

Under anesthesia, mice received intraperitoneal (i.p.) pretreatment, followed 1 h later by intratracheal instillation (i.t.). EXs-nor and EXs-RBC suspensions were prepared at approximately 3 × 10¹⁰ particles/mL, and each mouse received approximately 1.5 × 10⁹ particles in a total volume of 50 µL. Vehicles were volume-/solvent-matched across groups.

(i) PBS control: i.p. 50 µL PBS; 1 h later, i.t. 50 µL PBS.

(ii) EXs-nor standard dose (1.5 × 10⁹ particles): i.p. 50 µL PBS; 1 h later, i.t. 50 µL EXs-nor.

(iii) EXs-RBC standard dose (1.5 × 10⁹ particles): i.p. 50 µL PBS; 1 h later, i.t. 50 µL EXs-RBC.

(iv) CU-CPT-4a + EXs-RBC: i.p. CU-CPT-4a (10 mg kg⁻¹); 1 h later, i.t. 50 µL EXs-RBC.

(v) CC-401 + EXs-RBC: i.p. CC-401 (10 mg kg⁻¹; JNK inhibitor); 1 h later, i.t. 50 µL EXs-RBC.

(vi) TAK-715 + EXs-RBC: i.p. TAK-715 (10 mg kg⁻¹; p38 inhibitor); 1 h later, i.t. 50 µL EXs-RBC.

(vii) SCH772984 + EXs-RBC: i.p. SCH772984 (10 mg kg⁻¹; ERK1/2 inhibitor); 1 h later, i.t. 50 µL EXs-RBC.

At 24 h after intratracheal instillation, mice were euthanized by intraperitoneal overdose of sodium pentobarbital (100 mg kg⁻¹), and samples were collected for analysis.

**Part 6: Differential effects of EXs-RBC across unprimed, ARDS, and sepsis mice.**

**General procedures.** Under anesthesia, all exosome/PBS boluses were delivered by tail-vein (i.v.) injection. EXs-nor and EXs-RBC suspensions were prepared at approximately 3 × 10¹⁰ particles/mL, and each mouse received approximately 1.5 × 10⁹ particles in a total volume of 50 µL. Vehicles were volume-/solvent-matched across groups.

**Unprimed mice — i.v. bolus only**

(i) PBS control: 50 µL PBS (i.v.).

(ii) EXs-nor standard dose (1.5 × 10⁹ particles): 50 µL EXs-nor (i.v.).

(iii) EXs-RBC standard dose (1.5 × 10⁹ particles): 50 µL EXs-RBC (i.v.).

**LPS-induced ARDS model — i.t. priming + i.v. bolus**

(i) Sham: no treatment.

(ii) PBS + PBS: 50 µL PBS (i.t.), then 1 h later 50 µL PBS (i.v.).

(iii) LPS: 50 µL of 1 µg µL⁻¹ LPS (i.t.; 50 µg total), then 1 h later 50 µL PBS (i.v.).

(iv) LPS + EXs-nor standard dose (1.5 × 10⁹ particles): i.t. LPS as in (iii), then 1 h later 50 µL EXs-nor (i.v.).

(v) LPS + EXs-RBC standard dose (1.5 × 10⁹ particles): i.t. LPS as in (iii), then 1 h later 50 µL EXs-RBC (i.v.).

**LPS-induced sepsis model — i.p. priming + i.v. bolus**

(i) Sham: no treatment.

(ii) PBS + PBS: 50 µL PBS (i.p.), then 1 h later 50 µL PBS (i.v.).

(iii) LPS: 50 µL of 3 µg µL⁻¹ LPS (i.p.; 150 µg total), then 1 h later 50 µL PBS (i.v.).

(iv) LPS + EXs-nor standard dose (1.5 × 10⁹ particles): i.p. LPS as in (iii), then 1 h later 50 µL EXs-nor (i.v.).

(v) LPS + EXs-RBC standard dose (1.5 × 10⁹ particles): i.p. LPS as in (iii), then 1 h later 50 µL EXs-RBC (i.v.).

**Sample collection.** Mice were euthanized 24 h after the tail-vein (i.v.) bolus injection (PBS or exosomes). Sham mice were euthanized at the matched time point.

**Part 7: TRALI-like model with EXs-RBC challenge (LPS priming + anti–MHC-I + i.v. exosome bolus).**
**General procedures.** To assess the effects of EXs-RBC on pulmonary inflammatory responses and mast-cell activation in a TRALI-like setting, mice were primed with LPS (0.1 mg/kg, i.p.; *Escherichia coli*, L8880; Solarbio) for 24 h. Mice were then challenged with an anti–MHC-I monoclonal antibody (mAb; 1 mg/kg in 100 µL PBS) via tail-vein (i.v.) injection. One hour later, EXs-nor and EXs-RBC suspensions were prepared at approximately 3 × 10¹⁰ particles/mL, and each mouse received approximately 1.5 × 10⁹ particles in a total volume of 50 µL. Vehicles were volume-matched across groups. The TRALI model was established as previously described and confirmed in preliminary experiments.

**TRALI-like model — LPS priming + anti–MHC-I challenge + i.v. bolus**
(i) TRALI + PBS: LPS (0.1 mg/kg, i.p.), then 24 h later anti–MHC-I mAb (1 mg/kg in 100 µL PBS, i.v.), then 1 h later PBS (50 µL, i.v.).
(ii) TRALI + EXs-nor standard dose (1.5 × 10⁹ particles): i.p. LPS as in (i), then anti–MHC-I mAb as in (i), then 1 h later EXs-nor (50 µL, i.v.).
(iii) TRALI + EXs-RBC standard dose (1.5 × 10⁹ particles): i.p. LPS as in (i), then anti–MHC-I mAb as in (i), then 1 h later EXs-RBC (50 µL, i.v.).

**Sample collection.** Mice with TRALI were euthanized 2 h after the anti–MHC-I challenge, and samples (lungs and bronchoalveolar lavage fluid) were collected for subsequent analyses.

**3.4: Detailed biochemical analyses**

**qPCR**

Lung tissues and cultured cells were harvested and homogenized in TRIzol reagent. Total RNA was isolated with TRIzol, and reverse transcription was performed using a cDNA synthesis kit (Bio-Rad). qPCR was carried out with Maxima™ SYBR Green qPCR Master Mix (Thermo Fisher Scientific), and expression levels were normalized to 18S rRNA. Relative expression was calculated using the 2^ΔΔCt^ method. Primer sequences are provided in **Supplementary Table S2**.

**ELISA**

ELISA was performed to determine the levels of TPSAB1, IL-6, TNF-α, CCL2, CXCL1, TLR3, β-Hexosaminidase A, and MPO in lung homogenates and cell-culture supernatants. Results are presented as fold-change relative to the sham or PBS control group.

**Western blot analysis**

Mouse lung tissues and cultured cells were lysed in RIPA buffer supplemented with protease and phosphatase inhibitors. Protein concentration was determined using the Pierce™ BCA Protein Assay Kit (Thermo Fisher Scientific). Equal amounts of protein were separated by SDS-PAGE (12% gels), transferred to PVDF membranes, blocked with 5% nonfat dry milk in TBST, and incubated overnight at 4 °C with the appropriate primary antibodies. After washing, membranes were incubated with HRP-conjugated secondary antibodies; signals were detected with ECL and captured using a chemiluminescence imaging system. Protein expression was quantified by densitometry and normalized to α-tubulin.

For exosome characterization, EX preparations were lysed in RIPA buffer and analyzed by western blotting for exosome-associated markers, including CD63, CD9, CD81, TSG101, and ALIX, with calnexin assessed as a cellular contamination marker.

**H&E**

Mouse lung tissues were fixed in 10% neutral buffered formalin (NBF), processed, embedded in paraffin, and sectioned at 2 µm. Sections were deparaffinized and rehydrated, stained with hematoxylin for 5 min, blued, counterstained with eosin for 1 min, and dehydrated through graded ethanol before clearing and mounting. Images were acquired at 200× magnification using a bright-field microscope (Zeiss, Germany). Histological evaluation was performed in a blinded manner.

**Lung wet-to-dry weight ratio**
The lung wet-to-dry weight ratio was determined using the right cranial lung lobe. Briefly, the right cranial lobe was excised immediately after euthanasia, gently blotted to remove surface fluid, and weighed to obtain the wet weight. Samples were then dried at 60°C until a constant weight was reached and weighed again to obtain the dry weight. The wet-to-dry ratio was calculated as wet weight divided by dry weight.

**BALF total protein measurement**
Bronchoalveolar lavage fluid (BALF) was collected from mice after euthanasia. BALF samples were centrifuged to remove cellular debris, and the supernatants were collected for total protein measurement. BALF total protein concentrations were determined using a BCA protein assay according to the manufacturer’s instructions.

**H&E staining and histological lung injury scoring**
Mouse lung tissues were fixed in 10% neutral buffered formalin (NBF), processed, embedded in paraffin, and sectioned at 2 µm. Sections were deparaffinized and rehydrated, stained with hematoxylin for 5 min, blued, counterstained with eosin for 1 min, and dehydrated through graded ethanol before clearing and mounting. Images were acquired at 200× magnification using a bright-field microscope (Zeiss, Germany). Histological lung injury was quantified in a blinded manner using an established acute lung injury scoring system as previously described. Lung injury was assessed using a semi-quantitative scoring system based on four parameters: alveolar congestion, hemorrhage, inflammatory cell infiltration in the alveolar spaces or vessel walls, and alveolar wall thickening/hyaline membrane formation. Each parameter was scored from 0 to 4 according to the affected area: 0, absent; 1, <25%; 2, 25–50%; 3, 50–75%; and 4, >75%. The total lung injury score for each H&E-stained field was calculated as the sum of the four parameters, with a maximum score of 16. Six H&E-stained fields from each group were evaluated in a blinded manner and used for histological score analysis.
